# Hetero Diels–Alder Cycloaddition of Siloxy Vinylallenes—Synthesis of the Indolizidine Skeleton: Experimental and Computational Studies

**DOI:** 10.3390/molecules30234627

**Published:** 2025-12-02

**Authors:** Juan Francisco Rodríguez-Caro, Gabriel Vargas-Arana, María del Mar Afonso, José Antonio Palenzuela

**Affiliations:** 1 Departamento de Química Orgánica, Instituto Universitario de Bio-Orgánica Antonio González (SINTESTER), Universidad de La Laguna, Avda. Astrofísico Fco. Sánchez 2, 38206 La Laguna, Spain; jfrodcaro@gmail.com (J.F.R.-C.); gvargas@iiap.gob.pe (G.V.-A.); 2 Laboratorio de Química de Productos Naturales, Instituto de Investigaciones de la Amazonía Peruana, Avenue Abelardo Quiñones km 2.5, Iquitos 16001, Peru

**Keywords:** hetero Diels–Alder, vinylallenes, computational calculations

## Abstract

Vinylallenes have been used in Diels–Alder reactions with a variety of dienophiles. However, vinylallenes activated at the allenic part of the molecule have been reacted only with carbon–carbon double bonds. We prepared siloxy vinylallenes by the base-induced equilibrium of silyl-protected allyl-propargyl alcohols. We found that these systems react with imines to form cycloadducts with total regio and facial selectivity, but only moderate endo:exo selectivity. The cycloadducts obtained were transformed into indolizidine derivatives. The reaction was studied computationally using DFT and compared to the reaction of siloxydienes. It was found that the main difference between those systems is the higher nucleophilicity of the siloxydienes compared to the siloxy vinylallenes.

## 1. Introduction

Vinylallenes have been used as dienes in Diels–Alder reactions with homo and heterodienophiles for some time [1,2].

**Scheme 1:**
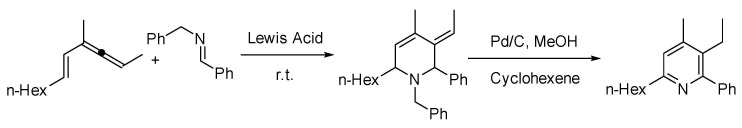
Previous work on alkyl-substituted vinylallenes.

We found that simple vinylallenes have a reactivity similar to monoactivated dienes in the hetero Diels–Alder reaction with aldehydes and imines [3], yielding heterocyclic compounds that can be converted to pyridines when imines were used as heterodienophiles (Scheme 1).

**Scheme 2:**
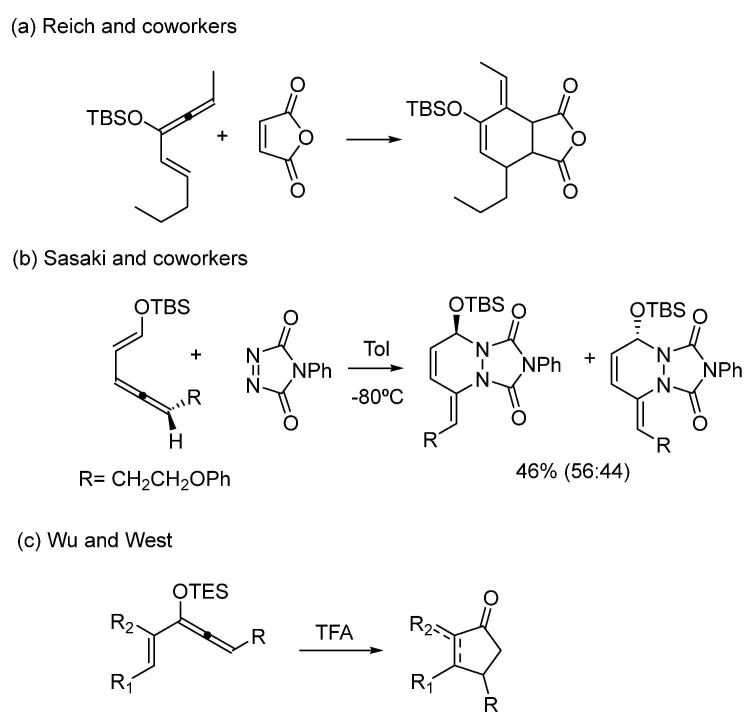
Previous works that use siloxy vinylallenes.

However, the use of more activated vinylallenes in the Diels–Alder reaction has not received much attention, although some examples of their preparation and use exist. Reich and coworkers prepared some siloxy vinylallenes and demonstrated their use as dienes in Diels–Alder reactions with homo dienophiles [4,5] (Scheme 2a).

The use of activated vinylallenes as dienes with heterodienophiles has been even less studied. The most used activating groups are silyl ethers, usually TBS, since they are strong activating groups, are easy to introduce and remove, and are stable under the reaction conditions. Sasaki and coworkers published the reaction of siloxy vinylallenes activated at the vinylic part of the vinylallene with PTAD [6,7]. An example is shown in Scheme 2b.

Siloxy vinylallenes activated on the allenic part of the molecule have also been used by Wu and West, but only for the study of the Nazarov reaction [8] (Scheme 2c).

**Scheme 3:**
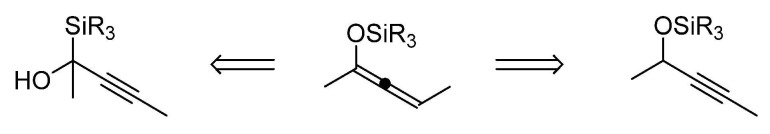
Most used reactions for the preparation of siloxyallenes.

The siloxyallene moiety has been prepared using different approaches, the two most used being the Brook rearrangement of α-hydroxypropargylsilanes [4,9] and the base-induced isomerization of silylated propargyl alcohols [10,11] (Scheme 3).

Those same approaches have been used for the preparation of siloxy vinylallenes. The use of Brook rearrangement in the preparation of siloxy vinylallenes is represented by the work of Reich and co-workers [4,5]. The use of the base-induced isomerization of silylated allyl-propargyl alcohols has been used in the work of Wu and West [8].

Due to our interest in the hetero Diels–Alder using vinylallenes [3], we decided to test if siloxy vinylallenes could react with a heterodienophile in the same way. We decided to use imines, which could lead to substituted piperidine derivatives that could be further transformed.

Thus, the aim of this work is the preparation of siloxy vinylallenes activated in the allenic portion of the molecule, and the study of their reactivity in the hetero Diels–Alder reaction.

## 2. Results and Discussion

### 2.1. Synthesis of the Siloxy Vinylallenes

**Scheme 4:**
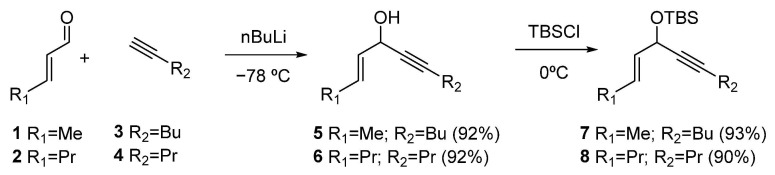
Preparation of the precursors for the siloxy vinylallenes.

**Scheme 5:**
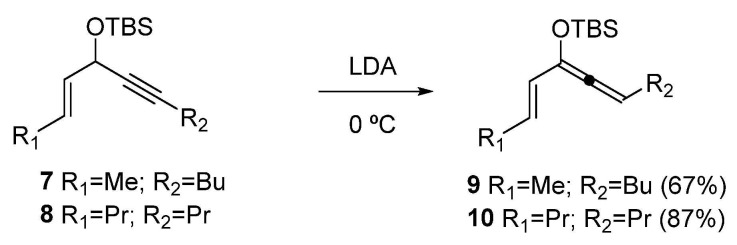
Preparation of the siloxy vinylallenes.

To prepare the required siloxy vinylallenes, we decided to use the base-induced isomerization of silylated propargyl alcohols since it seemed more direct than the preparation of the precursors for the Brook rearrangement. The synthetic procedure is depicted in Scheme 4 and Scheme 5.

Starting from easily accessible unsaturated aldehydes and terminal alkynes, the siloxy vinylallenes could be obtained in three steps.

Two α, β-unsaturated aldehydes, *trans*-crotonaldehyde (**1**) and *trans*-hexen-2-al (**2**), and two alkynes, 1-hexyne (**3**) and 1-pentyne (**4**), were used (Scheme 4). The preparation of the allyl-propargyl alcohols, **5** and **6**, was carried out in high yield from the addition of the anion of alkyne to the aldehyde using nBuLi as base. The protection of the hydroxyl groups with TBSCl was also uneventful and gave **7** and **8** in high yield.

The transformation of the allyl-propargyl silyl ethers into the desired allenes **9** and **10** was carried out following a slight modification of the literature procedures. KOtBu is the most used base for this transformation [8]; however, it gave us some difficulties in the separation step. nBuLi also worked, but some unidentified secondary products were obtained, probably from a nucleophilic attack on the allene. Thus, we decided to try using LDA as the base. Using this base, the reactions occurred cleanly in 20 min at 0 °C (Scheme 5). The siloxy vinylallenes **9** and **10** proved to be unstable to the purification attempts and thus, were used as extracted from the reaction. ^1^H-NMR of the crude reaction extract indicated that they were pure enough to proceed with the cycloaddition step. A small amount of each siloxy vinylallene was purified for identification purposes.

### 2.2. Hetero Diels–Alder Reaction of the Siloxy Vinylallene ***9***

**Scheme 6:**
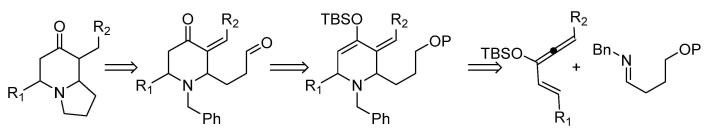
Retrosynthetic analysis of the synthesis of indolizidines from the hetero Diels–Alder reaction of the siloxy vinylallene.

For the hetero Diels–Alder reaction, we envisioned using a linear heterodienophile, which would give a piperidine derivative, but one that could allow further transformation of the cycloadduct into an indolizidine, a structure that we have prepared recently using the hetero Diels–Alder reaction using an activated diene [12]. Indolizidines are a large family of natural heterocyclic compounds, some exhibiting interesting biological activities. The indolizidines we intended to prepare were of the same family as those prepared in our previous paper, the 5,8-disubstituted indolizidines, the largest group of those isolated from the skin of amphibians [13]. The proposed transformation was based on the intramolecular reductive amination (Scheme 6), a route already used for the preparation of the indolizidine skeleton [14,15].

**Scheme 7:**
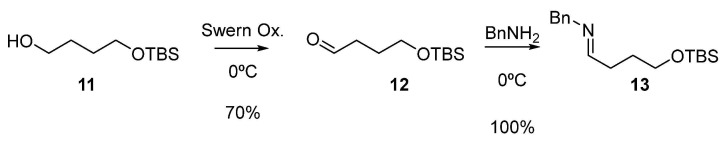
Synthesis of the heterodienophile **13**.

Thus, we prepared the known imine **13** [16], as depicted in Scheme 7.

**Scheme 8:**
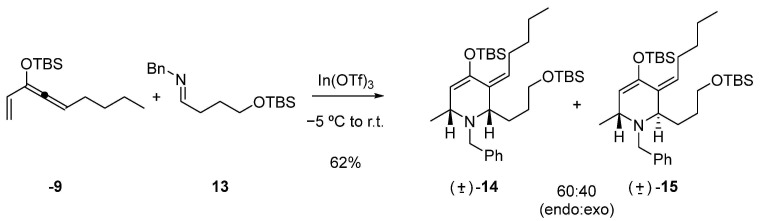
Hetero Diels–Alder reaction of **9** with imine **13**.

The reaction between the siloxy vinylallene **9** and the imine **13** was carried out first using BF_3_·OEt_2_, since this Lewis acid works well in the reaction of alkyl-substituted vinylallenes [3]. However, the yield was low due in part to decomposition of the siloxy vinylallene, in a similar way to what occurred with siloxydienes [12]. Thus, we tried In(OTf)_3_ as the Lewis acid and found that the reaction, carried out in CH_3_CN, was complete overnight (Scheme 8).

After the purification step using flash chromatography, two major compounds were obtained in a 60:40 ratio and 62% combined yield.

The structure of the compounds obtained was studied using 1D and 2D NMR spectroscopy (see Supplementary Materials), and it was deduced that the structures were consistent with the expected cycloadducts (±)-**14** coming from the endo approach and (±)-**15** from the exo approach (Scheme 8).

The relative stereochemistry was difficult to establish with total confidence, since, as happened with the alkyl-substituted vinylallenes [17], the nitrogenated cycloadducts of this type do not give good NOE correlations. Following that experience, we tentatively assigned the relative stereochemistry shown in Scheme 8 for each isomer.

In any case, this result indicates that the siloxy vinylallene reacted as a diene in a hetero Diels–Alder reaction with total regio and facial selectivity, but only moderate endo:exo selectivity.

**Scheme 9:**
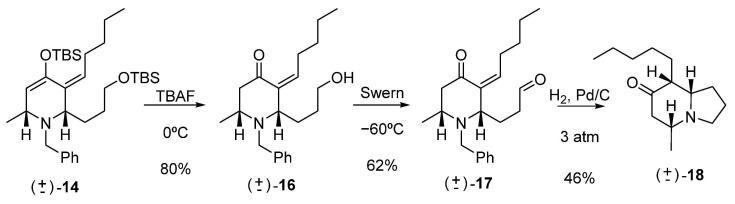
Transformation of the cycloadduct into the indolizidine **18**.

The major isomer was then treated with TBAF to remove both TBS groups (Scheme 9). The reaction was conducted in THF at 0 °C for 12 h, yielding **16** in 80% yield. Compound **16** was then oxidized to an aldehyde using a Swern oxidation. The aldehyde **17** proved to be quite unstable, and thus, it was hydrogenated at room temperature and 3 atm of pressure, using 10% Pd/C as the catalyst. After 12 h, the indolizidine **18** was obtained in 46% yield (Scheme 9). In this reaction, the benzyl group on the nitrogen was removed, and the iminium formed between the aldehyde and the nitrogen was reduced. The α, β-unsaturated ketone was also reduced under the conditions employed.

**Figure 1:**
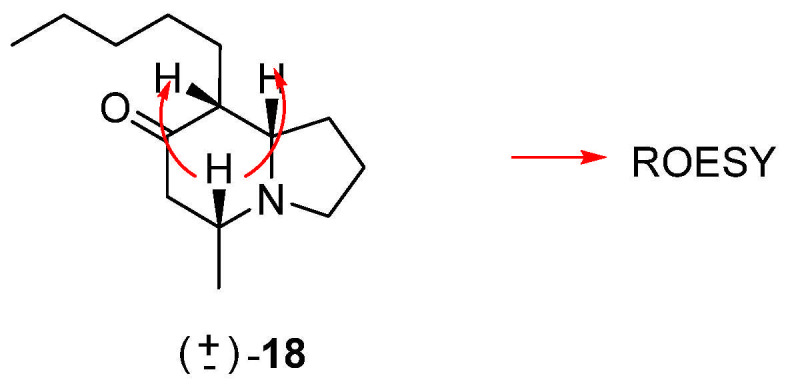
ROESY correlation found for **18**.

The structures were confirmed using 1D and 2D NMR spectroscopy. The relative stereochemistry was established on **18** based on a ROESY spectrum of the compound, since on **16** and **17** we found the same difficulties as for **14**. Figure 1 shows the relevant ROESY correlations found.

This confirms the relative stereochemistry postulated for the cycloadducts in the hetero Diels–Alder reaction.

Since we have recently prepared a compound with the indolizidine skeleton using a simple approach in which Δ^1^-pyrroline was used as a heterodienophile [12], and we have found that the siloxy vinylallenes react similarly to the siloxydienes, we decided to test if we could prepare that skeleton in one step. In that case, the extra double bond would allow for further transformation of the structure.

The Δ^1^-pyrroline was prepared from the diethylacetal of 4-aminobutyraldehyde as described in the literature [18] and freshly distilled before each use.

**Scheme 10:**
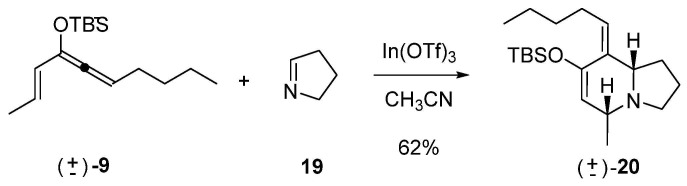
Hetero Diels–Alder of **9** and Δ^1^-pyrroline **19**.

The siloxy vinylallene **9** was thus reacted at −5 °C with Δ^1^-pyrroline (**19**) in CH_3_CN using In(OTf)_3_ as catalyst (Scheme 10). After allowing the reaction to proceed overnight, the crude reaction extract was purified by flash chromatography, and only one compound (**20**) was obtained in a 62% yield. The structure of **20** was studied using NMR spectroscopy, and it was established that it was one of the expected cycloadducts. After further checking the reaction crude mixture, it appears that the other minor isomer was present but is significantly more polar and was mixed with residues from the reaction, making its isolation impossible. The relative stereochemistry of **20** was difficult to establish using ROESY experiments, and thus, we decided to transform **20** into **18** to correlate the stereochemistry.

**Scheme 11:**
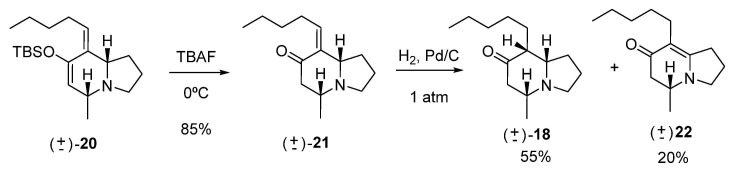
Synthesis of **18** from **20**.

For this transformation, **20** was treated with TBAF at 0 °C, giving, after 12 h, the α, β-unsaturated ketone **21** in an 85% yield (Scheme 11). This ketone was then hydrogenated at atmospheric pressure using 10% Pd/C.

After 12 h, a major compound was obtained in 55% yield, which resulted in an identical product to **18**, together with another compound, which was identified as **22**, isolated in a 20% yield, resulting from the isomerization of the double bond.

The NMR spectra of compound 18 are similar to those obtained for the keto indolizidine prepared in our previous paper [12]. Its preparation demonstrates that following either route, from siloxydienes or from siloxy vinylallenes, similar indolizidine skeletons can be obtained.

From these experiments, it is clear that the major compound in the hetero Diels–Alder reaction is the one coming from the endo approach of the dienophile to the dienic part of the vinylallene.

**Scheme 12:**
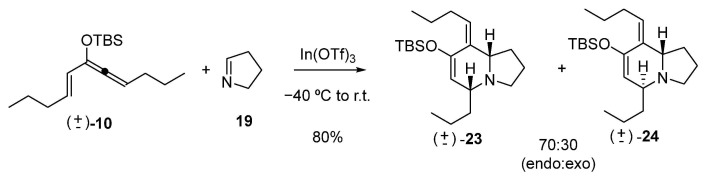
Reaction of siloxy vinylallene 10 and Δ^1^-pyrroline **19**.

After the successful preparation of the indolizidine skeleton from a siloxy vinylallene, we decided to prepare another siloxy vinylallene with a different substitution pattern, in an attempt to change the polarity of the compounds and thus be able to isolate both the endo and exo cycloadducts. Therefore, we prepared siloxy vinylallene **10** and repeated the reaction with Δ^1^-pyrroline. The reaction was carried out under the same conditions as before, and in this case, the two cycloadduct isomers **23** and **24** were isolated in an 80% combined yield (Scheme 12).

The structure and relative stereochemistry of the cycloadducts were studied using mono and bidimensional NMR spectroscopy. Again, we found that the reaction proceeded with total regio and facial selectivity, but the endo:exo ratio was moderate.

**Figure 2:**
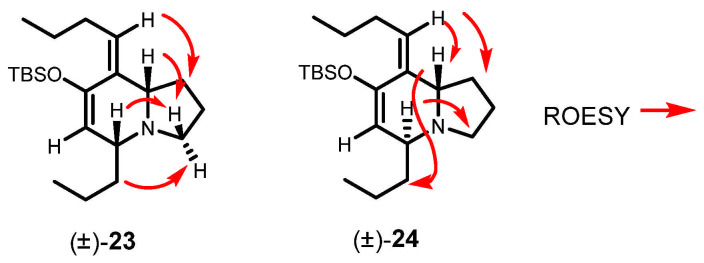
Relevant correlations found in the ROESY experiments.

The correlations found in the ROESY experiment (Figure 2) indicate that the major compound was the one coming from the endo approach of the dienophile. However, as in the previous cases, no direct correlation between the two protons on the stereogenic centers could be observed.

### 2.3. Removal of the TBS Group

**Scheme 13:**
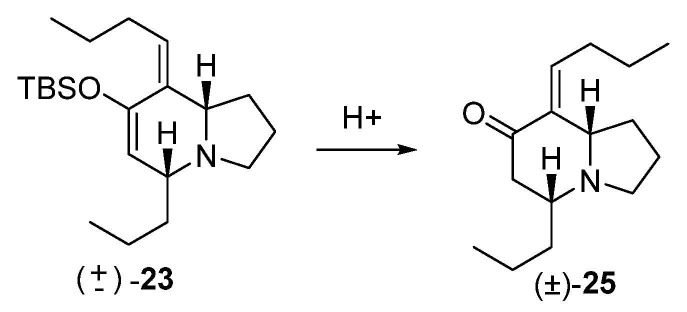
Acid treatment of the endo cycloadduct.

To remove the siloxy group to stabilize the compound, we treated the major isomer (±)-**23** with HCl in THF and, after purification of the crude product, a new compound **25** was isolated in a 46% yield (Scheme 13).

The spectroscopic data indicated that the TBS group has been removed, and an α, β-unsaturated ketone was obtained. However, after analysis of the ROESY spectrum, it was observed that the double bond has changed its stereochemistry, as shown in Scheme 13.

The position of the vinylic proton in the ^1^H-NMR spectrum (7.01 ppm in CDCl_3_, 6.82 ppm in C_6_D_6_) is consistent with a syn disposition relative to the carbonyl group.

**Scheme 14:**
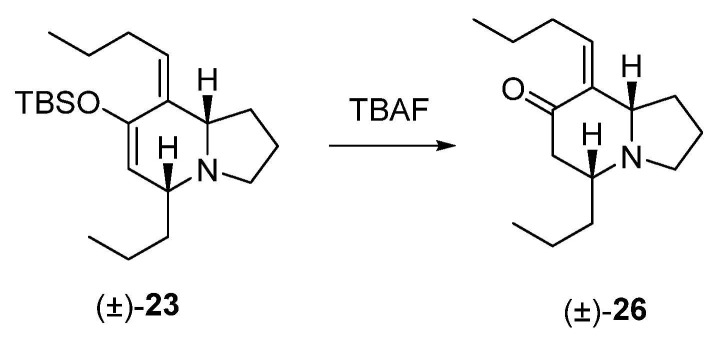
Removal of the TBS group using TBAF.

To confirm that the isomerization was caused by the acid treatment, the TBS group was then removed using TBAF in a neutral medium. This way, compound **26** was obtained in high yield. This compound was similar to **25**, but now the stereochemistry of the double bond was unchanged, and the position of the vinylic proton was consistent with an anti-disposition relative to the carbonyl group (5.72 ppm in CDCl_3_) as shown in Scheme 14.

**Figure 3:**
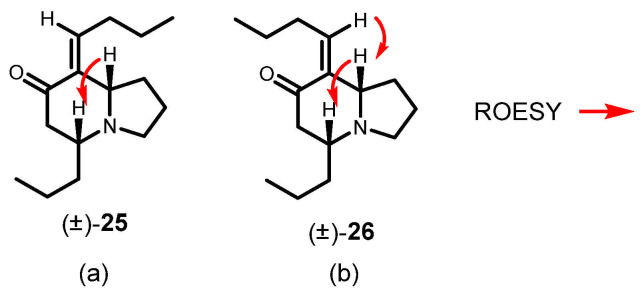
Relevant correlation found in the ROESY experiments for the ketones obtained in the acid (**a**) and fluoride (**b**) removal of the TBS group.

This indicates that the acid deprotection occurs with the intermediation of a cation that undergoes equilibrium, yielding the most stable isomer. The relative stereochemistry of the stereogenic centers was not affected in any of the procedures, as shown in Figure 3 for the ROESY interactions.

These results confirm that the major compound in this hetero Diels–Alder reaction is also the one coming from the endo approach.

Thus, we have demonstrated that siloxy vinylallenes react with heterodienophiles under Lewis acid activation to give heterocyclic cycloadducts in good yield, total facial selectivity, and moderate endo:exo selectivity. The cycloadducts can be easily transformed into indolizidines.

**Scheme 15:**
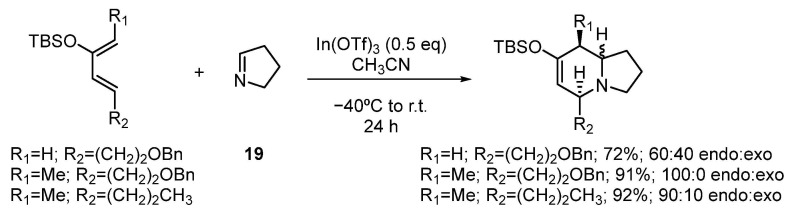
Results of the Hetero Diels–Alder reaction of substituted siloxydienes and Δ^1^-pyrroline [12].

It is interesting to observe that in the related reaction using siloxydienes [12], the endo:exo selectivity was very high when the diene was substituted at both ends, but was low when the R_1_ group was hydrogen (Scheme 15). With the siloxy vinylallene, the selectivity was only 70:30, similar to the less substituted diene. This indicated some differences in the approaches of the diene and the vinylallene to the dienophile. To gain some insight, we decided to study this aspect of the results from a computational point of view.

### 2.4. Computational Results

**Figure 4:**
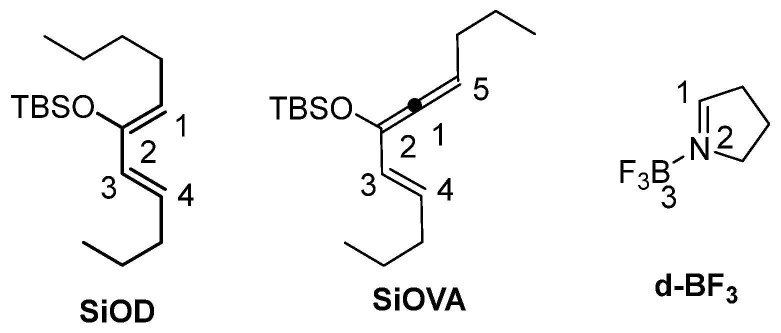
Model compounds used in computational studies and the numbering scheme used in the discussion.

Since we could isolate both cycloadducts from the reaction of siloxy vinylallene **10**, we decided to use it as the model for the computational exploration. For comparison purposes, and since the substitution in the diene seems to be relevant to the endo:exo selectivity of the reaction, we decided to add the calculations of the siloxydiene using a model with the same number of carbons as the siloxy vinylallene (Figure 4).

We studied the relevant points on the reaction coordinate, starting compounds SiOD, SiOVA, d-BF_3_, transition states for the endo (TSN) and exo (TSX) approaches, and final products for the endo (FPN) and exo (FPX) approaches, using DFT theory as implemented in the ORCA program [19]. The functional chosen was B3LYP [20,21] with the D4 correction [22], and def2-SVP was selected as the basis set. As the Lewis acid, we decided to use BF_3_, since calculations for Indium require larger basis sets, and we have found that the reaction also worked with BF_3_.

The starting compounds and the final ones were optimized using the default model implemented in ORCA. For the structure of the transition states, the NEB procedure on ORCA was followed. All stationary points were confirmed by frequency calculation, finding no imaginary frequencies for the initial and final compounds and only one imaginary frequency for the transition states. Those imaginary frequencies were analyzed using IRC to confirm that they corresponded to the reaction studied.

A molecular complex, slightly more stable than the sum of the starting compounds, was found for both endo (MCN) and exo (MCX) approaches of the dienophile complexed to the Lewis acid and the siloxydiene or siloxy vinylallene. These complexes result from the Van der Waals attraction between the reactants (see Supplementary Materials for details).

**Figure 5:**
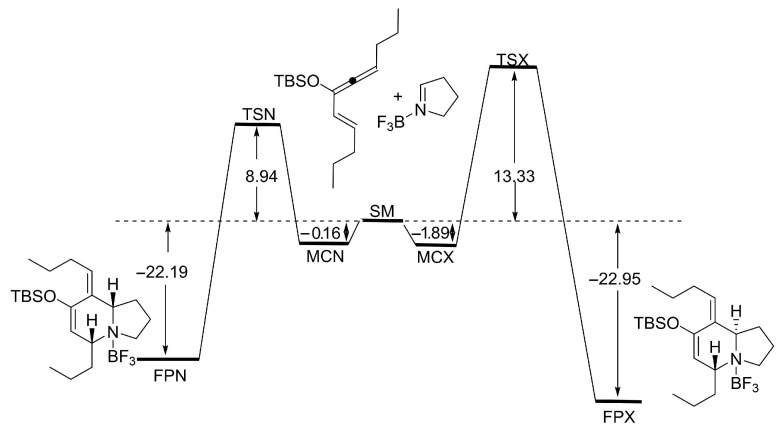
Reaction profile for the reaction of siloxy vinylallene SiOVA and Δ^1^-pyrroline. Gibbs activation energies and reaction energies at 298 K are in kcal/mol.

**Figure 6:**
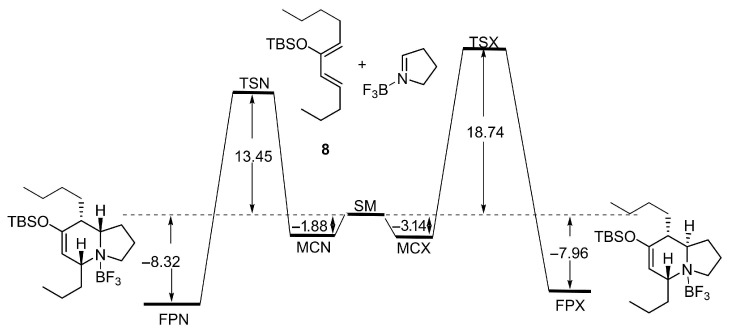
Reaction profile for the reaction of siloxydiene SiOD and Δ^1^-pyrroline. Gibbs activation energies and reaction energies at 298 K are in kcal/mol.

The profile calculated for the reaction of the siloxy vinylallene SiOVA is shown in Figure 5, and the corresponding one for the siloxydiene SiOD in Figure 6.

In both reactions, the activation energy found for the endo approach is smaller than that of the exo approach, confirming that the major component of the reaction mixture must be the isomer coming from the endo approach. Thermodynamically, the exo isomer was more stable than the endo one for the siloxy vinylallene and the opposite for the siloxydiene, although in both cases with a small difference. The larger stabilization on the final products for the siloxy vinylallene can be explained by the conjugation of the double bond coming from the distal double bond of the allene in the cycloadducts.

The activation energy found for the transition states in the siloxy vinylallene is smaller than the one found for the siloxydiene, 8.94 kcal/mol in the endo approach in the siloxy vinylallene vs. 13.45 kcal/mol for siloxydiene, and 13.33 kcal/mol in the exo approach for the siloxy vinylallenes vs. 18.74 kcal/mol for the exo approach for the siloxydiene. A kinetically more favorable reaction is thus expected.

However, the difference between the endo and exo transition states in each reaction is similar, and a similar ratio of cycloadducts should be expected. Thus, this data alone does not explain the difference observed experimentally.

**Figure 7:**
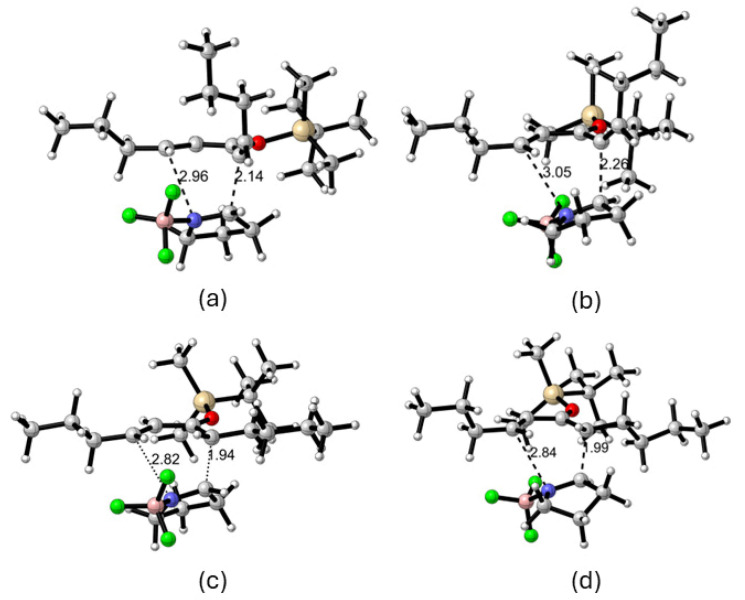
Transition state structures and distances between the reacting atoms. (**a**) Siloxy vinylallene endo approach. (**b**) Siloxy vinylallene exo approach. (**c**) Siloxydiene endo approach. (**d**) Siloxydiene exo approach. Distances are in Å. Graphics represented using Cylview [24].

The study of the transition state geometry indicated that in both cases the reaction is asynchronous, with one bond more formed than the other. In the case of the siloxy vinylallene, those distances are larger than in the case of the siloxydienes (Figure 7), and thus, a less compact transition state could be responsible for the lack of selectivity in this reaction. The differences found for distances between the bond-forming atoms in the endo and exo approaches of the diene (0.88 Å and 0.85 Å) are larger than those found for the vinylallene (0.82 Å and 0.79 Å), indicating a more asynchronous reaction in the former case. Both reactions can be considered highly asynchronous due to the large difference between the forming bonds (>0.5 Å) [23].

Both reactions are of normal electron demand following the FMO formalism, since the most favorable interaction is from the HOMO orbital in the vinylallene (or the diene) and the LUMO orbital in the dienophile (Table 1).

To shed more light on this process, an analysis using conceptual density function theory (CDFT) was undertaken. The study was carried out using the CDFT module in multiwfn [25,26,27] using the same computational level used in the previous calculations.

Table 2 shows the chemical potential (µ), the chemical hardness (η), the global electrophilicity (ω), and the global nucleophilicity (*N*) for the molecules studied.

The chemical potential for the siloxydiene is slightly larger than that of the siloxy vinylallene, and so is expected to be more reactive. The chemical hardness is similar for both compounds acting as dienes. The lower value of µ for the dienophile implies that the charge transfer should be from the diene or vinylallene to the dienophile, as found before. The global electrophilicity and nucleophilicity indicate that the siloxydiene is less electrophilic and more nucleophilic than the siloxy vinylallene. Both the siloxy vinylallene and siloxydiene are moderate electrophiles and strong nucleophiles [28]. The Δ^1^-pyrroline is a strong electrophile and a marginal nucleophile. Thus, a polar reaction is expected.

To study the polarity of the reaction, the Global Electron Density Transfer (GEDT) [29] in the transition states was calculated using the NBO methodology [30]. The results are shown in Table 3.

The data for the endo and exo approaches in the transition state of the reactions confirms that the transfer is from the dienic part of the reaction to the dienophile.

The transfer for both transition states of the approach of the siloxydiene to the dienophile is larger than that of the siloxy vinylallene, indicating that the reaction of the diene is a more polar hetero Diels–Alder reaction.

The condensed charges, Fukui functions, and dual descriptors for the relevant atoms are presented in Table 4. The numbering scheme for Table 4 and Table 5 is shown in Figure 4.

As seen in Table 4, the condensed-to-atom indexes for carbon 1 in the vinylallene or the diene, that is, the one forming the most advanced bond in the transition state, have the largest charge, the largest Fukui function related to electrophilic attack, and the most negative dual descriptor. This indicates that this carbon initiates the bond formation in both the vinylallene and the diene in a two-stage mechanism with a nucleophilic attack onto the imine carbon and then, after that bond has progressed, the other bond is formed. Those quantities are also larger for the diene.

The condensed electrophilicity and nucleophilicity indexes are collected in Table 5. Those results also indicate that the bond formation between C1 of the dienic part of the vinylallene or the diene and C1 of the dienophile should be the most advanced, resulting in an asynchronous transition state.

Those results can explain the shorter distance between the reactants in the transition state for the siloxydiene. This results in a more compact transition state and more differentiation between the endo and exo approaches to the dienophile.

The substitution pattern is important [12]; when the diene is unsubstituted at C1, experimentally, the endo:exo selectivity is low. With an alkyl group, the selectivity is high. In the siloxy vinylallene, the sp carbon has less steric impact. Thus, it seems that the steric factors are important for the selectivity in these reactions. The higher nucleophilicity of the dienes results in a transition state with shorter distances between the reactants, thus making the steric factors more relevant when compared to the siloxy vinylallene.

## 3. Materials and Methods

### 3.1. General Experimental Procedures

All moisture-sensitive reactions were carried out under an argon or nitrogen atmosphere with dry solvents under anhydrous conditions. All solvents and reagents were purified using standard techniques or used as supplied by commercial sources. Reactions under standard conditions were monitored by thin-layer chromatography (TLC) on silica gel 60 F254 plates. Visualization was accomplished with UV light, stained with an ethanolic solution of phosphomolybdic acid or ninhydrin, and developed by heating. Silica gel (200–300 mesh) was used for column chromatography. NMR and spectra were recorded in CDCl_3_ or C_6_D_6_ at 500 MHz for ^1^H NMR and 125 MHz for ^13^C NMR on a Bruker Avance instrument. Chemical shifts are given in (δ) parts per million and coupling constants (J) in Hz. ^1^H- and ^13^C-spectra were referenced using the solvent signal as an internal standard. The data are reported as s = singlet, d = doublet, t = triplet, q = quartet, quintet = quintet, sext = sextet, m = multiplet, dd = doublet of doublets, dt = doublet of triplets, and bs = broad singlet. High-resolution mass spectral analysis (HRMS) data were obtained using a Fison VG AutoSpec spectrometer, Ipswich, UK, via electron impact (EI). The ^1^H NMR and ^13^C NMR spectra and 2D NMR spectroscopy of new compounds are provided in the Supplementary Materials.

### 3.2. Compound Synthesis

Synthesis of *rac-(E)-dec-2-en-5-yn-4-ol* (**5**)

To a solution of 1-hexyne (**3**), (1 g, 12.17 mmol) in THF (60 mL) at −78 °C under argon atmosphere was added nBuLi (1.6 M in hexanes, 14.6 mmol) dropwise. The reaction was allowed to reach −30 °C, and after 40 min, it was cooled again to −78 °C, and (*E*)-but-2-enal (**1**) (0.895 g, 12.78 mmol) was added dropwise. After 3h at −78 °C, the reaction was quenched by the addition of 60 mL of a cold saturated solution of NH_4_Cl and was extracted with 3 × 50 mL of EtOAc, dried over anhydrous Na_2_SO_4_, filtered, and concentrated. The crude extract was purified by flash chromatography (90:10, hexane: EtOAc), affording **5** (1.70 g, 92%).

Colorless oil. ^1^H NMR (CDCl_3_, 500 MHz) δ 5.86 (dq, *J* = 6.4, 15.8 Hz, 1H), 5.63 (dd, *J* = 6.2, 15.2 Hz, 1H), 4.79 (d, *J* = 6.1 Hz, 1H), 2.22 (t, *J* = 6.9 2H), 1.83 (bs, 1H), 1.72 (d, *J* = 6.5 Hz, 3H), 1.54–1.33 (m, 4H), 0.90 (t, *J* = 7.2 Hz, 3H); ^13^C NMR (CDCl_3_, 125 MHz) δ 130.6, 128.1, 86.5, 79.3, 62.9, 30.4, 21.7, 18.2, 17.2, 13.3.

Synthesis of *rac-(E)-undec-4-en-7-yn-6-ol* (**6**)

To a solution of 1-pentyne **4** (817 mg, 12 mmol) in THF (60 mL) at −78 °C was added nBuLi (1.6 M in hexanes, 13.2 mmol) dropwise. The reaction was allowed to reach −30 °C, and after 40 min, it was cooled again to −78 °C, and trans-2-hexenal (**2**) (1.41 g, 14.4 mmol) was added dropwise. After 3 h at −78 °C, the reaction was quenched by the addition of 60 mL of a cold saturated solution of NH_4_Cl and was extracted with 3 × 50 mL of EtOAc, dried over anhydrous Na_2_SO_4_, filtered, and concentrated. The crude extract was purified by flash chromatography (90:10, hexane: EtOAc), affording **6** (1.7 g, 92%).

Colorless oil. ^1^H NMR (CDCl_3_, 500 MHz) δ 0.11 (s, 6H), 0.93 (t, *J* = 7.4 Hz, 3H), 1.00 (t, *J* = 7.2 Hz, 3H), 1.45 (sext, *J* = 7.4 Hz, 2H), 1.57 (sext, *J* = 7.2 Hz, 2H), 2.06 (t, *J* = 7.1 Hz, 2H), 2.34 (t, *J* = 7.2 Hz, 2H), 4.84 (d, *J* = 5.4 Hz, 1H), 5.62 (dd, *J* = 6.2, 15.7 Hz, 1H), 5.88 (dt, *J* = 6.8, 15.7 Hz, 1H); ^13^C NMR (CDCl_3_, 125 MHz) δ 13.5, 13.7, 20.8, 22.1, 34.0, 63.3, 79.8, 86.7, 129.7, 133.4; HRMS (EI^+^): Calcd for C_11_H_18_O [M^+^] 166.1358, found 166.1357.

Synthesis of *rac-(E)-tert-butyl(dec-2-en-5-yn-4-yloxy)dimethylsilane* (**7**)

To a solution of alcohol **5** (2 g, 13.15 mmol) and imidazole (2.7 g, 39.5 mmol) in DCM (30 mL), TBSCl (2.21 g, 32.5 mmol) in 23 mL of DCM was added. The mixture was stirred at room temperature overnight and was quenched by the addition of water (50 mL) and extracted using DCM (3 × 50 mL). The organic extracts were washed with brine, dried over Na_2_SO_4_, filtered, and concentrated. The crude extract was purified by flash chromatography (99:1 hexane: EtOAc), affording **7** (3.25 g, 93%).

Colorless oil. ^1^H NMR (CDCl_3_, 500 MHz) δ 5.80 (dq, *J* = 6.5, 14.4 Hz, 1H), 5.56 (dd, *J* = 5.5, 15.1 Hz, 1H), 4.85 (d, *J* = 5.2 Hz), 2.24 (t, *J* = 6.9 Hz, 2H), 1.72 (d, *J* = 6.6 Hz, 3H), 1.55–1.40 (m, 4H), 0.93 (s, 12H), 0.92 (t, *J* = 4.0, 3H), 0.15 (s, 6H); ^13^C NMR (CDCl_3_, 125 MHz) δ 131.6, 126.1, 85.7, 80.3, 63.6, 30.7, 25.8, 21.9, 18.4, 18.3, 17.4, 13.5, −4.7.

Synthesis of *rac-(E)-tert-butyldimethyl(undec-4-en-7-yn-6-yloxy)silane* (**8**)

To a solution of alcohol **6** (1.8 g, 10.8 mmol) in DCM (50 mL), imidazole (2.21 g, 32.5 mmol) and TBSCl (2.21 g, 32.5 mmol) were added, and the mixture was stirred at room temperature overnight. The reaction was quenched by the addition of water (25 mL) and was extracted using DCM (3 × 50 mL). The organic extracts were washed with brine, dried over Na_2_SO_4_, filtered, and concentrated. The crude extract was purified by flash chromatography (100% hexane), affording **8** (2.76 g, 90%).

Colorless oil. ^1^H NMR (CDCl_3_, 500 MHz) δ 0.13 (s, 3H), 0.14 (s, 3H), 0.91 (t, *J* = 7.3 Hz, 3H), 0.92 (s, 9H), 0.98 (t, *J* = 7.4 Hz, 3H), 1.42 (sext, *J* = 7.4 Hz, 2H), 1.54 (sext, *J* = 7.3 Hz, 2H), 2.03 (q, *J* = 7.2 Hz, 2H), 2.20 (dt, *J* = 7.1, 2.0 Hz, 2H), 4.86 (d, *J* = 5.1 Hz, 1H), 5.52 (ddt, *J* = 15.3, 5.6, 1.3 Hz, 1H), 5.77 (dtd, *J* = 15.3, 6.7, 1.3 Hz, 1H); ^13^C NMR (CDCl_3_, 125 MHz) δ −4.7, −4.5, 13.4, 13.6, 18.3, 20.8, 22.0, 22.2, 25.8, 33.9, 63.8, 80.6, 85.4, 130.6, 131.1; HRMS (EI^+^): Calcd for C_17_H_32_OSi [M^+^] 280.2222, found 280.2234.

Synthesis of *rac-(E)-tert-butyl(deca-2,4,5-trien-4-yloxy)dimethylsilane* (**9**)

To a solution of **7** (200 mg, 0.75 mmol) in THF (4 mL) at 0 °C was added cold (−10 °C) LDA (1.58 mL, 0.5 M in THF, 0.79 mmol). After stirring for 20 min, an aqueous solution of NH_4_Cl (5 mL) was added. The reaction mixture was extracted with ethyl ether (3 × 10 mL). The combined organic extracts were washed with brine, dried over anhydrous Na_2_SO_4_, and concentrated. The oily residue obtained (134 mg, 0.50 mmol) resulted in almost pure **9** by NMR analysis (67%).

Colorless oil. ^1^H NMR (C_6_D_6_, 500 MHz) δ 6.11 (dq, *J* = 6.6, 14.5 Hz, 1H), 5.94 (d, *J* = 15.2 Hz, 1H), 5.56 (t, *J* = 6.4 Hz, 1H), 1.96–1.90 (m, 2H), 1.59 (d, *J* = 6.6 Hz, 3H), 1.33–1.17 (m, 4H), 0.99 (s, 9H), 0.78 (t, *J* = 7.3 Hz, 3H), 0.17 (s, 6H); ^13^C NMR (C_6_D_6_, 125 MHz) δ 198.9, 127.6, 126.1, 124.1, 101.8, 30.9, 25.8, 22.3, 18.2, 17.6, 13.8, −4.6; HRMS (EI^+^): Calcd for C_16_H_30_OSi [M^+^] 266.2066, found 266.2079.

Synthesis of *rac-(E)-tert-butyldimethyl(undeca-4,5,7-trien-6-yloxy)silane* (**10**)

To a solution of **8** (1 g, 3.6 mmol) in THF (36 mL) at −10 °C was added cold (−10 °C) LDA (0.5 M in THF, 3.93 mmol). After stirring for 30 min, imidazole (267 mg, 3.93 mmol) in THF (3 mL) was added. The reaction mixture was poured over hexane, and the solid was filtered out. The resulting solution was concentrated, and the oily residue obtained (870 mg) resulted in an almost pure **10** by NMR analysis (87% yield).

Colorless oil. ^1^H NMR (C_6_D_6_, 500 MHz) δ 0.23 (s, 3H), 0.24 (s, 3H), 0.84 (t, *J* = 7.4 Hz, 3H), 0.86 (t, *J* = 7.4 Hz, 3H), 1.06 (s, 9H), 1.29–1.44 (m, 4H), 1.95 (m, 2H), 2.04 (q, *J* = 7.4 Hz, 2H), 5.61 (t, *J* = 6.6, 1H), 6.03 (d, *J* = 15.4 Hz, 1H), 6.22 (dt, *J* = 15.4, 7.2 Hz, 1H); ^13^C NMR (C_6_D6, 125 MHz) δ −4.6, −4.4, 13.8, 13.9, 18.5, 22.3, 23.0, 26.0, 101.8, 126.2, 126.8, 129.8, 199.6; HRMS (EI^+^): Calcd for C_17_H_32_OSi [M^+^] 280.2222, found 280.2229.

Synthesis of *4-((tert-butyldimethylsilyl)oxy)butanal* (**12**)

To a solution of DMSO (0.84 mL, 11.76 mmol) in DCM (26 mL) at −60 °C under argon atmosphere, were added 2.94 mL of oxalyl chloride (2 M in DCM, 5.88 mmol). The mixture was stirred for 20 min, and then a solution of **11** (1.0 g, 4.9 mmol) in DCM (7 mL) was added. After stirring for 1 h at −60 °C, 3.4 mL of TEA (24.5 mmol) was added, and the reaction was allowed to reach room temperature. Then, water was added, and the reaction was extracted with DCM (3 × 50 mL). The organic phase was washed with 1% aqueous HCl (40 mL), water (40 mL), saturated solution of NaHCO_3_, brine (40 mL), dried over anhydrous Na_2_SO_4_, filtered, and concentrated. After purification using flash chromatography (90:10 hexane: EtOAc), **12** was obtained (0.7 g, 70%).

Colorless oil. ^1^H NMR (C_6_D_6_, 500 MHz) 9.66 (t, *J* = 1.6 Hz, 1H), 3.53 (d, *J* = 5.9 Hz, 2H), 2.38 (dt, *J* = 1.6, 7.0 Hz, 2H), 1.74 (m, 2H), 0.76 (s, 9H), −0.07 (s, 6H); ^13^C NMR (C_6_D_6_, 125 MHz) δ 202.5, 62.0, 40.7, 25.8, 25.5, 18.2, −5.5.

Synthesis of *(E)-N-benzyl-4-((tert-butyldimethylsilyl)oxy)butan-1-imine* (**13**).

To a mixture of **12** (0.5 g, 2.46 mmol) and MgSO_4_ (12 mg) in DCM (3 mL) at 0 °C, 0.27 mL of BnNH_2_ (0.263 g, 2.46 mmol) was added dropwise. The mixture was stirred for 30 min at 0 °C, then filtered and the solvent removed under reduced pressure, yielding imine **13** (0.717 g, 2.46 mmol, 100%).

Colorless oil. ^1^H NMR (C_6_D_6_, 500 MHz) δ 7.45 (s, 1H), 7.30 (d, *J* = 7.44 Hz, 2H), 7.21 (d, *J* = 7.6 Hz, 2H), 7.12–7.09 (m, 1H), 4.42 (s, 2H), 3.52 (t, *J* = 6.1 Hz, 2H), 2.23 (bs, 2H), 1.75 (q, *J* = 6.9 Hz, 2H), 0.97 (s, 9H), 0.03 (s, 6H); ^13^C NMR (C_6_D_6_, 125 MHz) δ 164.1, 140.3, 128.3, 126.7, 65.1, 62.4, 32.3, 28.9, 25.9, 18.2, −5.3.

*Hetero Diels–Alder reaction of* **9** *and* **13** *with In(OTf)3*

To a solution of In(OTf)_3_ (328 mg, 0.58 mmol) in CH_3_CN (50 mL) at −5 °C, imine **13** (340 mg, 1.17 mmol) dissolved in CH_3_CN (5 mL) was added via cannula. The mixture was stirred for 5 min, then vinylallene **9** (310 mg, 1.17 mmol) dissolved in CH_3_CN (5 mL) was added via cannula. The reaction mixture was stirred overnight. A cold saturated aqueous solution of NaHCO_3_ (40 mL) was added to stop the reaction. Extraction was performed with EtOAc (3 × 40 mL). The combined organic phases were washed with a saturated aqueous solution of NaCl, dried over anhydrous Na_2_SO_4_, and concentrated. The reaction product was purified by column chromatography (95:5, hexane: EtOAc). Compounds **14** (236 mg, 0.43 mmol) and **15** (158 mg, 0.29 mmol) were obtained in a 60:40 ratio and 62% yield.

*rac-(2S,6R,Z)-1-benzyl-4-((tert-butyldimethylsilyl)oxy)-2-(3-((tert-butyldimethylsilyl)oxy)propyl)-6-methyl-3-pentylidene-1,2,3,6-tetrahydropyridine* (**14**)

Colorless oil. IR (ν_max_, cm^−1^): 2956, 2858, 1464, 1253, 1100; ^1^H NMR (C_6_D_6_, 500 MHz) δ 7.47 (d, *J* = 7.5 Hz, 2H), 7.23 (t, *J* = 7.5 Hz, 2H), 7.13 (t, *J* = 7.5 Hz, 1H), 5.10 (t, *J* = 5.0 Hz, 1H), 4.82 (bs, 1H), 3.73 (d, *J* = 14 Hz, 1H), 3.64 (d, *J* = 13.5 Hz, 1H), 3.61–3.55 (m, 2H), 3.26 (bs, 1H), 3.06 (t, *J* = 7.0 Hz, 1H), 2.82–2.78 (m, 1H), 2.53–2.49 (m, 1H), 1.96–1.92 (m, 1H), 1.81–1.78 (m, 1H), 1.70–1.61 (m, 2H), 1.43 (bs, 4H), 1.22 (d, *J* = 7.0 Hz, 3H), 1.04 (s, 9H), 0.99 (s, 9H), 0.96 (t, *J* = 6.5 Hz, 3H), 0.22 (s, 6H), 0.07 (s, 6H); ^13^C NMR (C_6_D_6_, 125 MHz) δ 145.9, 140.8, 130.2, 129.9, 128.8, 128.2, 126.9, 110.5, 67.2, 63.2, 61.6, 54.4, 32.9, 32.5, 30.8, 29.3, 27.1, 25.9, 23.3, 22.7, 18.3, 14.0, −4.1, −5.4.

*rac-(2R,6R,Z)-1-benzyl-4-((tert-butyldimethylsilyl)oxy)-2-(3-((tert-butyldimethylsilyl)oxy)propyl)-6-methyl-3-pentylidene-1,2,3,6-tetrahydropyridine* (**15**)

Colorless oil. IR (ν_max_, cm^−1^): 3392, 2929, 2869, 1693, 1434, 1069; ^1^H NMR (C_6_D_6_, 500 MHz) δ 7.48 (d, *J* = 7.5 Hz, 2H), 7.24 (t, *J* = 7.5 Hz, 2H), 7.11 (t, *J* = 7.5 Hz, 1H), 5.11 (t, *J* = 7.5 Hz, 1H), 4.80 (s, 1H), 3.72 (d, *J* = 14 Hz, 1H), 3.60–3.57 (m, 3H), 3.54 (d, *J* = 14 Hz, 1H), 3.22 (bs, 1H), 2.71–2.59 (m, 2H), 1.88–1.82 (m, 1H), 1.67−1.62 (m, 3H), 1.36 (bs, 4H), 1.12 (d, *J* = 7 Hz, 3H), 1.03 (s, 9H), 1.00 (s, 9H), 0.98–0.92 (m, 3H), 0.21 (s, 6H), 0.07 (s, 6H); ^13^C NMR (C_6_D_6_, 125 MHz) δ 147.8, 141.2, 131.3, 129.2, 128.6, 128.2, 126.7, 111.1, 63.1, 51.4, 50.5, 32.8, 30.2, 29.2, 26.0, 25.1, 22.7, 20.1, 18.3, 14.1.

Synthesis of *rac-(2S,6R,Z)-1-benzyl-2-(3-hydroxypropyl)-6-methyl-3-pentylidenepiperidin-4-one* (**16**)

A small portion of TBAF was added to a solution of the cycloadduct **14** (150 mg, 0.26 mmol) in THF (5 mL) at 0 °C. After 12 h of stirring and after all the starting material had been consumed, cold water (10 mL) was added. Extraction was carried out with ethyl ether (4 × 10 mL). The combined organic phases were washed with a saturated NaCl solution (10 mL) and dried with Na_2_SO_4_. The solvent was removed under reduced pressure, and the reaction product was purified by column chromatography (60:40, hexane: EtOAc), yielding compound **16** (68 mg, 0.21 mmol, 80%).

^1^H NMR (C_6_D_6_, 500 MHz) δ 7.31 (d, *J* = 7.5 Hz, 2H), 7.20 (t, *J* = 7.5 Hz, 2H), 7.11 (t, *J* = 7.5 Hz, 1H), 5.49 (t, *J* = 7.5 Hz, 1H), 3.61 (d, *J* = 14.0 Hz, 1H), 3.40 (d, *J* = 14.0 Hz, 1H), 3.27–3.20 (m, 2H), 3.10 (t, *J* = 7.5 Hz, 1H), 2.70–2.61 (m, 3H), 2.36–2.24 (m, 2H), 1.55–1.48 (m, 1H), 1.42–1.24 (m, 8H), 0.93 (d, *J* = 3.0 Hz, 3H), 0.90 (t, *J* = 7.0 Hz, 3H); ^13^C NMR (C_6_D_6_, 125 MHz) δ 200.2, 141.1, 140.1, 135.8, 129.2, 128.2, 127.1, 66.1, 62.1, 58.0, 53.4, 46.6, 35.1, 31.9, 29.8, 28.7, 22.5, 21.9, 13.9; HRMS (EI^+^): Calcd for C_21_H_31_NO_2_ [M^+^] 329.2355, found 329.2354.

Synthesis of *rac-3-((2S,6R,Z)-1-benzyl-6-methyl-4-oxo-3-pentylidenepiperidin-2-yl)propanal* (**17**)

To a DMSO solution (33 mg, 0.03 mL, 0.426 mmol) in DCM (3 mL) at −60 °C under an argon atmosphere, 0.09 mL of Cl_2_(CO)_2_ (2 M in DCM, 0.18 mmol) was added. The mixture was stirred for 20 min at the same temperature. Then, a solution of **16** (50 mg, 0.15 mmol) in DCM (1 mL) was added via cannula. The mixture was stirred for 1 h at −60 °C, and then 0.10 mL of TEA (75.8 mg, 0.75 mmol) was added, and the reaction was allowed to reach room temperature. After 4 h of stirring, water was added, and the mixture was extracted with DCM (3 × 10 mL). The combined organic phases were washed with 1% HCl (aq) solution (10 mL), water (10 mL), saturated aqueous NaHCO_3_ solution (10 mL), and saturated aqueous NaCl solution (10 mL), then dried over anhydrous Na_2_SO_4_ and concentrated. The resulting crude was purified by column chromatography (80:20, hexane: EtOAc), yielding aldehyde **17** (30 mg, 0.092 mmol, 62%).

^1^H NMR (C_6_D_6_, 500 MHz) δ 9.12 (s, 1H), 7.21–7.09 (m, 5H), 5.46 (t, *J* = 7.6 Hz, 1H), 3.51 (d, *J* = 14.0 Hz, 1H), 3.29 (d, *J* = 14.0 Hz, 1H), 3.00 (t, *J* = 7.6 Hz, 1H), 2.69–2.54 (m, 3H), 2.24–2.22 (m, 2H), 1.61–1.62 (m, 1H), 1.44–1.23 (m, 7H), 0.89 (t, *J* = 7.2 Hz, 3H), 0.86 (d, *J* = 6.4 Hz, 3H); ^13^C NMR (C_6_D_6_, 125 MHz) δ 199.7, 167.0, 142.1, 139.9, 135.1, 129.3, 127.4, 127.1, 64.8, 58.1, 53.2, 46.5, 40.4, 31.8, 30.9, 28.7, 22.5, 21.8, 13.9.

Synthesis of *rac-(5R,8aS)-5-methyl-8-pentylhexahydroindolizin-7(1H)-one* (**18**)

To a solution of **17** (30 mg, 0.092 mmol) in MeOH (2 mL), at room temperature, 10% Pd/C (3 mg) was added and placed in a Parr hydrogenator at 3 atm. After 12 h of reaction, it was filtered, and the solvent was removed under vacuum, obtaining **18** (14 mg, 0.042 mmol, 46%).

^1^H NMR (C_6_D_6_, 500 MHz) δ 2.91 (dt, *J* = 2.0, 8.5 Hz, 1H), 2.14 (d, *J* = 10.0 Hz, 1H), 2.11–2.00 (m, 3H), 1.81–1.72 (m, 2H), 1.67–1.55 (m, 3H), 1.50–1.45 (m, 1H), 1.35–1.16 (m, 8H), 0.84 (t, *J* = 7.0 Hz, 3H), 0.80 (d, *J* = 5.5 Hz, 3H); ^13^C NMR (C_6_D_6_, 125 MHz) δ 208.0, 68.8, 57.1, 55.8, 50.6, 49.2, 32.5, 30.3, 27.7, 26.0, 22.7, 21.5, 21.2, 14.1; HRMS (FAB^+^): Calcd for C_14_H_25_NO [M^+^] 223.1936, found 223.1929.

*Hetero Diels–Alder reaction of* **9** *and Δ^1^-pyrroline* (**19**)

To a solution of In(OTf)_3_ (210 mg, 0.38 mmol) in CH_3_CN (25 mL) at −5 °C, a solution of **19** (52 mg, 0.75 mmol) in CH_3_CN (2 mL) was added via cannula. The mixture was stirred for 5 min, then a solution of **9** (200 mg, 0.75 mmol) in CH_3_CN (2 mL) was added via cannula. The reaction mixture was stirred overnight. A cold, saturated aqueous solution of NaHCO_3_ (30 mL) was added to stop the reaction. The reaction was extracted with EtOAc (3 × 30 mL). The combined organic phases were washed with a saturated aqueous solution of NaCl, dried over anhydrous Na_2_SO_4_, and concentrated. The reaction product was purified by column chromatography (80:20, hexane: EtOAc), yielding 156 mg of **20** (0.47 mmol, 62%).

*rac-(5R,8aS,Z)-7-((tert-butyldimethylsilyl)oxy)-5-methyl-8-pentylidene-1,2,3,5,8,8a-hexahydroindolizine* (**20**)

IR (ν_max_, cm^−1^): 2959, 2859, 2780, 1611, 1463, 1254; ^1^H NMR (C_6_D_6_, 500 MHz) δ 5.29 (t, *J* = 7.0 Hz, 1H), 4.89 (s, 1H), 3.24 (dt, *J* = 2.0, 8.5 Hz, 1H), 2.89–2.85 (m, 2H), 2.78–2.72 (m, 1H), 2.67–2.63 (m, 1H), 1.94 (q, *J* = 8.4 Hz, 1H), 1.82 (t, *J* = 8.5 Hz, 1H), 1.78–1.73 (m, 1H), 1.61–1.55 (m, 1H), 1.44–1.27 (m, 5H), 1.16 (d, *J* = 6.5 Hz, 3H), 1.02 (s, 9H), 0.92 (t, *J* = 6.5 Hz, 3H), 0.21 (s, 6H); ^13^C NMR (C_6_D_6_, 125 MHz) δ 149.4, 133.9, 126.5, 113.8, 65.9, 57.3, 52.6, 33.0, 29.1, 28.9, 26.2, 22.8, 21.7, 21.3, 18.6, 14.1, −4.0.

Synthesis of *(5R,8aS,Z)-5-methyl-8-pentylidenehexahydroindolizin-7(1H)-one* (**21**)

A small portion of TBAF was added to a solution of **20** (70 mg, 0.20 mmol) in THF (2 mL) at 0 °C. The reaction was monitored by TLC. After 12 h of stirring and complete consumption of the starting material, cold water (10 mL) was added. Extraction was carried out with ethyl ether (4 × 10 mL). The combined organic phases were washed with a saturated NaCl solution (10 mL) and dried over Na_2_SO_4_. The solvent was removed under reduced pressure, and the reaction product was purified by column chromatography (20:80, hexane: EtOAc), yielding 37 mg of **21** (0.17 mmol, 85%).

IR (ν_max_, cm^−1^): 2961, 2873, 2787, 1716, 1375, 1187; ^1^H NMR (C_6_D_6_, 500 MHz) δ 5.58 (dt, *J* = 1.5, 7.5 Hz, 1H), 3.01 (dt, *J* = 1.5, 8.0 Hz, 1H), 2.69–2.66 (m, 2H), 2.59 (bs, 1H), 2.39 (dd, *J* = 3.5, 16.0 Hz, 1H), 2.25–2.15 (m, 3H), 1.76–1.59 (m, 4H), 1.48–1.31 (m, 4H), 0.87 (q, *J* = 7.0 Hz, 3H), 0.84 (d, *J* = 6.0 Hz, 3H); ^13^C NMR (C_6_D_6_, 125 MHz) δ 199.3, 138.2, 137.9, 67.4, 55.6, 51.2, 49.7, 31.9, 28.9, 28.8, 22.5, 21.4, 21.1, 13.9; HRMS (FAB^+^): Calcd for C_14_H_23_NO [M^+^] 221.1780, found 221.1768.

*Hydrogenation of* **21**

To a solution of **21** (42 mg, 0.19 mmol) in MeOH (4 mL), 10% Pd/C (6 mg, 0.02 mmol) was added, and the mixture was placed in a hydrogen atmosphere at 1 atm pressure. After 12 h of reaction, the reaction mixture was filtered, and the solvent was removed under reduced pressure. The crude reaction mixture was purified by column chromatography (40:60, hexane: EtOAc), yielding **18** (23.3 mg, 0.11 mmol, 55%) and 8.4 mg of the isomerization compound **22** (0.04 mmol, 20%).

*rac-(R)-5-methyl-8-pentyl-2,3,5,6-tetrahydroindolizin-7(1H)-one* (**22**)

^1^H NMR (C_6_D_6_, 500 MHz) δ 2.92–2.83 (m, 1H), 2.77–2.72 (m, 1H), 2.47–2.32 (m, 3H), 2.23–2.03 (m, 4H), 1.65–1.60 (m, 2H), 1.43–1.40 (m, 4H), 1.30–1.22 (m, 2H), 0.96 (t, *J* = 6.8 Hz, 3H), 0.71 (t, *J* = 6.4 Hz, 3H); ^13^C NMR (C_6_D_6_, 125 MHz) δ 189.0, 163.6, 163.3, 52.3, 50.0, 44.2, 32.1, 30.0, 29.9, 25.9, 23.0, 20.9, 17.5, 14.2.

*Hetero Diels–Alder reaction of* **10** *and Δ^1^-pyrroline* (**19**)

To a solution of In(OTf)_3_ (871 mg, 1.55 mmol) in dry CH_3_CN (15 mL) at 0 °C was added a solution of freshly distilled Δ^1^-pyrroline (**19**) (257 mg, 3.72 mmol) in CH_3_CN (3 mL) at 0 °C under argon. After 15 min, the mixture was cooled at −40 °C, and a solution of siloxy vinylallene **10** (869 mg, 3.10 mmol) in CH_3_CN (5 mL) was added. The reaction mixture was allowed to slowly warm up to room temperature and was stirred for 24 h, quenched with NaHCO_3_ aqueous solution, extracted with EtOAc, washed with brine, dried over Na_2_SO_4_, filtered, and concentrated in vacuo. The residue was purified by flash chromatography to give the corresponding products, **23** and **24** (870 mg, 80%) in a 70:30 ratio.

*rac-(5R,8aS,Z)-7-((tert-butyldimethylsilyl)oxy)-8-butylidene-5-propyl-1,2,3,5,8,8a-hexahydroindolizine* (**23**)

Colorless oil. ^1^H NMR (C_6_D_6_, 500 MHz) δ 0.10 (s, 6H), 0.78 (t, *J* = 7.6 Hz, 3H), 0.84 (t, *J* = 7.4 Hz, 3H), 0.89 (s, 9H), 1.49–1.14 (m, 5H), 1.73–1.55 (m. 3H), 1.82 (q, *J* = 8.7 Hz, 1H), 2.60 (m, 1H), 2.75 (t, *J* = 7.6 Hz, 1H), 2.80 (bs, 1H), 3.10 (td, *J* = 8.7, 2.7 Hz, 1H), 4.94 (s, 1H), 5.16 (t, *J* = 7.4 Hz, 1H); ^13^C NMR (C_6_D_6_, 125 MHz) δ −3.8, −3.7, 14.2, 14.8, 18.2, 18.8, 22.0, 24.1, 26.4, 29.1, 31.7, 37.2, 52.6, 62.1, 66.2, 112.1, 126.2, 134.4, 150.2; HRMS (EI^+^): Calcd for C_21_H_39_NOSi [M^+^] 349.2801, found 349.2794.

*rac-(5R,8aR,Z)-7-((tert-butyldimethylsilyl)oxy)-8-butylidene-5-propyl-1,2,3,5,8,8a-hexahydroindolizine* (**24**)

Colorless oil. ^1^H NMR (C_6_D_6_, 500 MHz) δ 0.32 (s, 3H), 0.34 (s, 3H). 1.05 (t, *J* = 6.7 Hz, 3H), 1.08 (t, *J* = 7.4 Hz, 3H), 1.14 (s, 9H), 1.14–1.49 (m, 4H), 1.95 (m, 2H), 2.00 (m, 2H), 2.77 (q, *J* = 8.2 Hz, 2H), 2.94 (m, 2H), 3.35 (m, 1H), 3.87 (t, *J* = 5.5 Hz, 1H), 5.22 (d, *J* = 4.1 Hz, 1H), 5.39 (t, *J* = 7.4 Hz, 1H); ^13^C NMR (C_6_D_6_, 125 MHz) δ −4.0, −3.9, 14.1, 14.6, 18.7, 20.2, 22.6, 24.0, 26.3, 31.3, 31.9, 37.2, 51.6, 55.8, 60.8, 111.4, 127.31, 132.1, 148.2; HRMS (EI^+^): Calcd for C_21_H_39_NOSi [M^+^] 349.2801, found 349.2794.

*HCl treatment of* **23**

To a solution of **23** (850 mg, 2.4 mmol) in THF (10 mL) were added 4 mL of 10% aq HCl. The reaction was stirred at rt for 3 h. Then a saturated solution of NaHCO_3_ was added, and the reaction was extracted with ether (3 × 50 mL), dried over anhydrous Na_2_SO_4_, filtered, and concentrated. The crude extract was purified by flash chromatography (35:65, hexane: EtOAc), yielding 263 mg of (5R*,8aS*, *E*)-8-butylidene-5-propylhexahydroindolizin-7(1H)-one (**25**) (46% yield).

Colorless oil, ^1^H NMR (C_6_D_6_, 500 MHz) δ 0.77 (t, *J* = 7.3 Hz, 3H), 0.79 (t, *J* = 7.4 Hz, 3H), 1.13, (m, 3H), 1.24 (sext, *J* = 7.4 Hz, 2H), 1.39 (m, 3H), 1.45–1.62 (m, 2H), 1.85 (dq, *J* = 7.5, 1.3 Hz, 2H), 1.98 (m, 2H), 2.03 (quint, *J* = 7.8 Hz, 2H), 2.31 (m, 2H), 2.51 (m, 1H), 2.75 (dt, *J* = 7.8, 4.3 Hz, 1H), 3.28 (t, *J* = 7.6 Hz, 1H), 6.82 (dt, *J* = 7.6, 2.5 Hz, 1H); ^13^C NMR (C_6_D_6_, 125 MHz) δ 14.0, 14.4, 18.7, 22.4, 22.8, 30.9, 31.8, 37.3, 42.8, 47.5, 56.3, 63.5, 139.0, 140.0, 197.8; HRMS (EI^+^): Calcd for C_15_H_25_NO [M^+^] 235.1936, found 235.1936.

*TBAF treatment of* **23**

To a solution of **23** (70 mg, 0.2 mmol) in THF (5 mL) was added a small amount of TBAF. The reaction was stirred at rt for 15 min and was quenched by adding water (5 mL), and was extracted with ethyl ether (3 × 20 mL). The extract was washed with brine, dried with Na_2_SO_4_, filtered, and concentrated. The crude was purified by flash chromatography (35:65, hexane: EtOAc), yielding 42 mg of (5R*,8aS*, *Z*)-8-butylidene-5-propylhexahydroindolizin-7(1H)-one (**26**) (90%).

Colorless oil, ^1^H NMR (CDCl_3_, 500 MHz) δ 0.88 (t, *J* = 7.3 Hz, 3H), 0.89 (t, *J* = 7.0 Hz, 3H), 1.23 (m, 1H), 1.38 (m, 4H), 1.63 (m, 1H), 1.74 (m, 1H), 1.85 (m, 2H), 2.02 (m, 1H), 2.16 (m, 1H), 2.27 (dd, *J* = 16.7, 16.2 Hz, 1H), 2.41 (q, *J* = 7.2 Hz, 2H), 2.45 (m, 1H), 2.54 (dd, *J* = 16.7, 3.9 Hz, 1H), 2.89 (m, 1H), 3.28 (dt, *J* = 8.8, 2.5 Hz, 1H), 5.72 (t, *J* = 7.1 Hz, 1H); ^13^C NMR (CDCl_3_, 125 MHz) δ 13.8, 14.2, 18.2, 21.3, 22.7, 28.6, 31.2, 37.1, 46.6, 51.2, 60.1, 67.4, 137.3, 139.4, 200.9; HRMS (EI^+^): Calcd for C_15_H_25_NO [M^+^] 235.1936, found 235.1947.

## 4. Conclusions

We have demonstrated that siloxy vinylallenes react with heterodienophiles in a hetero Diels–Alder reaction with total regio and facial selectivity but moderate endo:exo ratio. The cycloadducts were transformed into the indolizidine skeleton by two routes, indicating the usefulness of this reaction. Computational studies comparing the reaction of siloxy vinylallenes with siloxydienes, which have previously shown high endo:exo selectivity, indicate that the major difference is the higher nucleophilicity of the siloxydienes compared to the siloxy vinylallenes. This leads to more compact transition states for the dienes, resulting in a greater relevance of the steric factor present in the reactants. Of the different approaches to the synthesis of indolizidines employed in this and in our previous paper, the one using siloxydienes seems the most efficient in terms of yield and selectivity. The advantage of using siloxy vinylallenes could be the possibility of obtaining more functionalized cycloadducts that could be used for further transformations.

## Figures and Tables

**Table 1:** HOMO and LUMO energies of the reacting compound. Energies are in eV.

Compound	HOMO	LUMO
Siloxy vinylallene	−5.704	−0.586
Siloxydiene	−5.583	−0.533
Δ^1^-Pyrroline	−8.647	−1.464

**Table 2:** Chemical potential (µ), Chemical hardness (η), Global electrophilicity (ω), Global nucleophilicity (N). Energies are in eV.

Compound	µ	η	ω	N
Siloxy vinylallene	−3.26	5.26	1.01	3.11
Siloxydiene	−3.18	5.25	0.96	3.23
Δ^1^-Pyrroline	−5.09	7.72	1.63	0.16

**Table 3:** Global Electron Density Transfer for the transition states studies. Values are in average number of electrons e.

Transition State	GEDT
TSVA-endo	0.309
TSVA-exo	0.276
TSD-endo	0.463
TSD-exo	0.468

**Table 4:** Condensed charges q(N), electrophilic Fukui function, f−, nucleophilic Fukui function f+, radical Fukui function f0, and condensed dual descriptors CDD for the atoms indicated *.

Compound	Atom	q(N)	f−	f+	f0	CDD
Diene	1(C)	−0.0656	0.1602	0.1160	0.1381	−0.0442
	2(C)	0.0599	0.1024	0.0756	0.0890	−0.0268
	3(C)	−0.0614	0.0667	0.1197	0.0932	0.0530
	4(C)	−0.0284	0.1107	0.1497	0.1302	0.0390
Vinylallene	1(C)	−0.0519	0.1379	0.1005	0.1192	−0.0374
	2(C)	0.0528	0.0990	0.0728	0.0859	−0.0262
	3(C)	−0.0566	0.0723	0.1093	0.0908	0.0370
	4(C)	−0.0284	0.1134	0.1443	0.1288	0.0309
	5(C)	−0.0335	0.0736	0.0646	0.0691	−0.0090
Dienophile	1(C)	0.1430	0.0998	0.2732	0.1865	0.1734
	2(N)	−0.0023	0.0973	0.1509	0.1241	0.0536
	3(B)	0.2265	0.0228	0.0369	0.0299	0.0141

* Numbering according to Figure 4.

**Table 5:** Condensed electrophilicity ω_i_, and nucleophilicity N_i_ for the atoms indicated *.

Compound	Atom	ω_i_	N_i_
Diene	1(C)	0.11155	0.56672
	2(C)	0.07270	0.36228
	3(C)	0.11514	0.23615
	4(C)	0.14393	0.39151
Vinylallene	1(C)	0.10143	0.47129
	2(C)	0.07348	0.33844
	3(C)	0.110350	0.24709
	4(C)	0.14558	0.38745
	5(C)	0.06517	0.25138
Dienophile	1(C)	0.45761	0.04737
	2(N)	0.25271	0.04618
	3(B)	0.06185	0.01084

* Numbering according to Figure 4.

## Data Availability

Data is contained within the article or Supplementary Materials.

## Supplementary Materials

The following supporting information can be downloaded at: https://www.mdpi.com/article/10.3390/molecules30234627/s1: Computational section: Figure S1: Stationary points calculated for the reaction of the siloxy diene and Δ^1^-pyrroline, Figure S2: Stationary points calculated for the reaction of the siloxy vinylallene and Δ^1^-pyrroline; Study of the week interactions in the Molecular complexes: Figure S3: NCI scatter graphs of the molecular complexes found. Only the spikes reaching the bottom are relevant. The green color indicates Van der Waals interactions, Figure S4. RDG isosurfaces for the molecular complexes. Green color indicates Van der Waals interactions; Coordinates of the calculated stationary points; NMR spectra of the synthesized compounds.

## Abbreviations

The following abbreviations are used in this manuscript:

CDFT	Conceptual Density Functional Theory
DCM	Dichloromethane
DFT	Density Functional Theory
FMO	Frontier Molecular Orbital
GEDT	Global Electron Density Transfer
NBO	Natural Bond Order
PTAD	4-Phenyl-1,2,4-Triazole-3,5-dione
TBAF	tetrabutylammonium fluoride
TBS	*tert*-Butyl Dimethyl Silyl
