# Supplementary material for: Hetero Diels–Alder Cycloaddition of Siloxy Vinylallenes—Synthesis of the Indolizidine Skeleton: Experimental and Computational Studies
Source: Molecules. 2025 Dec 2;30(23):4627. doi: 10.3390/molecules30234627 (PMC12692913; doi:10.3390/molecules30234627)
Supplement: Supplementary file 1 [file molecules-30-04627-s001.zip › molecules-4014378-supplementary.pdf]

Hetero Diels-Alder cycloaddition of siloxy vinylallenes. Synthesis of the indolizidine skeleton. Experimental and computational studies.

Juan Francisco Rodríguez-Caro [1], Gabriel Vargas-Arana [1,2], María del Mar Afonso [1,*] and José Antonio Palenzuela [1,*]

1 Departamento de Química Orgánica, Instituto Universitario de Bio-Orgánica Antonio González (SINTESTER), Universidad de La Laguna, Avda. Astrofísico Fco. Sánchez 2, 38206 La Laguna, Spain.
2 Current address; Laboratorio de Química de Productos Naturales, Instituto de Investigaciones de la Amazonía Peruana, Iquitos, Perú. gvargas@iiap.gob.pe
* Correspondence: mmafonso@ull.edu.es (MMA); jpalenz@ull.edu.es (JAP)

Supplementary material

Table of contents

- **Computational section**
  - **General Computational Procedures** **1**
  - **Study of the weak interactions in the Molecular complexes** **4**
  - **Coordinates of the calculated stationary points** **6**
- **NMR spectra of the synthesized compounds** **25**

Computational section

General computational procedures

All calculations were performed using Orca 6.1 [19]. The functional used was B3LYP with theD4 correction and the def2-SVP basis set. The solvent (THF) was simulated using the Conductor-like Polarizable Continuum Model (CPCM) as implemented in ORCA. The transition states were found using the NEB protocol as implemented in Orca. All stationary states were confirmed by frequency calculations giving 0 imaginary frequencies for starting materials and final products and one imaginary frequency for transition states. That frequency was checked to correspond to the relevant bond formation. IRC analysis was performed to ascertain that the transition states correspond to the reaction studied. No intermediates were found in those analyses. The Lewis acid was modeled using BF3 coordinated to the nitrogen atom of the dienophile. The 3D images were made with Cylview 1.06 beta [24].

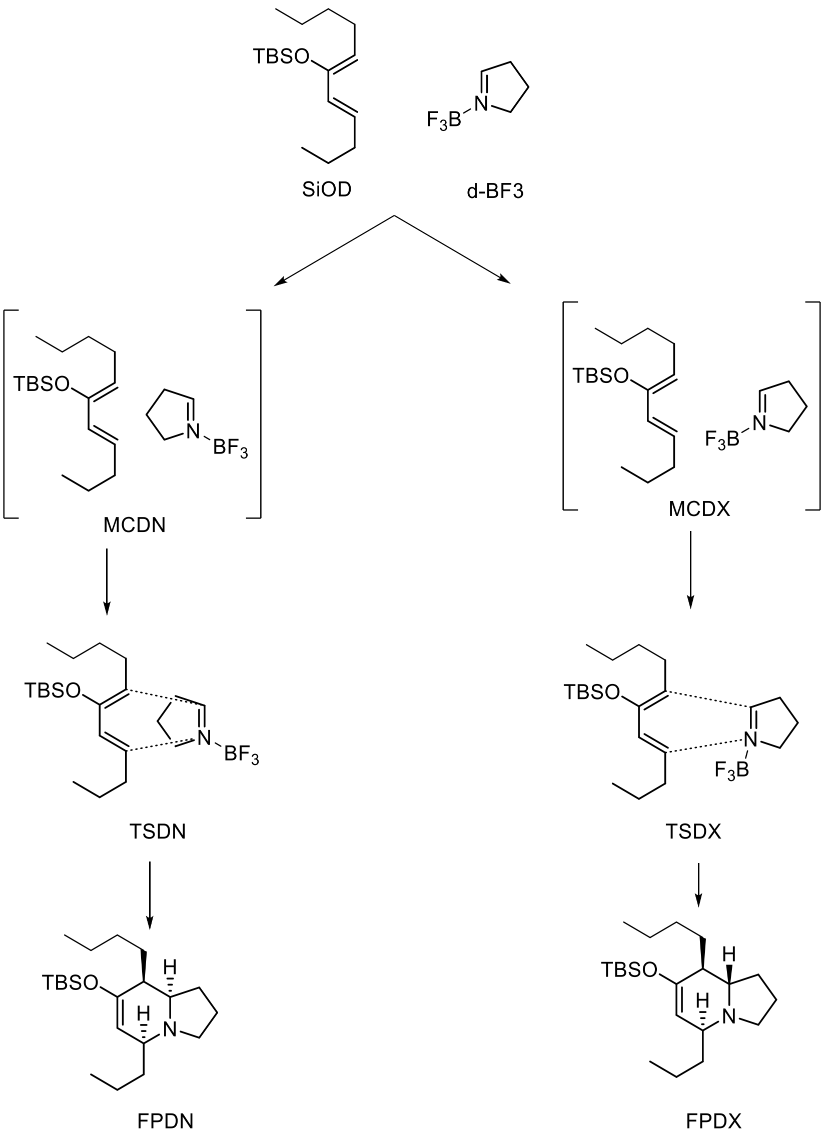

Figure S1. Stationary points calculated for the reaction of the siloxy diene and Δ^1-pyrroline

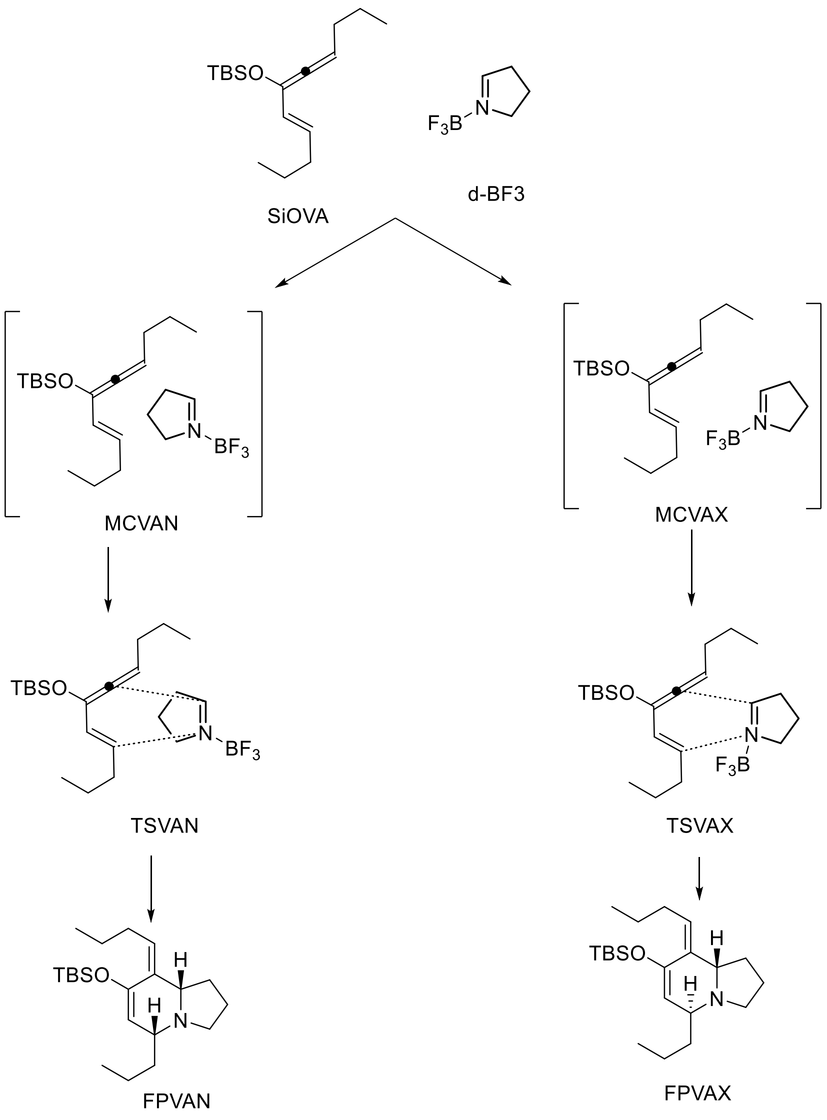

Figure S2. Stationary points calculated for the reaction of the siloxy vinylallene and Δ^1-pyrroline

Study of the week interactions in the Molecular complexes

From the IRC calculations, it was found that in each case, the approach of the dienic reactant to the dienophile resulted in a weakly bonded complex, energetically more stable than the sum of the isolated reactants.

Those complexes were studied using Non-Covalent Interactions (NCI) analysis also known as the reduced density gradient (RDG) method using Multiwfn as the software for the calculations and VMD for the representations.

Figure S3 shows the scatter graphs for each of the molecular complexes found.

In all cases, the relevant interactions are in the Van der Waals region (green color).

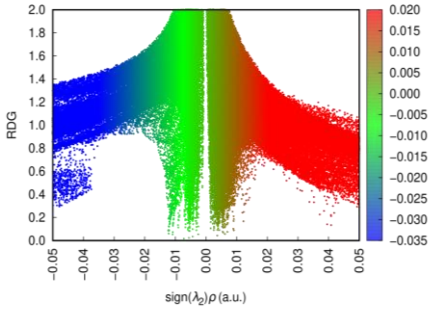

MCDN

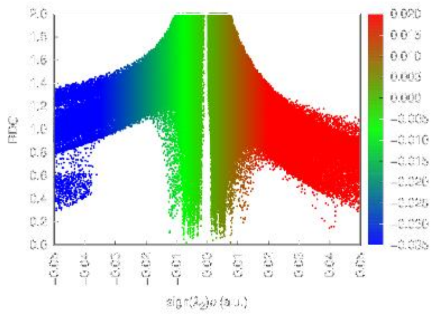

MCDX

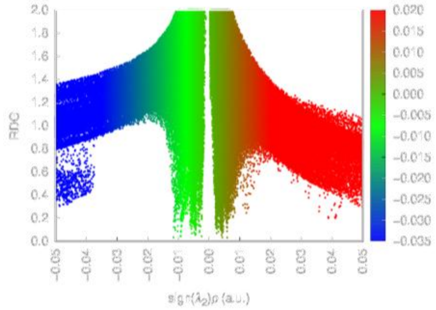

MCVAN

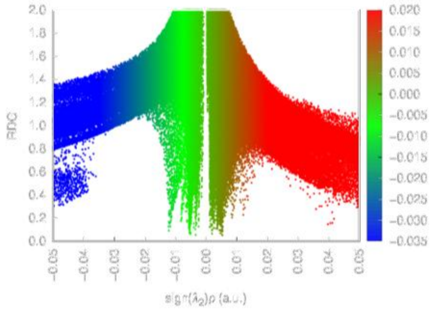

MCVAX

Figure S3. NCI scatter graphs of the molecular complexes found. Only the spikes reacing to the bottom are relent. The green color indicates Van der Waals interactions.

In Figure S4 the RDG isosurfaces are shown. The Van der Waals interactions are mostly between the dienic part and the imine bond of the dienophile.

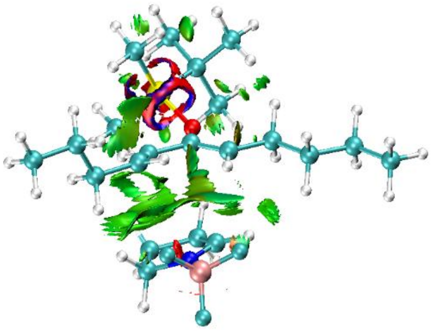

MCD
N

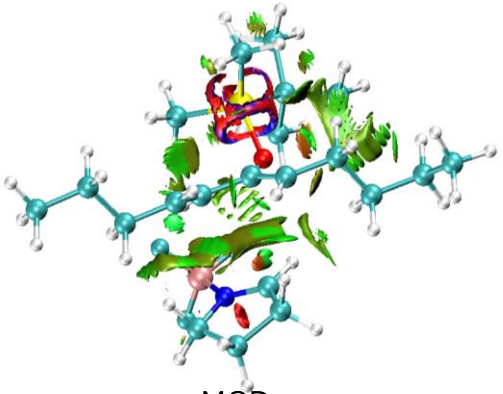

MCD
X

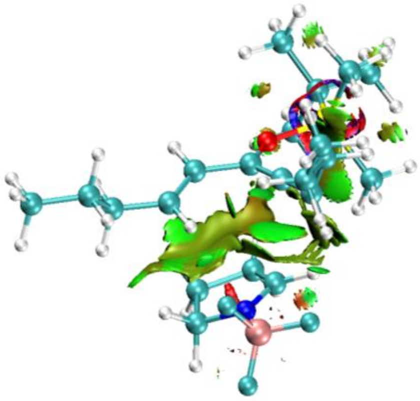

MCVA
N

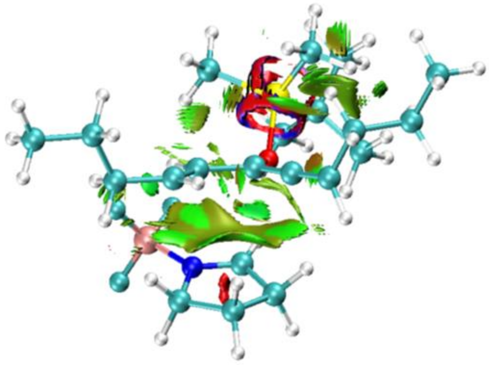

MCVA
X

Figure S4. RDG isosurfaces for the molecular complexes. Green color indicates Van der Waals interactions.

Coordinates of the calculated stationary points

Δ^1-pyrroline + BF3(d-BF3)

Total Enthalpy ... -535.22376542 Eh
Final Entropy Term (T*S) ... 0.04289522 Eh
Final Gibbs free energy ... -535.26666063 Eh

H	-0.39405496270390	-1.63943911913073	1.65915198035853
C	-0.45030554409911	-1.20910802657265	0.65069600538907
H	-1.18671324440533	-1.78972141899599	0.07524374547780
C	0.91153610425506	-1.21669108880011	-0.08116350409757
H	1.77732487993213	-1.24658578128868	0.60713368034338
H	1.04767762925756	-2.04778087110828	-0.79047679163163
C	0.91555723664216	0.10534948104909	-0.77015172060353
H	1.63476759942795	0.43793645386922	-1.52428669075259
N	-0.01612370491951	0.89130982837212	-0.37961249549324
C	-0.85183169490563	0.27795842153854	0.67278134463184
H	-0.61482298354667	0.78445983391809	1.62221952682426
H	-1.91023192041178	0.46500669997893	0.44507010012355
B	-0.26529335294173	2.38759994843906	-0.88729756918286
F	0.72335647674277	2.67364070718882	-1.81502840739701
F	-0.18915427258755	3.20435223166497	0.23112672644374
F	-1.53266824573642	2.41331269987762	-1.45016593043375

Siloxy diene (SiOD)
Total Enthalpy ... -1031.46056673 Eh
Final Entropy Term (T*S) ... 0.08178179 Eh
Final Gibbs free energy ... -1031.54234852 Eh

C	23.49047396716838	-11.96297227916880	-1.94247844635715
C	22.68539754736408	-14.78430205682575	-2.90575523303784
C	22.24075816005246	-12.40358294356740	-2.20092973147348
C	21.87443454042791	-13.80893530557168	-2.46137587867809
C	23.89354420212459	-10.53169903373416	-1.75336500400135
C	22.27137609305650	-16.21135904793103	-3.10781573493793
C	23.01166302272542	-17.19015846617447	-2.17930605425601
O	21.18020945428761	-11.53156948299123	-2.26425443189263
Si	20.14376599622380	-11.07634427168230	-0.99138337035701
C	21.16844433211460	-10.60112020176970	0.51041875292819
C	19.02905714701857	-12.52706087403984	-0.55429060911027
C	19.16563623336892	-9.61005143570649	-1.71381275939412
C	20.14268439942586	-8.50776060353724	-2.16519294177080
C	18.33984143414989	-10.08850144871227	-2.92335754063919
C	18.22159170711399	-9.04901543205781	-0.63217873618604
H	24.28656829932596	-12.71303624873731	-1.89863683607249
H	23.73586758113369	-14.55041189910481	-3.12388164025564
H	20.81753716092648	-14.04548880013361	-2.29061125184962
C	25.00042040140428	-10.09221432493177	-2.72686380252036
H	23.01835929089617	-9.87597016822735	-1.87933307642934
H	24.24729763669854	-10.38344522752598	-0.71605487937281
H	21.18226906106641	-16.31063318773692	-2.95580424518728
H	22.47454971902534	-16.50822772367969	-4.15506136348715
H	24.10096938498047	-17.07291898891470	-2.32447405640101

H	22.81180565497997	-16.90686416151452	-1.13044117247923
H	21.75935351100570	-9.68903727871992	0.33641127818207
H	20.51429973899813	-10.43004751665070	1.38141359348637
H	21.86561834766915	-11.41627563417647	0.76542243938014
H	18.48939116415518	-12.90383691332134	-1.43784276301473
H	19.62128635369614	-13.35663205760076	-0.13396755889291
H	18.28421650878047	-12.22929773820763	0.20252891291439
H	20.83081360057602	-8.86986668580222	-2.94528941311073
H	19.58421203302310	-7.64886623388916	-2.58049532081789
H	20.75240515984684	-8.12994526453798	-1.32788962731642
H	18.98204836935231	-10.52226715606023	-3.70701945962571
H	17.59690357366127	-10.85079567168522	-2.63662960540361
H	17.78870053133898	-9.24147363346138	-3.37169454362006
H	17.63935511490403	-8.20002889624898	-1.03481682550319
H	17.49991972228122	-9.80511892795641	-0.28074180187665
H	18.77682045825871	-8.68019543392897	0.24620454894721
C	22.61338567397391	-18.64752176317945	-2.41012443379179
H	24.65407801771057	-10.27707873739166	-3.75908506182294
H	25.88970181890717	-10.73482482443606	-2.58377383508669
C	25.40888859544097	-8.61998123362174	-2.58971096902376
H	24.51915872046388	-7.98302380564775	-2.75000752058906
H	26.10966841466223	-8.37185669354987	-3.40658153242151
C	26.05693296348303	-8.26250991044545	-1.25003366159286
H	26.94502118253222	-8.88965653283186	-1.05898863680851
H	25.36336973881813	-8.40520164176815	-0.40529457490215
H	26.38232696801813	-7.20940661534594	-1.23260918231800
H	22.83091074936025	-18.96123235225496	-3.44542488760690
H	21.53359364413042	-18.79693936692824	-2.23935081340586
H	23.15664689789183	-19.32634786637464	-1.73278870113962

Siloxy vinylallene (SiOVA)

Total Enthalpy ... -1030.24087452 Eh

Final Entropy Term (T*S) ... 0.07948899 Eh

Final Gibbs free energy ... -1030.32036351 Eh

C	24.66030971797574	-10.57864566525076	-2.55474742002106
C	22.53458159976334	-8.57191418126817	-1.72536405486177
C	24.45433211136772	-9.39889583922235	-3.12176595899038
C	23.32125109368342	-8.50563334888319	-2.81161374046665
C	24.83962889453121	-11.75228634058237	-1.99777801380990
C	21.39343964387438	-7.64578185494015	-1.43252392004274
C	21.59955831637326	-6.82573360404524	-0.14732667720659
O	25.31101730682009	-8.91767079269182	-4.09254273447603
Si	26.73983317051600	-8.05246270083794	-3.73258427714420
C	27.90891432637899	-9.17749692937586	-2.78616553330456
C	26.28826231445299	-6.55992009941044	-2.68315929843556
C	27.41143025405333	-7.57242781131973	-5.44838776853294
C	27.71662402325849	-8.85008408487005	-6.25402825349447
C	26.35740768661988	-6.73448790856755	-6.19706132970627
C	28.70194448877320	-6.74750602973324	-5.27783518915589
H	22.74195608282054	-9.34463262775522	-0.97228909985539
H	23.15106719196130	-7.72456179240356	-3.56242481788905
C	24.30267917696665	-13.06645243940215	-2.51716581525373
H	21.23158943180844	-6.96468154885791	-2.28635256295037
H	20.46382234240845	-8.23737948944913	-1.32070541942479

H	21.79209811511111	-7.51635191216400	0.69380858489632
H	22.51391328010073	-6.21564663937830	-0.25674549141833
H	28.13013681302796	-10.09657271115673	-3.35181828090622
H	28.86122403357242	-8.66275283549016	-2.57544255712977
H	27.46145790387481	-9.46970158549965	-1.82220613162380
H	25.57653941217727	-5.90006867177540	-3.20451512168052
H	25.82294016563805	-6.88285405865567	-1.73725384414329
H	27.18626392668889	-5.97051758917104	-2.43381999131385
H	26.81781341858533	-9.47571951226260	-6.37652456955624
H	28.08493192908226	-8.58861208356210	-7.26299276622226
H	28.49172992535870	-9.46555566246798	-5.76857799408255
H	25.41477807237953	-7.29041306625684	-6.32438537282274
H	26.12538554051612	-5.79608468873876	-5.66660827031840
H	26.72690759103533	-6.46297525897480	-7.20299258499555
H	29.10811148436796	-6.46252838330210	-6.26567829234005
H	28.52413119780129	-5.81627536112737	-4.71459627915259
H	29.48723664261288	-7.31490778148522	-4.75108273274218
C	20.40912453329970	-5.92716130032167	0.18507600223001
H	25.14966551462615	-13.76319411685797	-2.66322362694613
H	23.83747928239747	-12.91505848051541	-3.50537240474336
C	23.29257617536151	-13.71047325761826	-1.55522089247086
H	23.76060022009392	-13.81936072569581	-0.55999517153380
C	22.79100221321484	-15.06929607155060	-2.04204787362087
H	22.44054282975016	-13.02101192500614	-1.41834963110662
H	25.40705507549673	-11.81114875271359	-1.05405682625690
H	22.31623243544884	-14.98569097672746	-3.03450801583908
H	22.04718811493911	-15.49585861856226	-1.34971793372372
H	23.62065154449179	-15.79103257355159	-2.13232190867872
H	20.19755275088782	-5.22490922401117	-0.63939721992586
H	20.59474535225560	-5.33089750912822	1.09310376607314
H	19.49581533139758	-6.52286357740402	0.35349531711720

Molecular complex SiOD + d-BF3 endo (MCDN)

Total Enthalpy ... -1566.70247901 Eh

Final Entropy Term (T*S) ... 0.10353898 Eh

Final Gibbs free energy ... -1566.80601799 Eh

C	1.46389335903928	0.33731910522487	0.28837167280250
C	0.71120323841009	-2.60139794285379	0.69366338410920
C	0.21101885560296	-0.15153889954752	0.42865249905090
C	-0.13359040431877	-1.58099182649831	0.46860723949033
C	1.80028580774453	1.80037274491174	0.22407737939026
C	0.33407238790398	-4.04817854565739	0.63495849993308
C	0.61150273604596	-4.80707757419272	1.94260199891986
O	-0.82942747247923	0.73160231157494	0.44720342461671
Si	-2.33914314327453	0.72748287405066	1.24141885276616
C	-2.16035101786732	-0.12196683227136	2.90636238971362
C	-3.59633098945430	-0.15303784640299	0.15517815263790
C	-2.74895159099632	2.58251612303156	1.41246667124983
C	-1.68751421032307	3.26613811767960	2.29573643837043
C	-2.75360248338318	3.24021781939460	0.01886870569047
C	-4.13706982611219	2.74210519203969	2.06315407635218
H	2.27961398731204	-0.38045533785568	0.18141510354913
H	1.76039430062068	-2.38864335283103	0.92981813770587
H	-1.18158882266004	-1.81678954430356	0.25690586061267
C	3.28878687246863	2.06029670920020	-0.01926126568168

H	1.20410801404766	2.28595327490912	-0.57365258699735
H	1.48162900816165	2.30872601730198	1.15461955516611
H	-0.73069110644517	-4.15100719397248	0.36053371452900
H	0.92053139139787	-4.51963147810150	-0.17571378720569
H	1.67478371912949	-4.67836920061756	2.21488039090613
H	0.02929628109844	-4.34334164347850	2.75911214968686
H	-1.35546528472901	0.33794356055809	3.50202202839757
H	-3.09912004745129	-0.04232862591112	3.47925527158637
H	-1.92678501388157	-1.19121628099792	2.78257516384570
H	-3.59319599259801	0.26132790896614	-0.86584558069875
H	-3.38123091951704	-1.23140094043899	0.08740922423154
H	-4.61272657790535	-0.03917367769042	0.56707723744931
H	-0.67631520004475	3.16017258786560	1.87204099061048
H	-1.90221466455685	4.34675319285114	2.38503778926010
H	-1.67117848406657	2.84855482662940	3.31586933112517
H	-1.77463875340273	3.14458442828050	-0.47754775296429
H	-3.51303271163006	2.79448768770426	-0.64471116171951
H	-2.98240911215203	4.31831942766797	0.10489206496259
H	-4.38333466354908	3.81327085927639	2.17974939454108
H	-4.93456726216161	2.28615664620951	1.45355362209143
H	-4.17710349478572	2.28555742002835	3.06620889305093
C	0.27950333582956	-6.29576992409769	1.84674293340200
H	3.60760193156930	1.52816317680984	-0.93462953788977
H	3.87419209527260	1.60861294264720	0.80320730399170
C	3.63986195365821	3.54352743712238	-0.14017365504488
H	3.04946493641318	3.98830026079904	-0.96266757689056
C	5.12817180694610	3.79598158009597	-0.38145283773147
H	3.31715272061851	4.06772561273035	0.77841309097952
C	-1.11161459919284	-1.27046145149797	-2.90517171239650
H	-1.78238643090272	-1.33072204688712	-3.77257956069540
C	0.03941157478412	-2.29473117271499	-3.00046561739657
H	0.17087295876233	-2.71309560153618	-4.01134491561993
H	-0.05272857196984	-3.13453096633490	-2.29903645179982
N	1.25293383814545	-1.52346111402759	-2.67299420143185
C	1.01622026140396	-0.26677742051801	-2.56817461462790
H	1.83242232934933	0.43445060875518	-2.39202715432829
H	-1.71480299588551	-1.45309193354101	-2.00599264229872
C	-0.40618979326697	0.10343622583925	-2.80590345025933
H	-0.46282062781223	0.69459330259216	-3.73789504203594
H	-0.78797882614401	0.74572871945723	-1.99764613805432
B	2.68153171924846	-2.20601833761293	-2.54496025015340
F	2.55124151526216	-3.30133692963106	-1.69782761151269
F	3.55154260675311	-1.25319260623697	-2.02291742727075
F	3.06554477029461	-2.60740964470984	-3.81891557478034
H	5.46858390723857	3.30699607997308	-1.31027092368415
H	5.73940539154805	3.39416335341641	0.44473079936592
H	5.34959429129066	4.87220625011711	-0.46879559441814
H	0.87197606906119	-6.78406630008039	1.05432074064459
H	-0.78601552926030	-6.45186921516095	1.60608434327414
H	0.48905665174748	-6.81877297750110	2.79390810552866

Transition State SiOD + d-BF3 endo (TSDN)

Total Enthalpy ... -1566.68989937 Eh

Final Entropy Term (T*S) ... 0.09768791 Eh

Final Gibbs free energy ... -1566.78758728 Eh

Imaginary frequency ... -488.41 cm^{-1}

C	1.28493505166404	0.18720213744001	-0.00739527947332
C	0.60384936211349	-2.62262936152545	0.55294948903750
C	0.04728198644981	-0.22731674805005	0.53342074627936
C	-0.27514131108523	-1.60646111087417	0.76000575713374
C	1.64180126781222	1.66144645118449	0.00513002483609
C	0.24558845253155	-4.06724206213761	0.59569938302209
C	1.24438253219212	-4.91375301733334	1.39908324339897
O	-0.88073605096573	0.70497970554214	0.67555969619137
Si	-2.47878103688178	0.82598377159840	1.32978269102676
C	-2.43898228594042	0.13664390631328	3.07148259756831
C	-3.66432462731914	-0.11346753146172	0.22322652940539
C	-2.77172203873457	2.70471409312964	1.27709244224777
C	-1.77166058105159	3.41297339878977	2.21172781663146
C	-2.57737739009985	3.21838609195145	-0.16325823889759
C	-4.21182911119625	2.99986266265641	1.74317189947509
H	2.11915916117933	-0.48592767380217	0.19751839326556
H	1.65597579244949	-2.40182123282078	0.37029848956151
H	-1.31135624473510	-1.84756656376158	1.01187512461757
C	3.14210982947994	1.91518295880146	-0.15483516009319
H	1.08627944891126	2.19422502687751	-0.78753678512679
H	1.29228294613001	2.11265869713794	0.95016990897280
H	-0.78305327471627	-4.20299113406724	0.97151881512262
H	0.25930290799088	-4.43039017506241	-0.44936995219630
H	2.25290557892527	-4.75560555468010	0.97906930071029
H	1.27968222999546	-4.54743813733235	2.44046584867629
H	-1.62621979575595	0.59238886968765	3.65905503650854
H	-3.39248207118215	0.35211959067204	3.58188160623501
H	-2.29655107184928	-0.95544385940024	3.07264335640106
H	-3.63499667912090	0.26569790431803	-0.81010660891011
H	-3.43881641571811	-1.19105234370590	0.20311290731340
H	-4.69281888345970	0.00656985842029	0.60256757183656
H	-0.72840045613386	3.20201298468626	1.92683542472506
H	-1.91666192438240	4.50733576739640	2.16340039654640
H	-1.90419186894177	3.10887892666464	3.26289580276713
H	-1.55322226735415	3.03428036581049	-0.52423467781855
H	-3.27612548457105	2.74190210087501	-0.87041233273543
H	-2.75730911251950	4.30783788615650	-0.20528931345870
H	-4.39475241944129	4.08950138386677	1.74252641709603
H	-4.96311075940486	2.53977121975207	1.08042784427826
H	-4.39727505432170	2.63667560328785	2.76787039920484
C	0.90259867175552	-6.40245196167140	1.38071261818083
H	3.51221568315056	1.42479861626462	-1.07367532729003
H	3.68131955899166	1.42966842369513	0.67933910581066
C	3.50007805329853	3.40168744292858	-0.19763777109489
H	2.96390920236223	3.87772316743090	-1.03925159756474
C	5.00072398662764	3.65923900048082	-0.33179642101220
H	3.11866180950423	3.89110777394897	0.71752115301829
C	-1.05059798686499	-0.97080547909486	-2.54122973731076
H	-1.77129014988689	-0.92001093240086	-3.36999853731864
C	-0.09235191463676	-2.16381619647701	-2.66472757864138
H	0.10034173897098	-2.41558095167753	-3.72518520392969
H	-0.47048609595486	-3.07509414565965	-2.17787957539797
N	1.15402439795538	-1.69424378864299	-2.04795795745472

C	1.15758171079264	-0.34324025292968	-1.87331718527100
H	2.10735498832586	0.15909792962192	-2.08698282775663
H	-1.62577063285479	-1.04220550129375	-1.60797216313625
C	-0.10298908390366	0.23890910676038	-2.49341002556400
H	0.14650502097414	0.58395431486904	-3.51214726282669
H	-0.50973050171432	1.10116929813508	-1.94731514116120
B	2.45374538945058	-2.51825266637853	-2.12251892040100
F	2.15414605573351	-3.87459294951907	-1.92174526961871
F	3.33408262343556	-2.05782442031368	-1.11754055893822
F	3.06510122364441	-2.35733176485033	-3.37685811428930
H	5.40175799520770	3.20703259692923	-1.25498427779571
H	5.55680278936482	3.22454147829336	0.51633114373621
H	5.22623114121746	4.73766964208892	-0.36221724577129
H	0.89293374405790	-6.79320872483609	0.34909605375968
H	-0.09372566758446	-6.59029189583311	1.81590457790928
H	1.63587791763714	-6.98969201686978	1.95653743574722

Molecular complex SiOD + d-BF3 exo (MCXN)

Total Enthalpy ... -1566.70387894Eh

Final Entropy Term (T*S) ... 0.10014001 Eh

Final Gibbs free energy ... -1566.80401895 Eh

C	1.92106396995185	0.11791628816791	1.05690591964632
C	1.14221181040578	-2.87719376074460	0.93146168098266
C	0.75433451320350	-0.40497324755480	0.62258189151348
C	0.48577331877761	-1.83446218219142	0.39385464030482
C	2.15238073488505	1.58637227253168	1.27571038476083
C	0.83961451855788	-4.31240600006732	0.61992204126664
C	0.44605145306685	-5.13350614449868	1.85866283965016
O	-0.27940065334798	0.43348645448962	0.29053020629606
Si	-1.77996380018176	0.56911775474854	1.09551604888547
C	-1.43102253935593	0.60066762492064	2.94223880616592
C	-2.87353898686418	-0.89532305425546	0.66126193557370
C	-2.50695314489758	2.21514402123045	0.46602066781910
C	-1.45511700859812	3.33359370396609	0.59114175641305
C	-2.93244260067571	2.07654807443300	-1.00799618053447
C	-3.73926585126168	2.57080685390078	1.32225861611661
H	2.75697651923043	-0.56967787697782	1.21416686949426
H	1.94595944535805	-2.69567434114378	1.65738288628174
H	-0.33590528362649	-2.03663142655434	-0.29921023221063
C	2.52135359701410	2.34373371446472	-0.01422436804432
H	1.24516561942997	2.04975390475003	1.69928438699634
H	2.95353107967001	1.71765816552282	2.02155743799201
H	0.03592635303422	-4.36764495313868	-0.13501613223503
H	1.73338787878489	-4.78095891611719	0.16229643153436
H	1.24931477836923	-5.05559107330060	2.61376653984169
H	-0.44956262366159	-4.67860795153071	2.31836647667085
H	-0.83421326178827	1.48131463642212	3.22906702138267
H	-2.37136203240655	0.61923251293410	3.51740548756224
H	-0.87234601054002	-0.30273488981503	3.23840246798642
H	-2.52373694232018	-1.80709533240067	1.17211257264777
H	-3.91407354926914	-0.70859954629753	0.97562168759605
H	-2.86308307438971	-1.08990547028553	-0.42194322983814
H	-0.57872108232275	3.13577219889965	-0.04433874043638
H	-1.88771170073283	4.30148215949728	0.27771066867368

H	-1.09742352313799	3.45269206486010	1.62744931806481
H	-2.09500011966829	1.77494432208714	-1.65477170999230
H	-3.73097519930918	1.32719134817562	-1.13529204726512
H	-3.32223942571813	3.04082633678676	-1.38353650810672
H	-4.20380993553805	3.50305548772205	0.95216367480792
H	-4.51038364330715	1.78295112190676	1.28407162845481
H	-3.47401854236836	2.73205682192327	2.38008041079934
C	0.17723107060471	-6.60354079642593	1.53900322408985
H	1.72188345352627	2.17035202159054	-0.75418756675473
H	3.44111634981691	1.90646150541284	-0.44511271416358
C	2.71005462056370	3.85390760161660	0.17291433449346
H	1.78017866354677	4.28240832733491	0.59018076205236
H	2.83623692482131	4.31764802419990	-0.82169330470686
C	3.89559318939158	4.24219986311598	1.05921512778247
C	2.85591347988432	-2.23145236152059	-2.93139975079531
H	3.46819670809758	-2.87821565441397	-2.28924127230594
C	1.39349204467895	-2.71237411194783	-3.00288773202288
H	1.10599215799967	-3.36336301688163	-2.16341699047548
H	1.13328531134744	-3.22919343400184	-3.93674481427037
N	0.59617658353214	-1.47398365259851	-2.90017971573366
C	1.31155181407210	-0.46696309609873	-2.56241725559942
H	0.84782056518269	0.51028129111360	-2.40753003125163
H	3.30513216321221	-2.23008385006519	-3.93538958494803
C	2.75508887043446	-0.78705475989719	-2.38908424782499
H	2.99457764738195	-0.69991194137533	-1.31519806185389
H	3.39103078212070	-0.05515254053675	-2.91179482557327
B	-0.97629734576400	-1.47537168487354	-3.15873544209353
F	-1.17135266472389	-1.93681168014617	-4.45125389532116
F	-1.54332204576033	-2.34408300043709	-2.22668524873463
F	-1.43066762793720	-0.17547409765539	-2.99513052225176
H	4.83792851181900	3.81984386623151	0.66888981362307
H	3.77419969331738	3.87963523218023	2.09309639652215
H	4.01606198887460	5.33683602345062	1.10836205197121
H	1.06979561194387	-7.08673550809067	1.10601892780153
H	-0.64306813548678	-6.70900580294739	0.80864124868574
H	-0.10448544094998	-7.16812444379997	2.44258683614066

Transition State SiOD + d-BF3 exo (TSDX)

Total Enthalpy	...	-1566.68059516 Eh
Final Entropy Term (T*S)	...	0.09856112 Eh
Final Gibbs free energy	...	-1566.77915628 Eh
Imaginary frequency	...	-332.72 cm^{-1}

C	1.68363834122065	-0.20622439443361	0.20277008046780
C	0.61151451840801	-2.94493687403024	0.51912344031607
C	0.34561628726185	-0.49240875770165	0.56416071347749
C	-0.14568709748610	-1.83515890524623	0.69842916418217
C	2.22837937309436	1.20479914227109	0.39239656809819
C	0.09068682819038	-4.34004082170415	0.55682113099908
C	0.68015934119518	-5.15983061051561	1.71861454931908
O	-0.49124398897889	0.52281625043868	0.68057844681122
Si	-2.16196865475071	0.69781024547391	1.12920500914596
C	-2.30902287012194	0.11554077568989	2.90494843493257
C	-3.24396423331645	-0.28243347840780	-0.04432295451249
C	-2.43362168984374	2.57261765062219	0.95073496173264

C	-1.39076847201110	3.34132096315838	1.78434852529285
C	-2.31193800315561	2.97345001324842	-0.53257652738203
C	-3.84875065159909	2.91769090294239	1.45912199239227
H	2.37804624340218	-0.99059500190284	0.51069258400849
H	1.69479559912443	-2.85065877603283	0.41216015255131
H	-1.21872523515510	-1.96186260170688	0.85429306006573
C	1.78991749933239	2.27611590874663	-0.61793425072973
H	1.93687434187127	1.54697728479594	1.40306457171529
H	3.32735806026021	1.14373529497801	0.40294926732025
H	-1.01056111083574	-4.33659370487368	0.60999790439609
H	0.35928446979133	-4.82612288919320	-0.39743244226218
H	1.78304286312136	-5.13035018769410	1.65927671165856
H	0.41145835791998	-4.67713594384068	2.67481860088028
H	-1.64144659727241	0.68758986205974	3.56884507633562
H	-3.34439965378921	0.25033685458972	3.25931777656006
H	-2.05788616335355	-0.95289299307909	2.99806191375848
H	-3.30034639087588	-1.34505112328652	0.23864565889183
H	-4.26768801027811	0.12627172007727	-0.02765978608577
H	-2.85349041665856	-0.22617784697080	-1.07058426495028
H	-0.36324500988378	3.10814170603041	1.46808971574116
H	-1.53942970834944	4.43026232490662	1.66875698912193
H	-1.47376857387007	3.11035385433621	2.85920819754056
H	-1.31740889767995	2.73438096608693	-0.94042482484946
H	-3.06331627245805	2.46531601453975	-1.15850437778366
H	-2.46570250092810	4.06183927922523	-0.64663062561292
H	-4.03508961608135	4.00112353091294	1.34821342521083
H	-4.63468312089264	2.39093823718781	0.89270571665145
H	-3.97657310785420	2.66954494973647	2.52568426817004
C	0.19967014728243	-6.61046112109384	1.71375508794449
H	0.69922235883712	2.22579636655924	-0.74903926332040
H	2.23273932690288	2.06701968836910	-1.60621353939320
C	2.17716567404730	3.70001432311083	-0.20109876979194
H	1.71644939872087	3.92848290977898	0.77741165073909
H	1.72723873562270	4.40876933545343	-0.91859420371643
C	3.68504925141434	3.95178726915860	-0.12721502575558
C	3.55426519486181	-1.87190270728070	-2.83817025944963
H	4.57098855965195	-2.26843676668219	-2.69793861271363
C	2.45584160531951	-2.81867655467779	-2.33728092228834
H	2.78706968722264	-3.37317707768874	-1.43833667124429
H	2.14289605760108	-3.56292482490183	-3.08307356314631
N	1.34187151251878	-1.91709187658775	-2.02651938207708
C	1.78621215039256	-0.66727052260099	-1.72857857650119
H	1.13294601337760	0.14699647523337	-2.04813451245472
H	3.40521035843235	-1.68018870813867	-3.91318043740230
C	3.28956666893181	-0.58557482142627	-2.03126477598691
H	3.89002961089211	-0.57583774027105	-1.10918173270653
H	3.53380185332224	0.33299069521539	-2.58445414121762
B	-0.06350209649712	-2.15403304061865	-2.61701586505748
F	-0.00261620783748	-2.04366109285541	-4.02033460001749
F	-0.52834665681721	-3.43497846710389	-2.29060059329043
F	-0.92803349789981	-1.16178659821217	-2.11200729713208
H	4.17255312420351	3.72417941165462	-1.09114351373570
H	4.16982437979558	3.33001181163521	0.64318984618650
H	3.90279905717226	5.00444846225426	0.11657962781485
H	0.48663010026606	-7.12001776954613	0.77844881993223

H	-0.89873716845033	-6.66704995315719	1.79831380465851
H	0.63077872399870	-7.17870592701448	2.55370286754676

Molecular complex MCSiOVA + d-BF3 endo (MCVAN)

Total Enthalpy ... -1565.48517954 Eh

Final Entropy Term (T*S) ... 0.10159590 Eh

Final Gibbs free energy ... -1565.58677544 Eh

C	0.57737111499703	0.92398140521852	-0.88105349784592
C	-0.00627673064695	-0.25299570464475	-0.69596511171507
C	0.67304256521724	-1.54753764209284	-0.88667271443841
C	2.00078635838712	-1.73263378940294	-0.96325183499718
N	2.57017830690721	0.05813278145315	1.97394479997002
C	1.33706009082581	0.38407705948071	2.09843043017273
C	2.82237920776826	-1.33873680631397	2.37771851705903
C	1.16524348958981	2.07094627506622	-1.12282617471307
H	-0.00804681167055	-2.40455974265367	-0.94703279093220
C	1.18556743953835	2.78064739349405	-2.45581878648291
H	1.73247315434000	2.55770784388228	-0.31483200286627
C	2.61099372123345	2.91062790953119	-3.01507770000559
H	0.75723650646138	3.79280200760984	-2.32810349523299
H	0.54348705355118	2.24789490097804	-3.17670700255255
H	3.24610052314701	3.41998325148741	-2.26813435623246
C	2.66401286740556	3.66372823220591	-4.34362278387984
H	3.03844245312730	1.89957714561235	-3.13674297639068
C	2.66938805243893	-3.05696646565126	-1.16948974270550
H	2.66328552069668	-0.86431152941457	-0.86518276762860
C	3.55853159606733	-3.09097331131164	-2.42373454408570
H	1.91443259764628	-3.86082113876393	-1.22164922085145
H	3.30674709302379	-3.28054182093525	-0.29100884218887
H	2.93505595652460	-2.87793436578809	-3.31034191036311
C	4.28367605939831	-4.42385431549439	-2.60475265203010
H	4.29452814507504	-2.26906574502002	-2.36331786605679
O	-1.30434996116846	-0.39753232676542	-0.27566795598352
Si	-2.49672575684683	0.81719404536930	-0.08204596745103
C	-4.03435381445772	-0.19797643056961	0.40789212133815
C	-2.72312785463719	1.72418880986078	-1.70976981725321
C	-1.98004325261189	2.00214902365106	1.28288526787191
H	-1.80609951764102	1.47397799144104	2.23384060918291
H	-2.77224852806699	2.75059189954638	1.45163390379604
H	-1.06027199558802	2.54162920332444	1.00893375351478
C	-4.43372878082748	-1.12518241393062	-0.75582941529333
C	-5.19743085618255	0.76222786072907	0.72558527366596
C	-3.71612875954924	-1.04914613636071	1.65270566569105
H	-2.89242164108122	1.01798357054594	-2.53840441172883
H	-1.83725796463500	2.33198951618492	-1.94993738269220
H	-3.59305365805479	2.39965778823088	-1.65265563480746
H	-5.45648290120427	1.39845771576074	-0.13724710636477
H	-4.96281150373545	1.42554358453866	1.57438266801567
H	-6.10351978398371	0.18967703852052	0.99551702555901
H	-2.89611665918285	-1.75878097066740	1.45917649571351
H	-4.60388142416222	-1.63554421546483	1.95278398255653
H	-3.42593282080393	-0.42668759921960	2.51532324866025
H	-4.71709780973533	-0.55591538423508	-1.65630034489679
H	-5.30270228193506	-1.74621265415320	-0.47079728439097

H	-3.61271028046987	-1.80647715979888	-1.03204522383792
C	0.46821725914045	-0.71650557535725	2.59791868935044
H	1.01352744415045	1.40589326131321	1.89151166175181
H	-0.41450291302670	-0.84093439803846	1.95279179927817
H	0.08899989907322	-0.43771571084959	3.59781944389112
C	1.41999169234509	-1.93718764558515	2.61174284115752
H	3.44937264973793	-1.31506583836837	3.28257100860032
H	3.39700108530363	-1.84127675441195	1.58775923389568
H	1.16044930244462	-2.62881227615523	1.79922758749798
H	1.36908210427152	-2.49142734507373	3.55829927542305
B	3.75269092148590	1.01426577025421	1.50403658780137
F	3.24537394048169	2.30767462808921	1.43150122097956
F	4.75531917030998	0.90341100802681	2.45635288856728
F	4.18965723415779	0.56555971958744	0.26099391960800
H	3.69607752727174	3.73782172702193	-4.72305067976216
H	2.27101182815667	4.68928378944302	-4.23725344132011
H	2.05928049472555	3.15582576990042	-5.11420619106054
H	4.91369770437236	-4.42058216640513	-3.50896663064904
H	3.56782442174844	-5.25834038684124	-2.69814178277201
H	4.93601970936051	-4.64260416162084	-1.74207187611067

Transition State SiOVA + d-BF3 endo (TSVAN)

Total Enthalpy	...	-1565.47480288 Eh
Final Entropy Term (T*S)	...	0.09798646 Eh
Final Gibbs free energy	...	-1565.57278934 Eh
Imaginary frequency	...	-240.91 cm^{-1}

C	0.76583371240998	0.96850093067884	-0.09923129353472
C	0.05744861934001	-0.18256431168385	-0.35278932954425
C	0.72021177358519	-1.40208518506359	-0.75934661973511
C	2.04310962322030	-1.46273148134715	-1.03640472489809
N	2.64160085341918	-0.37224610722139	1.64412166691812
C	1.71974129817131	0.57469942596866	1.77173099139683
C	2.25817366557308	-1.60952154868889	2.34485810902741
C	1.25641000004568	2.07601646029794	-0.62996665803323
H	0.09067294710054	-2.29667101643045	-0.80502104153191
C	1.09434933027508	2.49910614420455	-2.06652268316129
H	1.89729988302159	2.72611441834941	-0.01770736760034
C	2.44409650425693	2.52187249785919	-2.80372867113452
H	0.64834959938005	3.51083782003164	-2.10345284370692
H	0.39882471291274	1.82005049330620	-2.58516315302558
H	3.14541697833057	3.17689690641123	-2.25665601234811
C	2.31968151954340	2.98810102306112	-4.25354916620246
H	2.88439251238924	1.51014301234011	-2.76762693372223
C	2.78746171109896	-2.70007922280311	-1.40452143503717
H	2.62628019745840	-0.53932058996770	-0.99282006094249
C	3.76194777475905	-2.48375864046024	-2.57184834810572
H	2.08779896282291	-3.52566960378265	-1.62106197396982
H	3.37975680901897	-3.00041567360262	-0.51976846708833
H	3.19876069165643	-2.14596989823684	-3.46020199574962
C	4.56824847641139	-3.73616357083652	-2.91053148999652
H	4.44519464102966	-1.65834004743281	-2.30593935958983
O	-1.23958592863294	-0.31284520407284	-0.06137199988829
Si	-2.52514341712213	0.85126067885096	-0.01862856263927

C	-4.01971776043189	-0.25652530046460	0.37849082709503
C	-2.61752493237501	1.63838686342247	-1.71572800540754
C	-2.22694313254285	2.14404456272009	1.30557160816188
H	-2.23547171462432	1.71438291160073	2.31903553300372
H	-3.03102854508232	2.89716214276153	1.25133002269104
H	-1.26708478894639	2.65872138763320	1.14880386887776
C	-4.22263264717840	-1.28139257973974	-0.75441501282154
C	-5.27710823444949	0.62607694786273	0.50900549479703
C	-3.77102051454086	-1.00094782827129	1.70498100482901
H	-2.59648296248876	0.87768433828156	-2.51201026135857
H	-1.77629926098483	2.33016384976607	-1.87379446599273
H	-3.55256738869120	2.21444830869996	-1.81434022865805
H	-5.49127807544100	1.18018577742259	-0.42012041851121
H	-5.18323692144573	1.36050777998423	1.32599228277829
H	-6.16017108522807	-0.00062399928441	0.72910460239702
H	-2.87538454420451	-1.64035721825972	1.65062895016608
H	-4.63247337911342	-1.65049555039858	1.94425418045312
H	-3.63874297419592	-0.30545724141634	2.55007137869782
H	-4.43970120720961	-0.79166228175985	-1.71792382982760
H	-5.07573185496376	-1.94354064542922	-0.52016703867008
H	-3.33283143574920	-1.91681422130505	-0.89065871530692
C	0.64467485564990	0.13576218693564	2.73610448174805
H	2.03644055463803	1.61740372870186	1.70314228670691
H	-0.35703334120586	0.50402202176302	2.48859099618466
H	0.90859473873319	0.54590827068158	3.72868320240360
C	0.78198553809429	-1.39557124918397	2.71238236507602
H	2.89381832473208	-1.70870853996156	3.24338671309902
H	2.43373101960616	-2.49342663912851	1.71681214253769
H	0.12777897708537	-1.82193550106895	1.93775195202999
H	0.51540593779472	-1.86473634203279	3.66964380363368
B	4.11112553756158	-0.11385677660908	1.19021676480311
F	4.10852733366242	0.87237057747982	0.19529798361032
F	4.85618681550347	0.32010222181662	2.29378564987478
F	4.64664740861013	-1.31230414564509	0.70328231006441
H	3.29845680836450	2.98994929920880	-4.75993141136582
H	1.91018379921955	4.01108188256097	-4.30992837173666
H	1.64605573901190	2.32954387843349	-4.82790499880125
H	5.26168638108442	-3.55390205473339	-3.74728783452153
H	3.90778648609645	-4.57185622094157	-3.19822825691730
H	5.16545699416965	-4.06870231183243	-2.04455213197974

Molecular complex MCSiOVA + d-BF3 exo (MCVAX)

Total Enthalpy	...	-1565.48222174 Eh
Final Entropy Term (T*S)	...	0.10179850 Eh
Final Gibbs free energy	...	-1565.58402023 Eh

C	23.39314609859655	-11.42012110454476	-3.71096872402371
C	23.30175130567531	-14.45252299146823	-4.05066690023965
C	22.31790689555454	-12.18203499642228	-3.57815830003909
C	22.27399653637927	-13.64525377643985	-3.74008297246680
C	24.45873131425475	-10.66327770081781	-3.80788151576683
C	23.17917005395252	-15.93609636580659	-4.22751314765424
C	23.96243854517775	-16.74248359705911	-3.17743235912126
O	21.09417376745368	-11.59501574070664	-3.31548269748924
Si	20.45877265531048	-11.37121043956735	-1.74018993527428

C	21.87030067780471	-10.82181964177448	-0.62844251767450
C	19.73635759345722	-13.00183746756211	-1.15439254041643
C	19.12892835060758	-10.01495744854020	-1.93140732236670
C	19.65031751840704	-8.90700507761246	-2.86574453980840
C	17.82616002270314	-10.61031991042890	-2.49961557243439
C	18.83905992084568	-9.41404311787288	-0.54014797502011
H	24.30323106108637	-14.01917300876939	-4.17834162568036
H	21.27636241304589	-14.07622046960615	-3.62436822967966
C	25.35578073947991	-10.27572569839123	-2.65386707331307
H	22.11459250495405	-16.22469091105513	-4.20213058919418
H	23.55799473700093	-16.21279081571523	-5.23073531892600
H	25.02198400440137	-16.42849256711253	-3.19466051497649
H	23.58319760681741	-16.48112195964380	-2.17345069968153
H	22.30079687823295	-9.86577252186140	-0.96745393076033
H	21.52000864051440	-10.69547272143472	0.40897973878753
H	22.67499654701342	-11.57456768742335	-0.62583833492006
H	19.04456855803583	-13.41237284077503	-1.90656672460868
H	20.53532854025651	-13.74127815475360	-0.98052624407925
H	19.18360242250901	-12.86914055917991	-0.20926956956818
H	19.82563567556547	-9.28271355381029	-3.88545479847542
H	18.91608106662021	-8.08304178323516	-2.93010847005156
H	20.59894251115486	-8.47640057997282	-2.50277692106591
H	17.98185165831759	-11.15222592184226	-3.44450043582279
H	17.36575307912095	-11.32158441016561	-1.79470401836004
H	17.08929347112200	-9.80776789297588	-2.68712557168340
H	18.02302822953016	-8.67169605932548	-0.60894799389505
H	18.52192943657812	-10.18278334054337	0.18525359395314
H	19.71944463903251	-8.89980505405780	-0.12211260955049
C	21.90307997215962	-12.41861633021939	-7.11475775374316
H	22.80384856861406	-12.54495676713352	-6.49867584755631
C	20.96763080207844	-13.63058706756996	-6.93565887727744
H	21.45331231202818	-14.50569706167409	-6.48451878578463
H	20.48077685592340	-13.95394600497421	-7.86979004322224
N	19.91064473745810	-13.14514561806100	-6.02578290162882
C	19.96628592640138	-11.87677948079181	-5.85549450568485
H	19.22423396157309	-11.36563735401865	-5.24177087173461
B	18.83381867702035	-14.12822655219606	-5.39150542217719
F	19.53399483797342	-15.13899338521573	-4.73510735246705
F	18.07536298397234	-14.64118053637223	-6.43282653764811
F	18.06193383012378	-13.38770278696840	-4.50171396884264
H	22.21805624598024	-12.29363580945641	-8.15913191575032
C	21.07590633404917	-11.21860840946961	-6.59714030970535
H	21.63358149733427	-10.53338068932622	-5.94096308045714
H	20.63845153191209	-10.60333504500114	-7.40502480885133
C	23.86545152028313	-18.25217579421949	-3.39450635198457
H	25.10619007336592	-10.88329515844027	-1.76849884486568
C	25.24625221146182	-8.78285040501473	-2.30680933285598
H	26.40386200104707	-10.50652722872646	-2.92053092284503
H	24.19882171805472	-8.55765615784566	-2.03755553605761
H	25.46535890906265	-8.18332422469251	-3.20883019104345
C	26.17697777414074	-8.36751653963132	-1.16806364987728
H	24.72072700573764	-10.22791088060550	-4.78648999223098
H	27.23284836062997	-8.55661191661254	-1.42623609266085
H	25.95494358433940	-8.93272191639462	-0.24694740085534
H	26.07531053893581	-7.29494555831997	-0.93612294049817

H	24.27201366336438	-18.54013877406245	-4.37900095215052
H	22.81638371796372	-18.59178452483530	-3.35813534719028
H	24.42515417043512	-18.80776413387347	-2.62468610100471

Transition State SiOVA + d-BF3 exo (TSVAX)

Total Enthalpy	...	-1565.46708755 Eh
Final Entropy Term (T*S)	...	0.09870437 Eh
Final Gibbs free energy	...	-1565.56579192 Eh
Imaginary frequency	...	-220.78 cm^{-1}

C	22.72205734738828	-11.49258062947984	-4.28108616938542
C	22.64456774130604	-14.36831940341926	-3.83702107217223
C	22.04667194268416	-12.06355319404566	-3.22518788394781
C	22.04050439479310	-13.49360427421775	-3.00425709834747
C	23.85866725578765	-10.87168132240831	-4.55254371496420
C	22.64242156135728	-15.84933342053936	-3.67220533803724
C	24.05796590247313	-16.44238529503888	-3.56911311170887
O	21.27772961261708	-11.27043907296137	-2.49134495247051
Si	20.21431737695561	-11.30979458707779	-1.13519499235522
C	21.32406899431650	-11.00176693733911	0.34444383292401
C	19.29618932427299	-12.93619917276488	-0.97410855188998
C	19.03996673087159	-9.85623030913741	-1.50368233310319
C	19.85665944981610	-8.60368033425743	-1.87545960746340
C	18.10848987918918	-10.22949287864266	-2.67337264236813
C	18.19706683849411	-9.56463887902300	-0.24560886785999
H	23.20071949272184	-13.97462148708142	-4.69435937949206
H	21.47041939312857	-13.86732069756414	-2.15179654159551
C	24.91499596804584	-10.57683475321857	-3.50913455793078
H	22.04354172280243	-16.13352078784563	-2.79049812202834
H	22.13493920580591	-16.28667102699117	-4.55169890242569
H	24.64423135649081	-16.13346840197093	-4.45335837024492
H	24.57013455231157	-16.00446585804957	-2.69409869500591
H	21.85640234643692	-10.04137621431470	0.25608371228044
H	20.73708955155134	-10.98869407886738	1.27763986309763
H	22.07430336909835	-11.80590423352445	0.42668983219053
H	18.93779001730925	-13.28328800268742	-1.95360573308329
H	19.92791180773351	-13.71976831257597	-0.52621483834453
H	18.42680797292972	-12.78976722816780	-0.31127324068914
H	20.45409673994117	-8.76655387503669	-2.78581025975503
H	19.17969235703680	-7.75026064359707	-2.06184004199563
H	20.54712323177495	-8.30682928645951	-1.06840722229479
H	18.67272012966665	-10.49937736140850	-3.57957665013340
H	17.46092407686544	-11.08539659487536	-2.42335860866105
H	17.45073522962270	-9.37709578089694	-2.92317818911446
H	17.48488314828323	-8.74416217842936	-0.44780258706611
H	17.60532650170229	-10.44005291084306	0.07050125406908
H	18.82251668485969	-9.25404030586124	0.60764336667298
C	22.67768781341345	-12.75891036284621	-7.58189790123857
H	23.62695115603272	-12.91547649652919	-7.04563381201858
C	21.63546431441155	-13.79482176775260	-7.13203428816879
H	22.07055458338098	-14.74822818945575	-6.79882908475955
H	20.90755646168397	-14.02299248559185	-7.93096016658420
N	20.92042302361729	-13.12838453730131	-6.03178993437293
C	21.23455873022784	-11.84770822452457	-5.94709498463259
H	20.56103042235799	-11.17412343367052	-5.41681296072749

B	19.65569825826257	-13.76635775826869	-5.38212353637548
F	19.91282010022988	-15.10023755214189	-5.06405351896351
F	18.60709491538566	-13.69969706204116	-6.31033520728251
F	19.32293749233616	-13.01986056882796	-4.23976634533903
H	22.88400128282736	-12.80765340818933	-8.66002394086873
C	22.04727691687083	-11.42839994625111	-7.14246582421862
H	22.75760585916039	-10.61393158023720	-6.96156377355391
H	21.32471805994482	-11.06109706571131	-7.89613866393171
C	24.05046392576592	-17.96649286898456	-3.45965257523833
H	24.86249385194323	-11.33100168476654	-2.70570719660829
C	24.74968796230572	-9.17485580624039	-2.89739397650723
H	25.91788139449300	-10.65664097753667	-3.96590724677973
H	23.74980693053032	-9.11420390470556	-2.43246968434954
H	24.75786992980192	-8.42124923615813	-3.70542234949587
C	25.82758488300873	-8.84250128284996	-1.86635511851664
H	24.06376120098077	-10.48807330098830	-5.56047546722969
H	26.83411513090351	-8.86755028013204	-2.31766947517856
H	25.81946410028740	-9.56757545646421	-1.03467478976857
H	25.67778184767419	-7.83847208269447	-1.43707166814925
H	23.56715844312754	-18.42508604592910	-4.33892552098779
H	23.49591542858221	-18.29911808003823	-2.56590768186786
H	25.07372637200637	-18.36931882254956	-3.38887689158623

Cycloadduct from SiOD + d-BF3 endo (FPDN)

Total Enthalpy ... -1566.72721737 Eh

Final Entropy Term (T*S) ... 0.09506523 Eh

Final Gibbs free energy ... -1566.82228260 Eh

C	1.41724277031126	0.17649200374252	-0.08972542561881
C	0.84298464120289	-2.52051288724719	0.01594151103741
C	0.13236921608619	-0.24131089591755	0.59131142831331
C	-0.15931761333744	-1.55370436584277	0.58271143158438
C	1.61619741927927	1.69216007184152	-0.19200893250263
C	0.29791807612124	-3.94985488777959	-0.01914504503624
C	0.20343528519263	-4.55528925478727	1.38592735286590
O	-0.64810033672926	0.74924168228718	1.05824230911486
Si	-2.31294865005693	0.77379084728780	1.46445980809220
C	-2.58063597644695	-0.22642032793804	3.03066592958227
C	-3.29142720931542	0.07524104638207	0.02202724591250
C	-2.64275227674847	2.63098106060531	1.73071009872959
C	-1.72635695619405	3.16393409121238	2.84919429271997
C	-2.35354494461773	3.39658943533248	0.42476498872046
C	-4.11680073303647	2.83491414155231	2.13289127039693
H	2.26927029861091	-0.23982141910659	0.47314420330311
H	1.73945590229966	-2.52276637287435	0.65338514923425
H	-1.09114670128335	-1.95016698196522	0.98686975704719
C	2.96962507333490	2.11206866017626	-0.76785303661384
H	0.80206096808805	2.14064173676245	-0.78609971238082
H	1.50906176981219	2.11408032519821	0.82045225866548
H	-0.70099000625041	-3.95696737336056	-0.48824182479034
H	0.95512794212833	-4.58408818892746	-0.62791014481929
H	1.21145592995477	-4.56256495849603	1.83790508140509
H	-0.41562890144700	-3.91214838013806	2.03538610599503
H	-1.91128735488666	0.11545383046867	3.83648330654887
H	-3.62041679599005	-0.11803799121192	3.38156395666615

H	-2.39272159849286	-1.29847078916480	2.86136337269614
H	-3.04204698355194	0.59721855849058	-0.91550769937708
H	-3.08979816994716	-0.99769624425398	-0.11901322308032
H	-4.37241904761553	0.19502752008649	0.20354807479988
H	-0.66149191087723	3.03200016834678	2.59925364881433
H	-1.90311624328379	4.24383535974848	3.00509062524643
H	-1.91310020510689	2.65756274031323	3.81050257425649
H	-1.30911375777201	3.26601266424622	0.09993419574717
H	-3.00650725713025	3.06532191086175	-0.39934056419167
H	-2.52735825387745	4.47851371039265	0.56912058103984
H	-4.32398428371543	3.90923826633037	2.28886122790815
H	-4.81187547887255	2.47625729356220	1.35548124089836
H	-4.36402273194915	2.31331652396365	3.07232978873657
C	-0.36941731288296	-5.97215132240925	1.37457622201481
H	3.06791184816096	1.76703812699105	-1.81283578412194
H	3.77925206434801	1.60885484460259	-0.20709435513056
C	3.18750918250590	3.62663028550274	-0.72675195488765
H	2.37000422164727	4.12376079725392	-1.28089815030690
C	4.53605744020756	4.06057367909496	-1.30053985902535
H	3.09793555912443	3.97564191460430	0.31842525435246
C	-0.50337753335473	-1.48521058211098	-2.67816488114017
H	-0.93814024839271	-1.50513296721754	-3.68826762977318
C	0.62217203219535	-2.49677942400991	-2.53812724901108
H	1.29839350336771	-2.44092722940530	-3.40131729799418
H	0.30495555919039	-3.53620189499760	-2.41840594879677
N	1.41963952917566	-2.04141460866020	-1.34668159794535
C	1.41086851681522	-0.51253015810646	-1.46613231369125
H	2.33328407901862	-0.26756937243845	-2.00033504180472
H	-1.30738717127448	-1.69871432102360	-1.95805783316296
C	0.18526075555872	-0.15350898695567	-2.34475236883716
H	0.52655135901830	0.35059639837264	-3.26093409751301
H	-0.49579422132797	0.53574461643557	-1.82674154492210
B	2.96146420868563	-2.55818690050205	-1.48023390701071
F	2.95985322826309	-3.94508910354622	-1.44952591888036
F	3.67678858781883	-2.02372042583208	-0.41466106254842
F	3.45784565635878	-2.09226932614940	-2.69286705535547
H	4.63811500992913	3.75335186093050	-2.35553170081699
H	5.37152655979351	3.60398799459546	-0.74269824882554
H	4.66312839467418	5.15460869284197	-1.25697541524026
H	0.24587842396943	-6.64543181217751	0.75399403382960
H	-1.39241585892622	-5.98461561189688	0.96162524634539
H	-0.41302828755752	-6.39710749396676	2.39031325253253

Cycloadduct from SiOD + d-BF3 exo (FPDX)

Total Enthalpy ... -1566.72753011 Eh

Final Entropy Term (T*S) ... 0.09417008 Eh

Final Gibbs free energy ... -1566.82170019 Eh

C	1.78071916603713	-0.17044652171375	0.08352340811273
C	0.94001583401138	-2.75783608176865	-0.00226140893327
C	0.35730654351985	-0.45252091134771	0.51656851816895
C	-0.04061659471074	-1.73464351182840	0.48356020873586
C	2.15357949335569	1.32017982902906	0.03309723426266
C	0.35948765255220	-4.17270860948935	-0.01783028702694
C	0.14740010046856	-4.70861056372649	1.40169835521066

O	-0.38425005991559	0.60984958536989	0.88249991978529
Si	-2.05808687484788	0.71924705652993	1.23954256855860
C	-2.39021263775099	-0.20611040221820	2.84006099078266
C	-3.03633895555319	0.00440669102778	-0.19544251927922
C	-2.34042193578121	2.59423150829603	1.43434001105827
C	-1.30483876435417	3.18199475574251	2.41179820212680
C	-2.20626142771429	3.28907700289837	0.06581495719626
C	-3.76056014743660	2.82862125916420	1.98738591726313
H	2.43598045571658	-0.64424394433943	0.83817514199168
H	1.82148235850148	-2.77333852295497	0.66506545572184
H	-1.05318140487083	-2.05108519777063	0.73167306464853
C	1.52658432816797	2.12054020814548	-1.11491576891821
H	1.84989101489369	1.76959666819994	0.99198260334946
H	3.25148255056540	1.40114645572801	-0.00960343557279
H	-0.59498183266845	-4.17651174076742	-0.56539466440591
H	1.03213517101413	-4.85595831920749	-0.55742464266156
H	1.11540849418325	-4.71369676128855	1.93505214287050
H	-0.50584498599810	-4.02119874901567	1.96691844380816
H	-1.81852128369476	0.22891796826204	3.67584656923816
H	-3.46150634923772	-0.15799796793986	3.09639457445664
H	-2.11262952444973	-1.26816063449801	2.75096260882270
H	-3.03894688198572	-1.09605647359669	-0.18134618381375
H	-4.08315761424034	0.34762185579988	-0.14825977928985
H	-2.60955318332289	0.32699362744202	-1.15834304320146
H	-0.27685400688554	3.05138253309279	2.03948346729122
H	-1.47926737692494	4.26520560167799	2.54643864107138
H	-1.36458287780789	2.71281656447846	3.40800851586123
H	-1.21154126031978	3.13114948889332	-0.37798810599431
H	-2.95811988313325	2.92721240348192	-0.65436220056864
H	-2.35184360657234	4.37931807883037	0.17578656544667
H	-3.95914458939604	3.91206100334469	2.07796232431495
H	-4.53696659338415	2.40794280151545	1.32630018011612
H	-3.89322379065244	2.38503352215098	2.98776048054713
C	-0.45729337807169	-6.11229292979025	1.41320649739733
H	0.46016330103188	1.85718053497624	-1.19781855539070
H	1.99170960705334	1.83459112949572	-2.07527463747102
C	1.65199390958433	3.63886152404199	-0.94143539913053
H	1.15706305426054	3.93459178575221	0.00157650950070
H	1.08425358337411	4.13448774570252	-1.74852753188837
C	3.09169331285588	4.15806802533305	-0.94648852237058
C	3.73785014787245	-2.24623999807856	-2.37499157844658
H	4.76030803609250	-2.64968766918827	-2.32975868613251
C	2.74518846368840	-3.17501943233536	-1.69042909145408
H	3.16579236560367	-3.55722148376820	-0.74791124393953
H	2.42245052803334	-4.02595027063702	-2.29953217061438
N	1.54531211605092	-2.31810000026910	-1.34669000343035
C	2.07367587391038	-0.86701161057363	-1.25980754273182
H	1.55093496224765	-0.32283346051978	-2.05090256554101
H	3.46778715123217	-2.10202732831211	-3.42948644885328
C	3.57156809957853	-0.95006605068192	-1.58280719528500
H	4.14693251979660	-1.00765856168300	-0.64352706215521
H	3.91173942799451	-0.05894006242839	-2.12935055194186
B	0.47986431462069	-2.39571176969599	-2.59447568382903
F	1.12203887319619	-1.84987925019229	-3.70407716864352
F	0.17305833684230	-3.72913301406306	-2.82549565679018

F	-0.64762001703701	-1.66081905112348	-2.27359897671062
H	3.61374678218790	3.87187464237637	-1.87610862699637
H	3.67583405226949	3.75613631310628	-0.10249193921828
H	3.12113933178722	5.25740966361002	-0.87222824164300
H	0.18945901364320	-6.82998426851015	0.88039486130955
H	-1.44225229494804	-6.12392009818412	0.91663829786193
H	-0.59528019412921	-6.48373660998954	2.44144988338599

Cycloadduct from SiOVA + d-BF3 endo (FPVAN)
Total Enthalpy ... -1565.52979612 Eh
Final Entropy Term (T*S) ... 0.09324131 Eh
Final Gibbs free energy ... -1565.62303743 Eh

C	1.19268757881592	1.08877270536497	0.01954230426109
C	0.38086720555062	0.07790406352083	-0.68764829346846
C	0.88489091680384	-1.16993327558503	-0.73915617785422
C	2.31511001250204	-1.43097671411331	-0.34130499960529
N	2.72938797393838	-0.69878128359111	0.96095548658323
C	1.83832291721770	0.52098227592520	1.25797952029374
C	2.59665718361658	-1.53748382717310	2.20289852570842
C	1.36668417674149	2.37446510384355	-0.33364555726804
H	0.31253713778510	-1.97846072095825	-1.19779625749011
C	0.83119944232977	3.06777210093192	-1.54807118141023
H	2.01873854600424	2.98168418684395	0.30765947193365
C	1.95692285521670	3.67729696236821	-2.40059910388285
H	0.15083828572551	3.88224570313235	-1.23265617081420
H	0.23252866382925	2.37811759853164	-2.15954657461745
H	2.55073272841488	4.37124061305350	-1.77851857562709
C	1.43081867301201	4.40775884180578	-3.63520872555370
H	2.65008293356236	2.87265758067519	-2.70374183233257
C	2.65995842694840	-2.92182620076436	-0.34118089462498
H	2.95743173453609	-0.94805353561382	-1.09149208017505
C	2.70557245218242	-3.50139826396597	-1.75956448963397
H	1.92466683853599	-3.48204987435616	0.26154580132498
H	3.64677962954426	-3.06715650854905	0.11806165008134
H	1.73778422881610	-3.34892791746058	-2.26749014936175
C	3.05589610145004	-4.98893422231044	-1.76935345604656
H	3.45071678687244	-2.93764418440937	-2.34897809543895
O	-0.80275523337399	0.40498383732582	-1.25216007452783
Si	-2.29266518873918	1.01417738990784	-0.67923602123796
C	-3.32936238907387	-0.49627386158219	-0.13586141205501
C	-3.02177141278832	1.86215954595694	-2.18217320751130
C	-2.05424154625440	2.24056121542153	0.72278911018014
H	-1.53813299169343	1.80518507936182	1.59066728266031
H	-3.04174067248046	2.59784358621978	1.06024840421774
H	-1.47640388499530	3.11386967977561	0.38327497157175
C	-3.43722540404898	-1.48780311114725	-1.31020184731096
C	-4.73939456218497	-0.03135126898971	0.27743009570604
C	-2.64913094490362	-1.19498702824552	1.05715987481065
H	-3.07816909864837	1.17426783780304	-3.04080807800746
H	-2.40179282330733	2.72573615736419	-2.47314285231912
H	-4.03884089392634	2.22957639686396	-1.96865573830656
H	-5.27050668530802	0.46472509275742	-0.55178883375038
H	-4.71015670715242	0.67078346600496	1.12712230425960
H	-5.35156447610175	-0.89762348626298	0.58884993961577

H	-1.63692777385181	-1.54746369538729	0.80287808303076
H	-3.23832190050864	-2.07652314270111	1.37012170313332
H	-2.56561095213057	-0.52973072486763	1.93212249534881
H	-3.95519226812233	-1.04489922931911	-2.17682933370795
H	-4.01040843444543	-2.38239147325231	-1.00501656868470
H	-2.44459473728319	-1.82581624238454	-1.64870683237304
C	0.81678013855414	0.04133788828262	2.32778268837478
H	2.50855900414102	1.26592890277864	1.69717653772579
H	-0.21562292795878	0.17998060803270	1.98669320965368
H	0.94585084768655	0.64260130705267	3.23972007387406
C	1.13304921673036	-1.43527561997447	2.59258539057011
H	3.23199467632235	-1.06345129854606	2.96286996165515
H	2.96764139996675	-2.55070934966067	2.03034426854905
H	0.51642050988728	-2.08835945817926	1.95561882399322
H	0.96976222149869	-1.73149244903692	3.63911161123566
B	4.27109400733246	-0.18842673693510	0.79495938760343
F	4.32047455882245	0.64410823861562	-0.31400547310779
F	4.61183234236368	0.50162491006544	1.95266198499024
F	5.07173570104015	-1.31005339737099	0.63226552298993
H	2.25306999560924	4.83274139358000	-4.23336534592856
H	0.75880454413213	5.23559944505699	-3.35120139892373
H	0.85821346562334	3.72618567951398	-4.28701325122131
H	3.09824842379452	-5.38505516620872	-2.79671248369928
H	2.30715862047704	-5.57842048461257	-1.21336131593388
H	4.03661080534720	-5.17079164022374	-1.29803380212411

Cycloadduct from SiOVA + d-BF3 exo (FPVAX)

Total Enthalpy	...	-1565.53008936 Eh
Final Entropy Term (T*S)	...	0.09352012 Eh
Final Gibbs free energy	...	-1565.62360948 Eh

C	22.31473816667910	-11.64488944136064	-4.75078029909069
C	22.48734238622881	-14.31206646059519	-4.44462517971869
C	21.89705293181202	-12.17703590524968	-3.43094470326589
C	21.93690274152926	-13.51326636519589	-3.29792255377037
C	23.16137215983160	-10.61510578460011	-4.91391940040575
C	22.48409062782787	-15.81384984365352	-4.15284234879305
C	23.53104143668951	-16.18231380820156	-3.09655101823882
O	21.44259066211620	-11.28540274699071	-2.52397353501044
Si	20.30050946868837	-11.52462076661866	-1.26386367226944
C	21.06723566522711	-12.58920507631485	0.08194339264251
C	18.77967798529021	-12.33401227214843	-2.00561238431693
C	19.96637329054492	-9.75133919193993	-0.65363796972395
C	21.26559109405992	-9.14698321219903	-0.08707457062753
C	19.46350808454095	-8.88468097886486	-1.82351506975936
C	18.89225924570751	-9.79486726714905	0.45146270671596
H	23.53299866501616	-14.00830697306385	-4.62547735072134
H	21.57702495492137	-14.03643752012899	-2.41237937234018
C	23.83793159842954	-9.83658000556182	-3.82486031134153
H	21.48349812324305	-16.11870367520365	-3.80955983335605
H	22.68650463641844	-16.38572408190704	-5.06973630731600
H	24.53092340405259	-15.88352281869276	-3.46047180333090
H	23.35764423193056	-15.59827404159318	-2.17597862348204
H	22.07558011384065	-12.23073233963244	0.34483599831210
H	20.44607114004403	-12.55914135932458	0.99264087547181

H	21.14751136231319	-13.64200927227471	-0.23169594952212
H	18.27393196772623	-11.65110907975235	-2.70694667519316
H	19.04708354273576	-13.24647417013332	-2.55960110698167
H	18.06281207101736	-12.60598700370902	-1.21335330892936
H	22.06478737383230	-9.11859733208895	-0.84421069190831
H	21.09001707427921	-8.10973779127735	0.25243120259414
H	21.64009833808771	-9.71833010424758	0.77808294651109
H	20.19711024266784	-8.84425153131189	-2.64394292865370
H	18.51585813507988	-9.26454294320129	-2.23933860472384
H	19.28263816205535	-7.84903254880600	-1.48227052649992
H	18.68298342189073	-8.77438026388560	0.82078232913833
H	17.94028830087629	-10.21341699697786	0.08455611550129
H	19.21370795738540	-10.39720858203001	1.31731208212867
C	23.22806273328231	-13.07746414868032	-7.52317492422857
H	24.12691012106300	-12.77204260471268	-6.96414191759956
C	22.63575572898289	-14.35515251278711	-6.94508634152024
H	23.38173343387287	-15.08643113577529	-6.61357283155214
H	21.98177443553982	-14.84490549844159	-7.67552626379623
N	21.78283872048493	-13.92984705537691	-5.75952333173877
C	21.61882419592980	-12.38565298996425	-5.88570644305524
H	20.54567771908292	-12.18814843198227	-5.77548398058524
B	20.30351605848983	-14.60642461603723	-5.91358153493667
F	20.46537529970319	-15.96422774136981	-6.14298085146653
F	19.70130895413327	-14.00147066497056	-7.01338254009199
F	19.58638881195607	-14.36276276177271	-4.75203265264204
H	23.50780201821010	-13.20330449772596	-8.57935940695920
C	22.09327658694097	-12.07983814832032	-7.30630996875981
H	22.38442799731684	-11.03044833984272	-7.43722370543015
H	21.27126278094966	-12.28876392313782	-8.00580146715789
C	23.52561289279945	-17.67407175400603	-2.76441043870234
H	23.71925570324024	-10.33590720997484	-2.85189499786934
C	23.32949824369290	-8.38829369651690	-3.72423672135825
H	24.92167697545302	-9.80919244866202	-4.04523478729193
H	22.25279392852197	-8.40758194995198	-3.48491643213919
H	23.41534691222482	-7.90218160422827	-4.71277351682038
C	24.08079868193243	-7.56687768163275	-2.67769817623886
H	23.39423943976030	-10.28271160471593	-5.93092079105369
H	25.15708224521851	-7.50854103349557	-2.91312507867862
H	23.98510728431839	-8.01668051064950	-1.67527990804512
H	23.69285815242477	-6.53693409943736	-2.62024463118248
H	23.72649489852681	-18.28291841483810	-3.66226192810477
H	22.54662740414981	-17.98848244141309	-2.36483256590443
H	24.29108087717829	-17.92249092369210	-2.01163341481477

NMR spectra of the synthesized compounds

1H-NMR

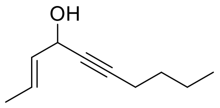

5

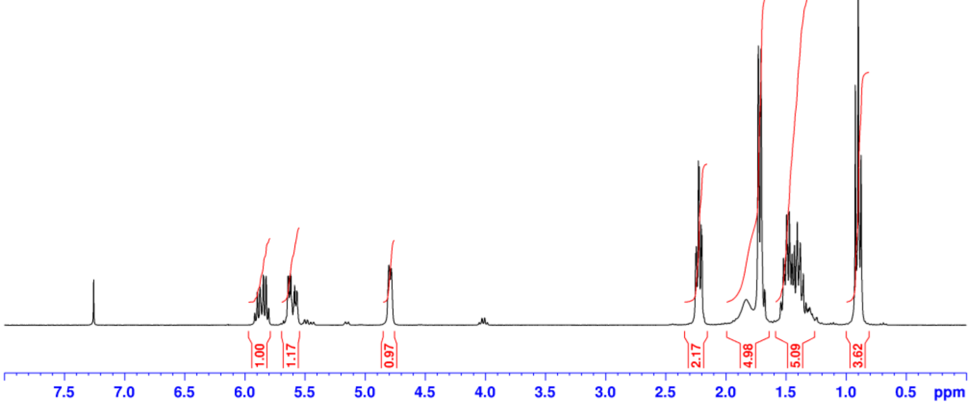

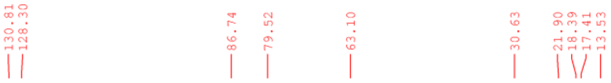

13C-NMR

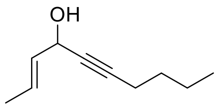

5

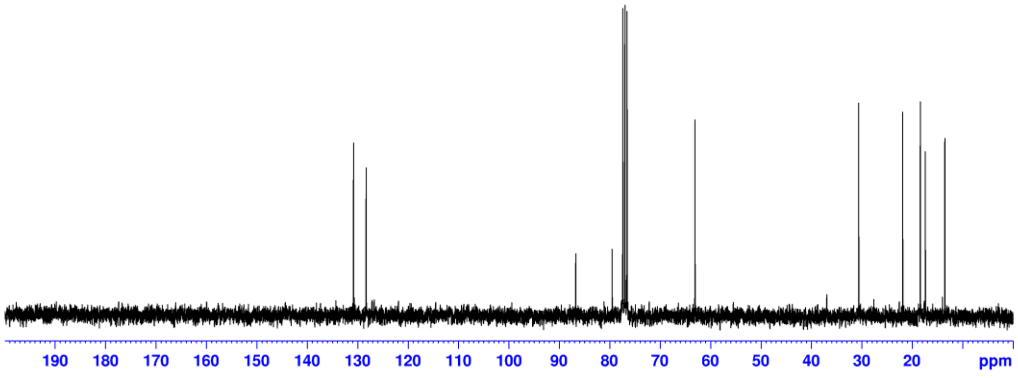

1H-NMR

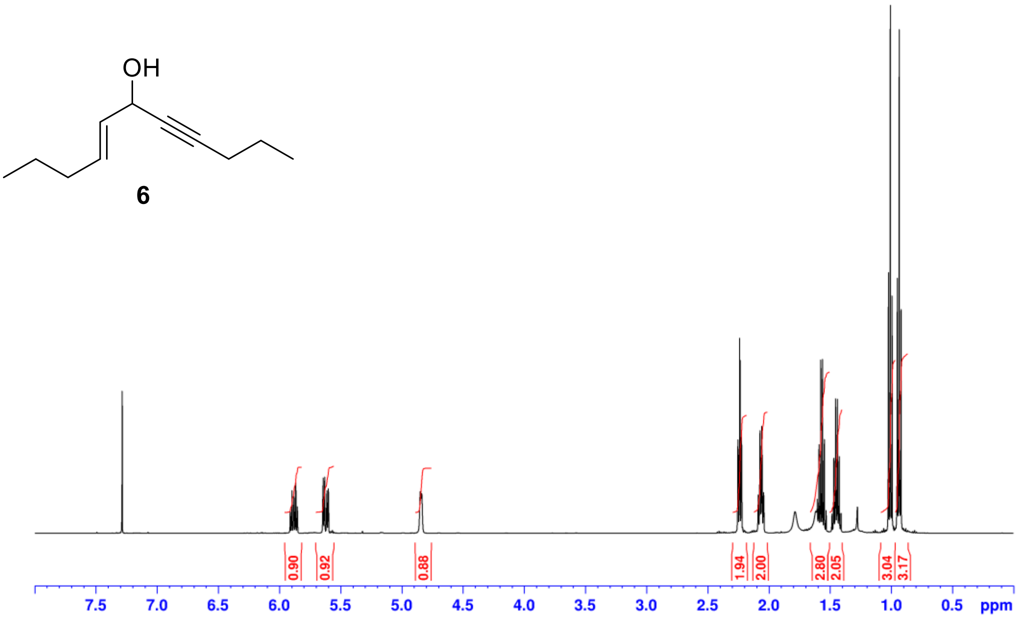

13C-NMR

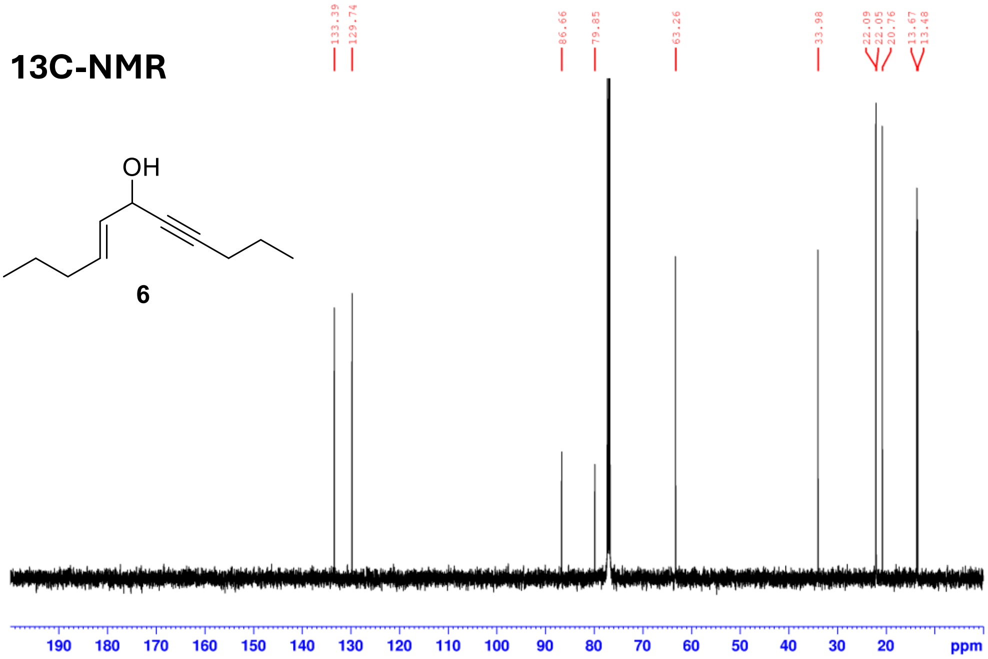

1H-NMR

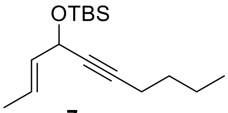

7

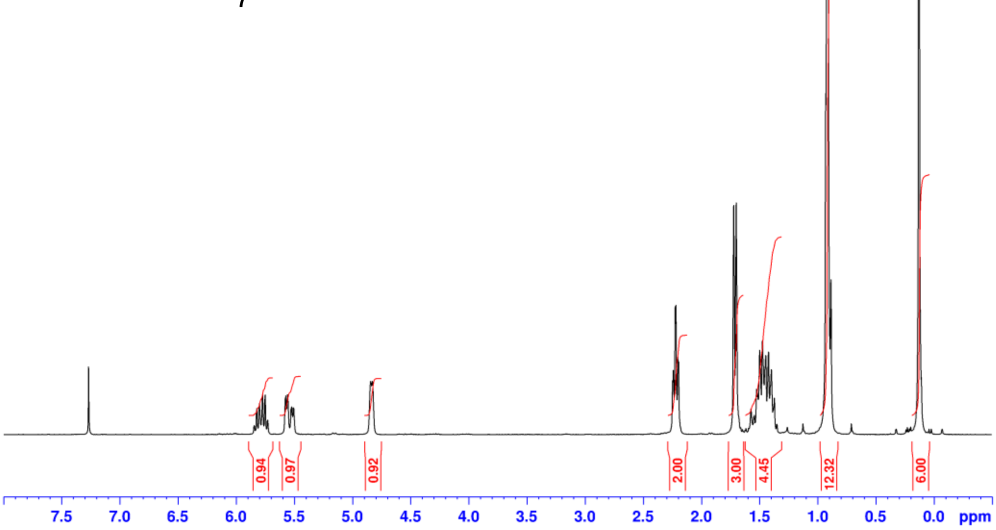

13C-NMR

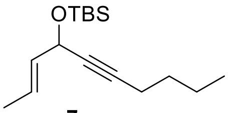

7

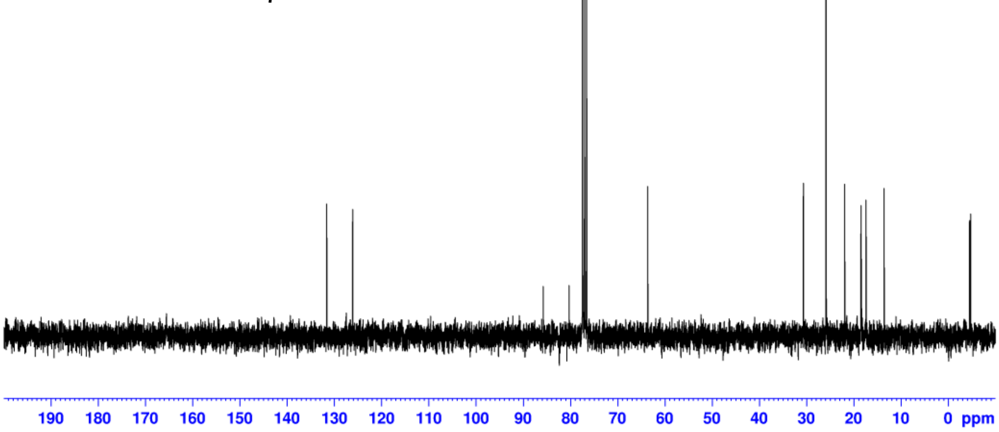

1H-NMR

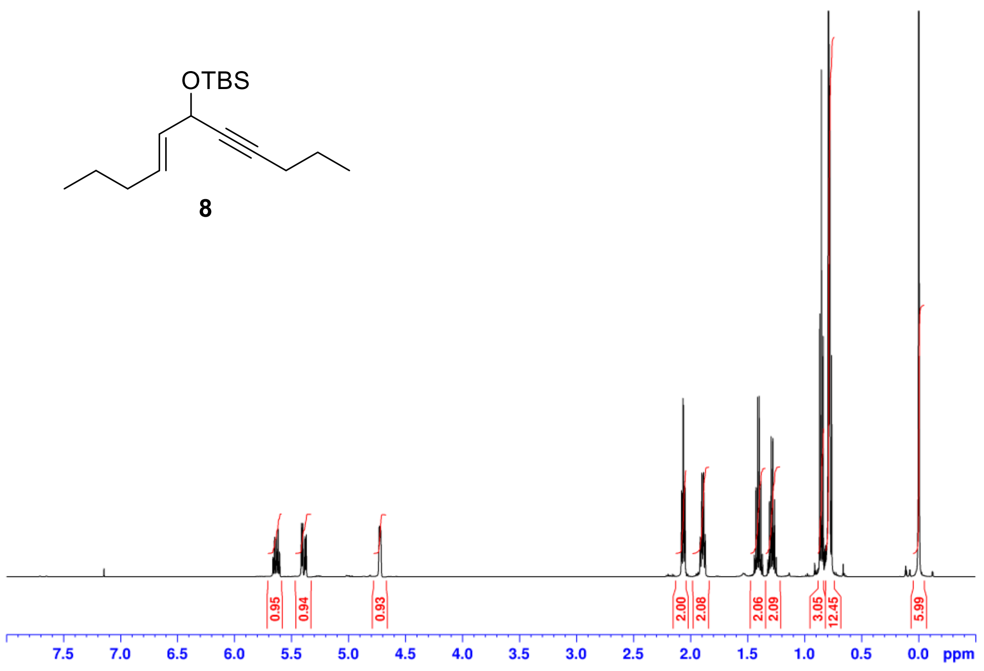

13C-NMR

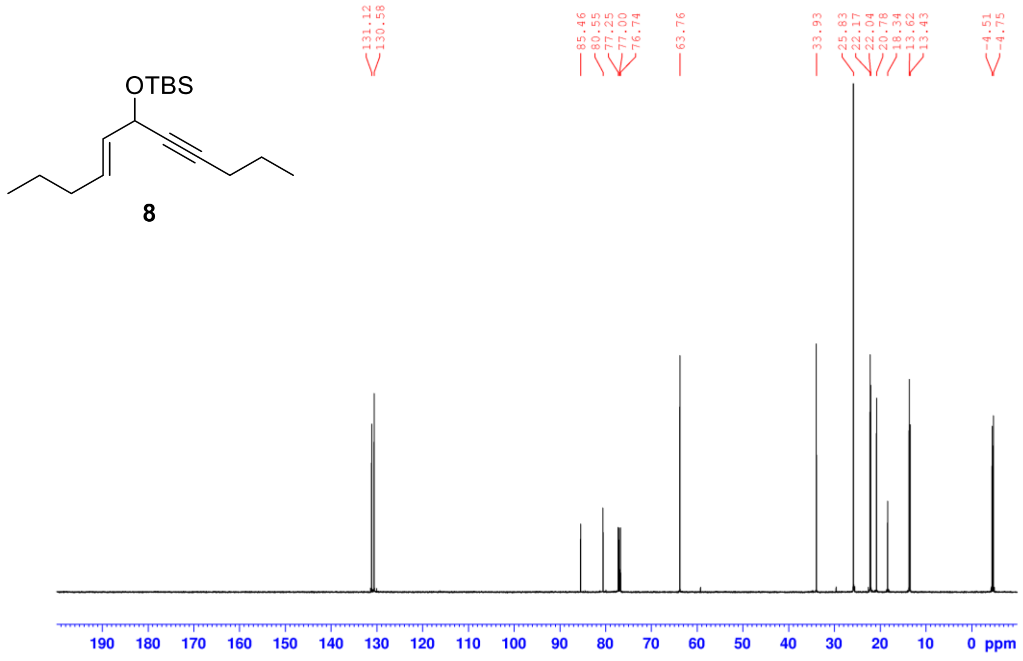

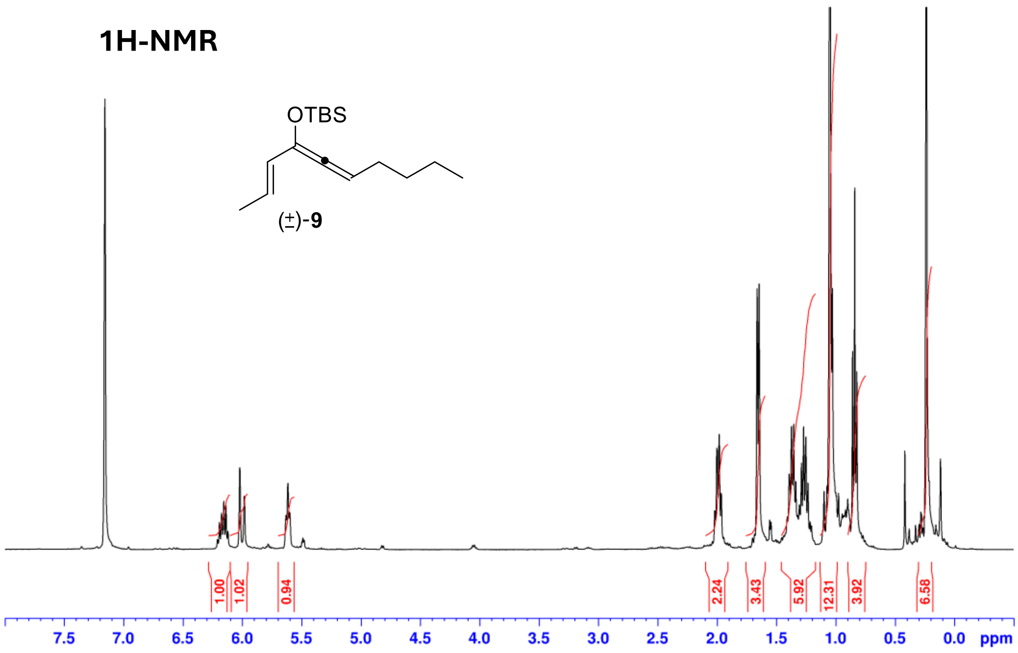
1H-NMR
OTBS
(±)-9
1.00
1.02
0.94
2.24
3.43
5.92
12.31
3.92
6.58
7.5
7.0
6.5
6.0
5.5
5.0
4.5
4.0
3.5
3.0
2.5
2.0
1.5
1.0
0.5
0.0
ppm

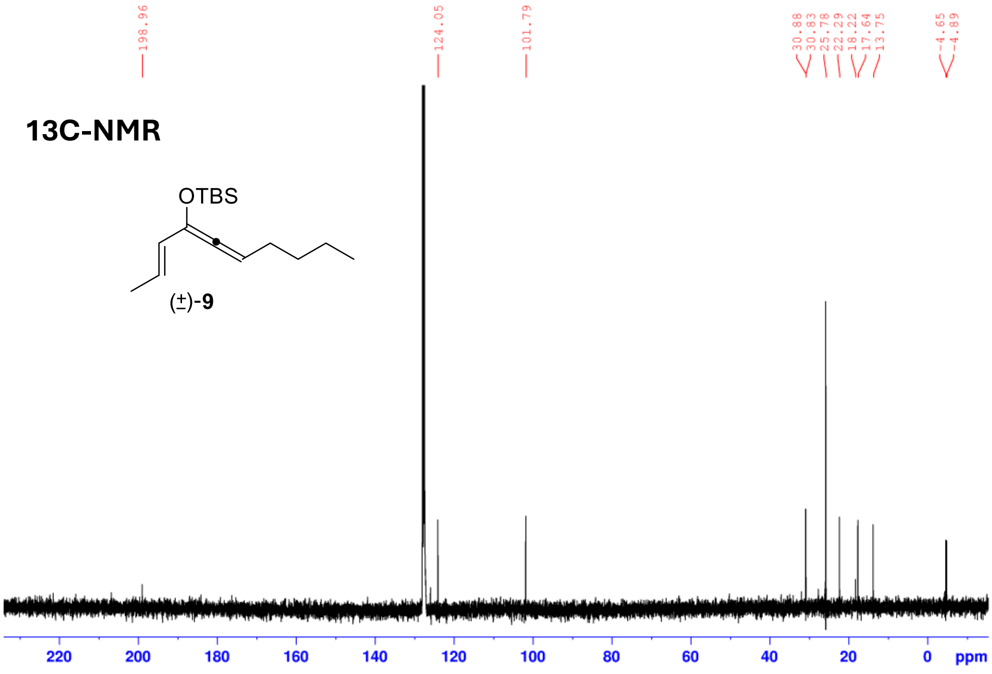
198.96
124.05
101.79
30.88
30.83
25.78
22.29
18.22
17.64
13.75
-4.65
-4.89
13C-NMR
OTBS
(±)-9
220
200
180
160
140
120
100
80
60
40
20
0
ppm

1H-NMR

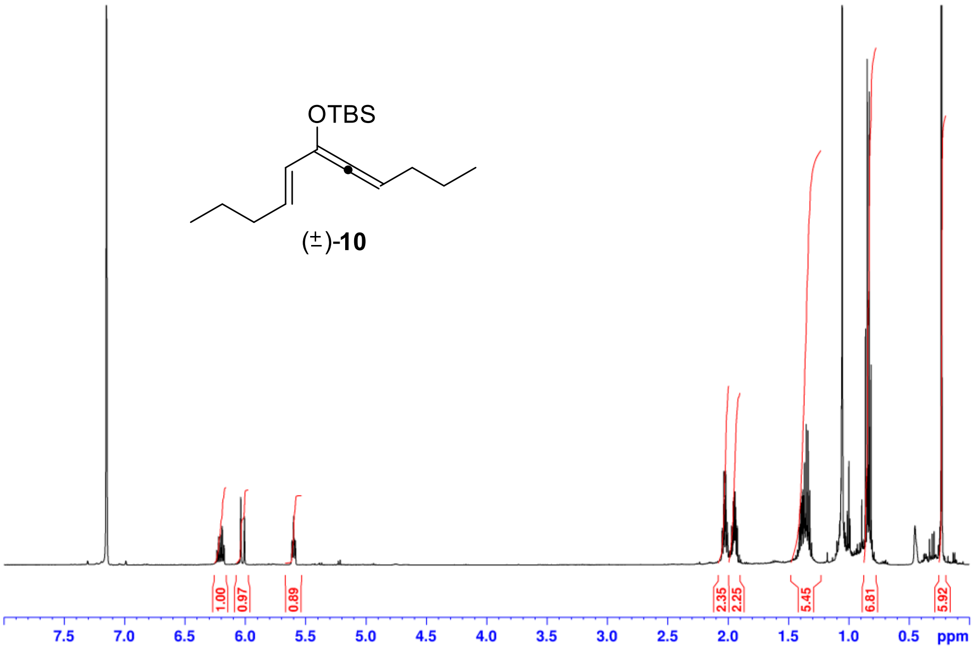

13C-NMR

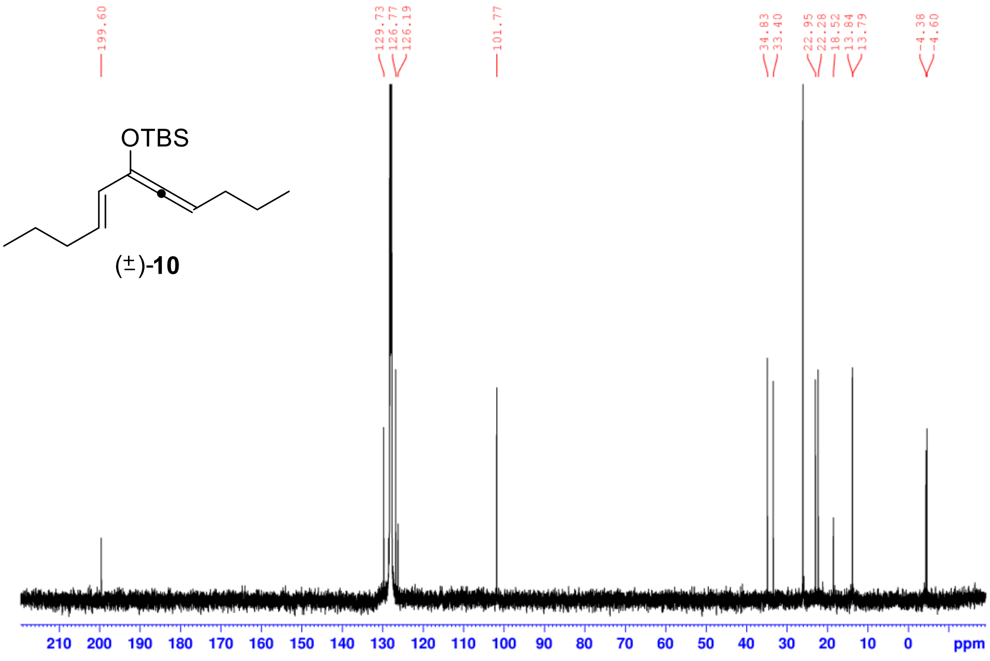

1H-NMR

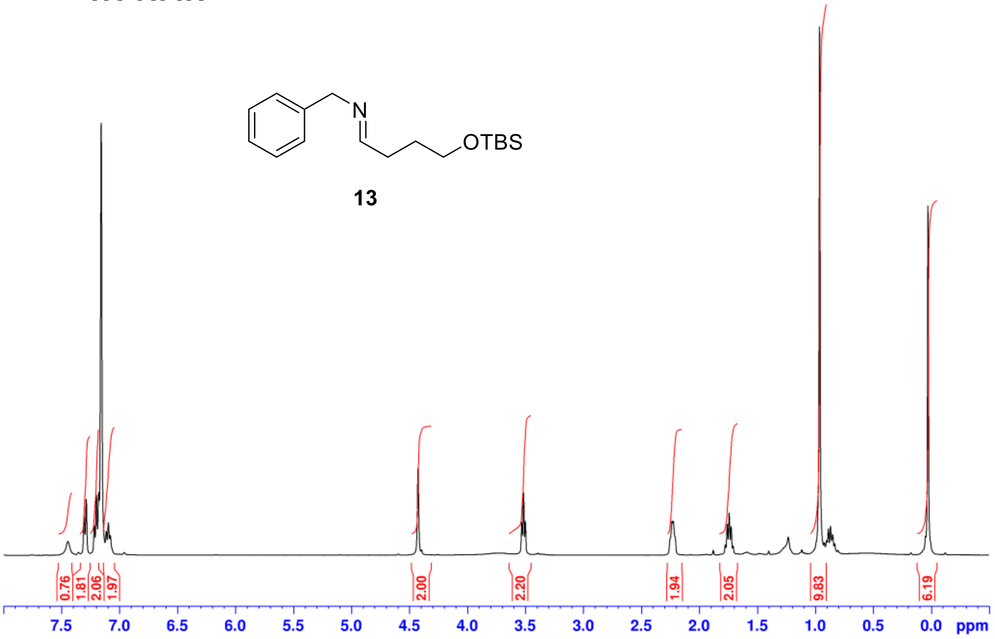

13C-NMR

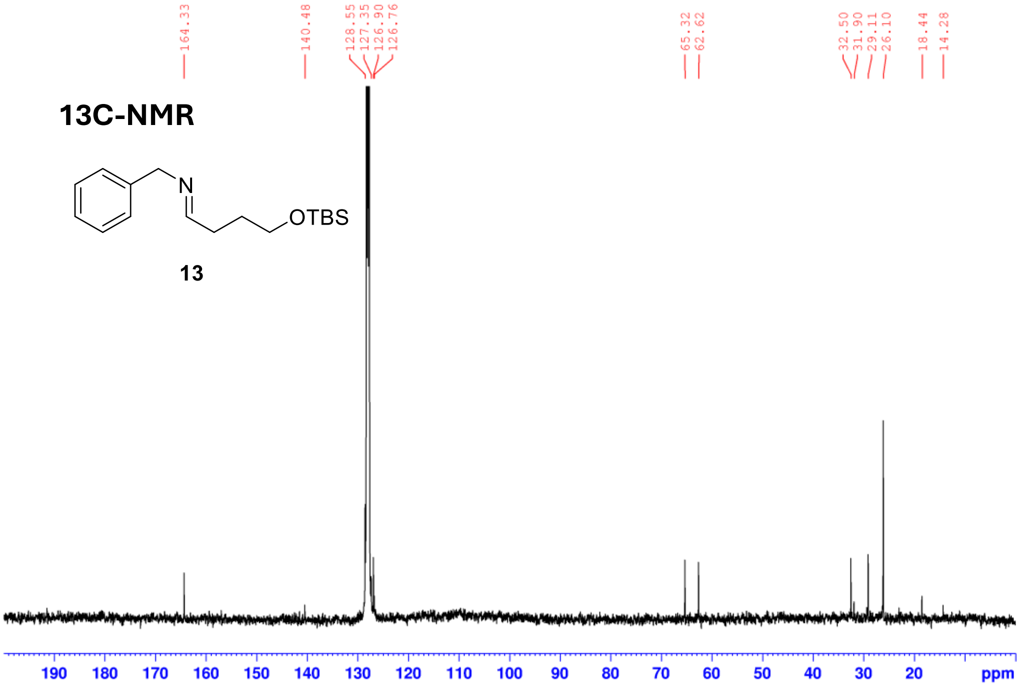

1H-NMR

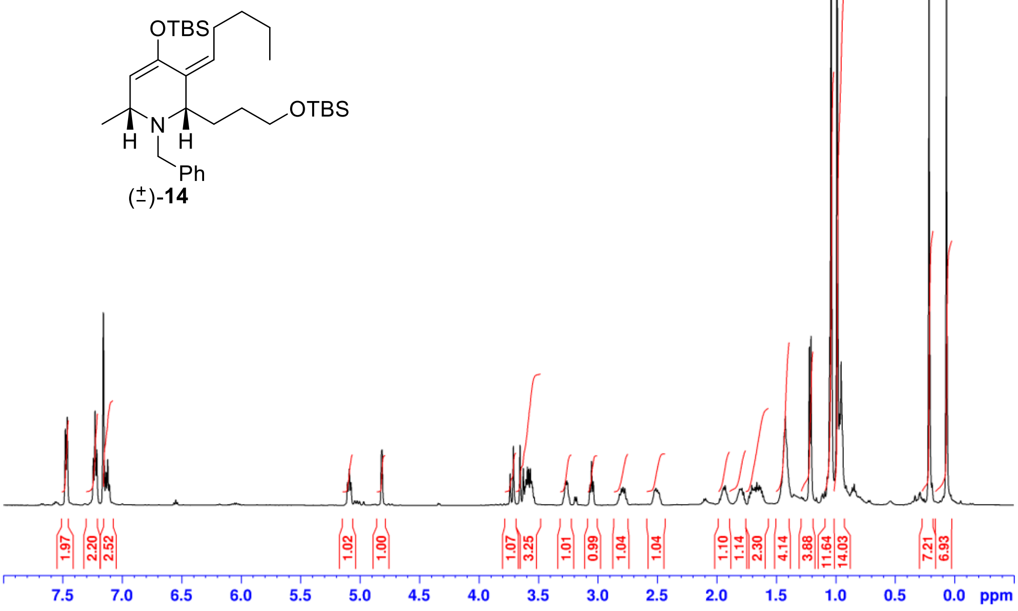

13C-NMR

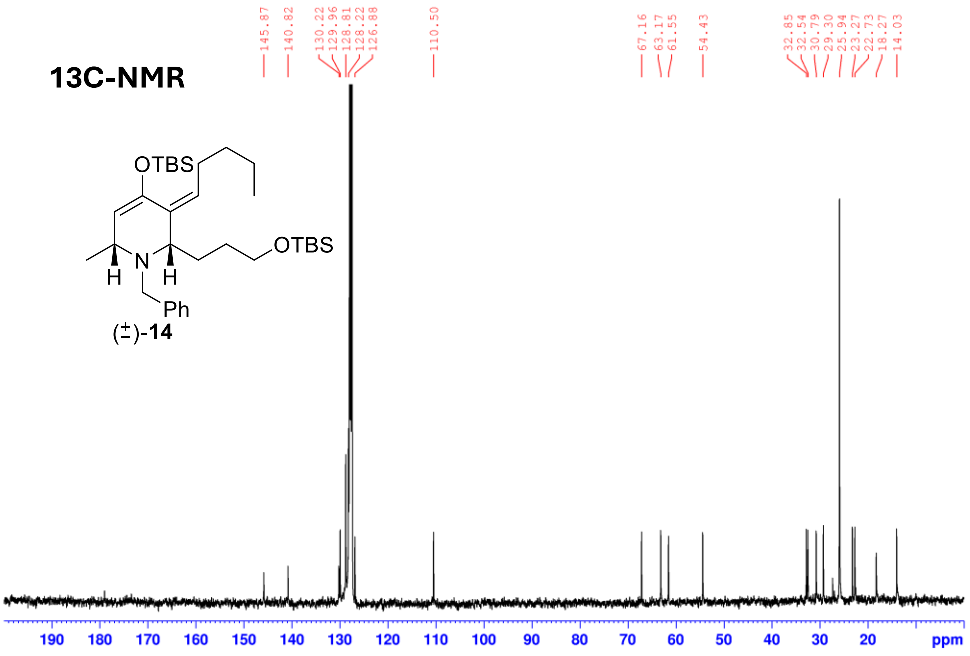

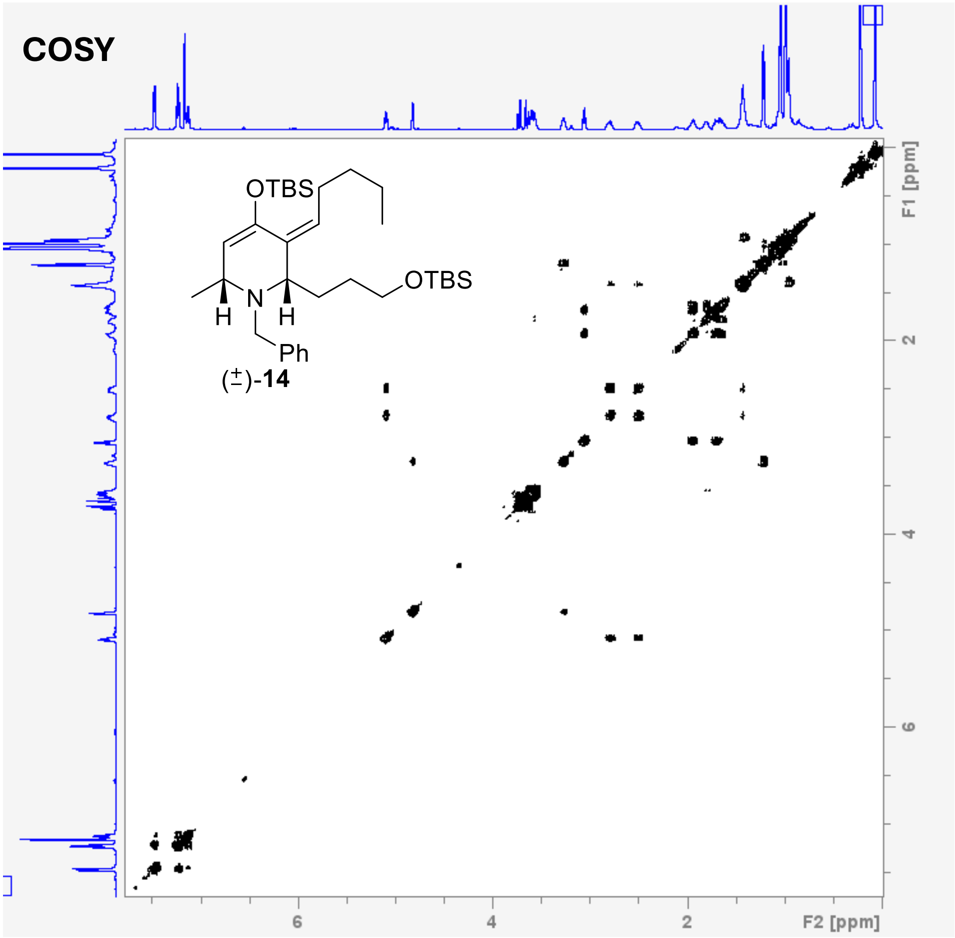
COSY
OTBS
OTBS
N
H
H
Ph
(±)-14
F1 [ppm]
F2 [ppm]
2
4
6

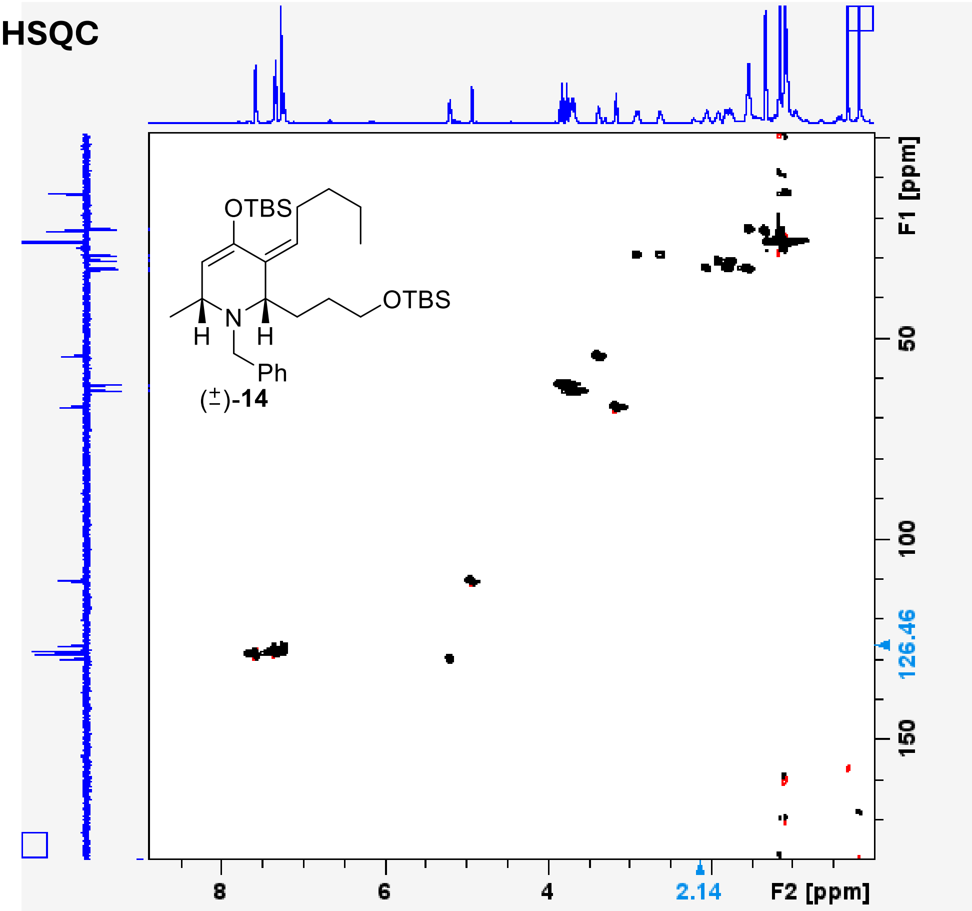
HSQC
OTBS
OTBS
N
H
H
Ph
(±)-14
F1 [ppm]
F2 [ppm]
50
100
126.46
150
8
6
4
2.14

1H-NMR

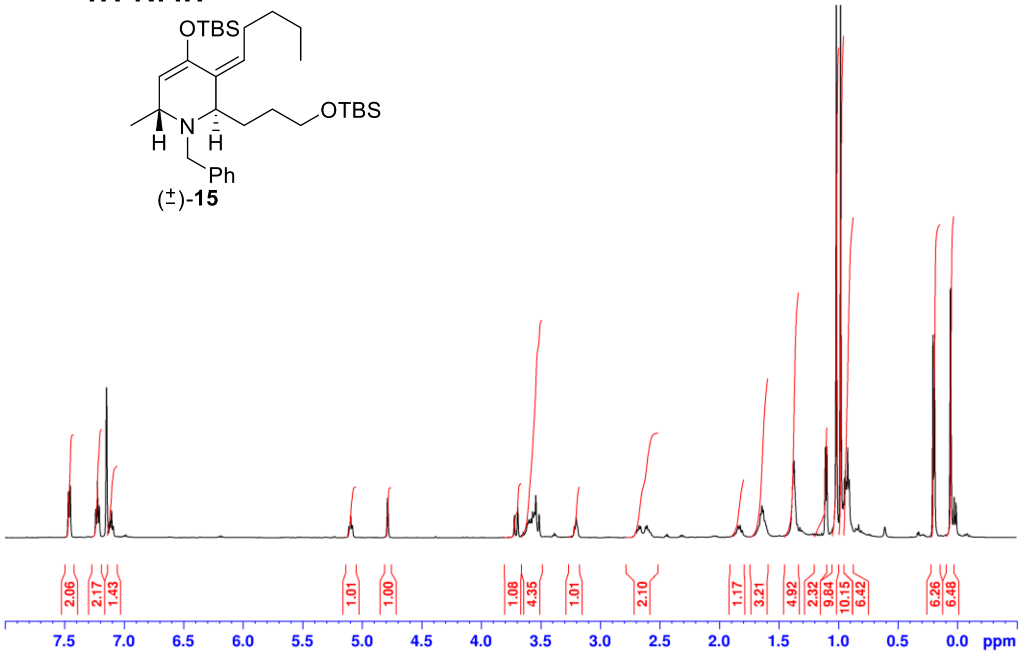

13C-NMR

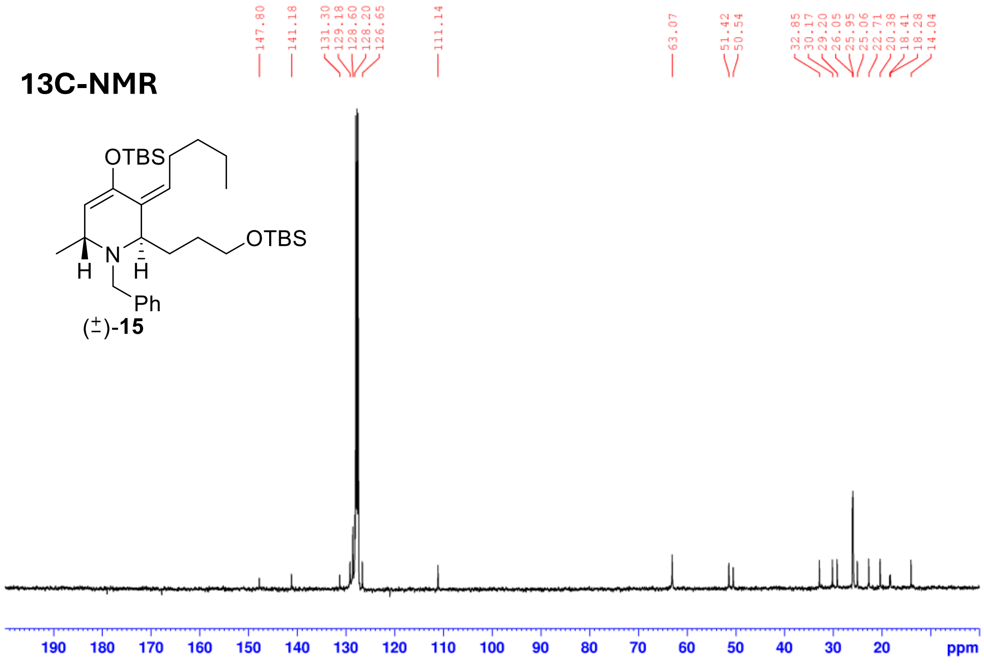

1H-NMR

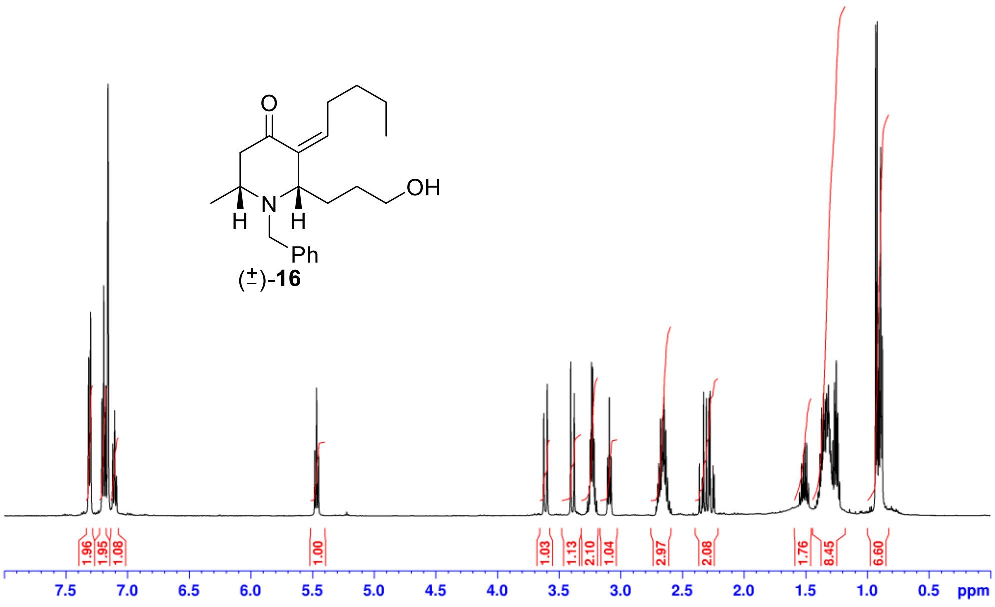

13C-NMR

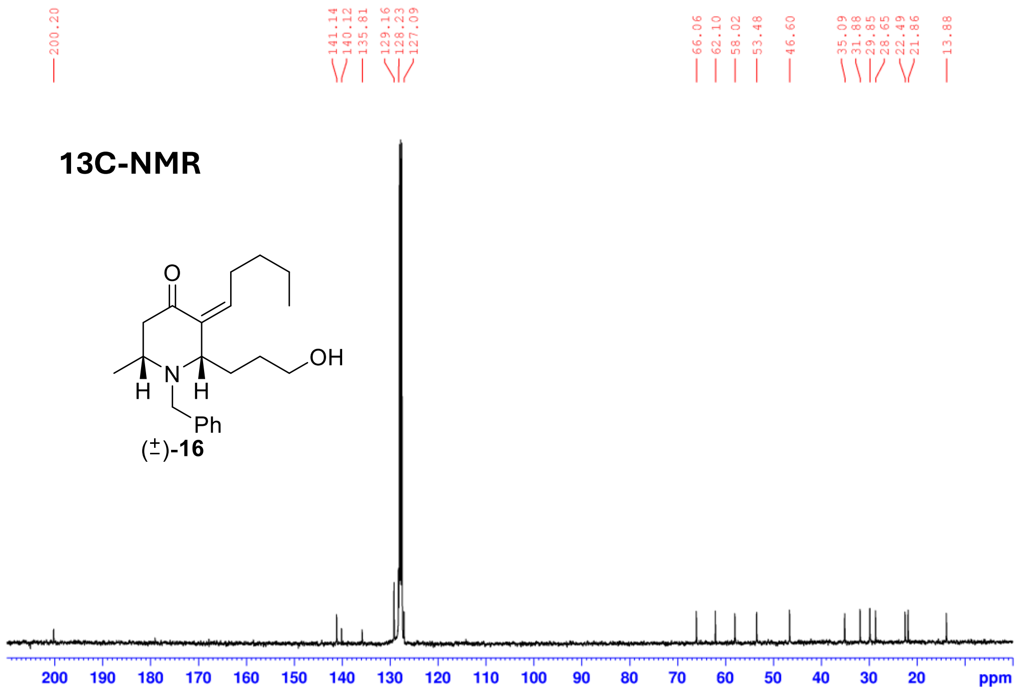

1H-NMR

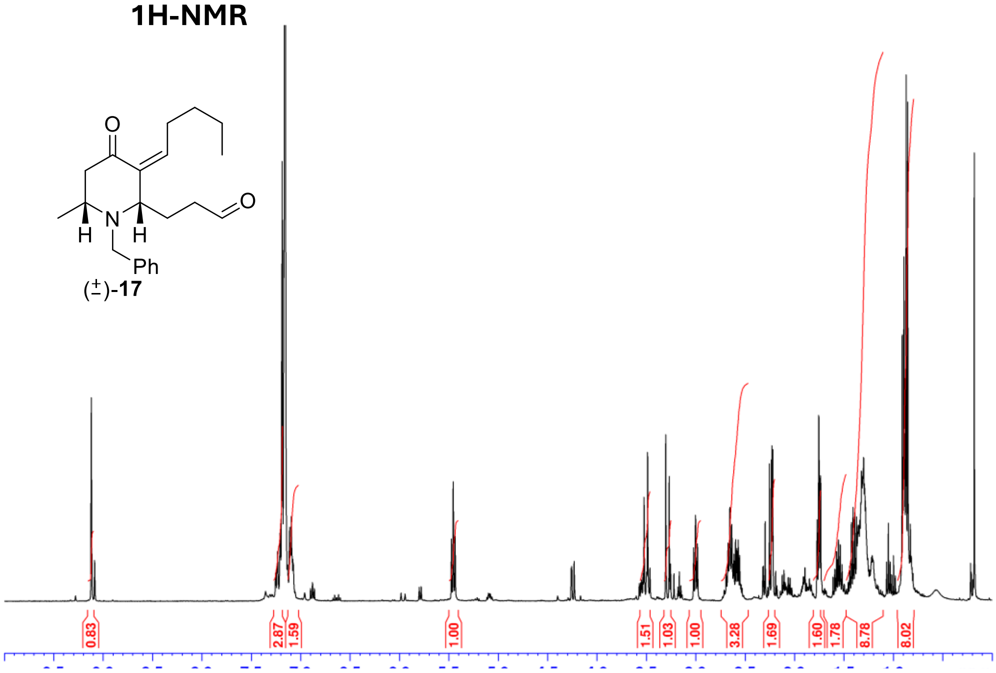

13C-NMR

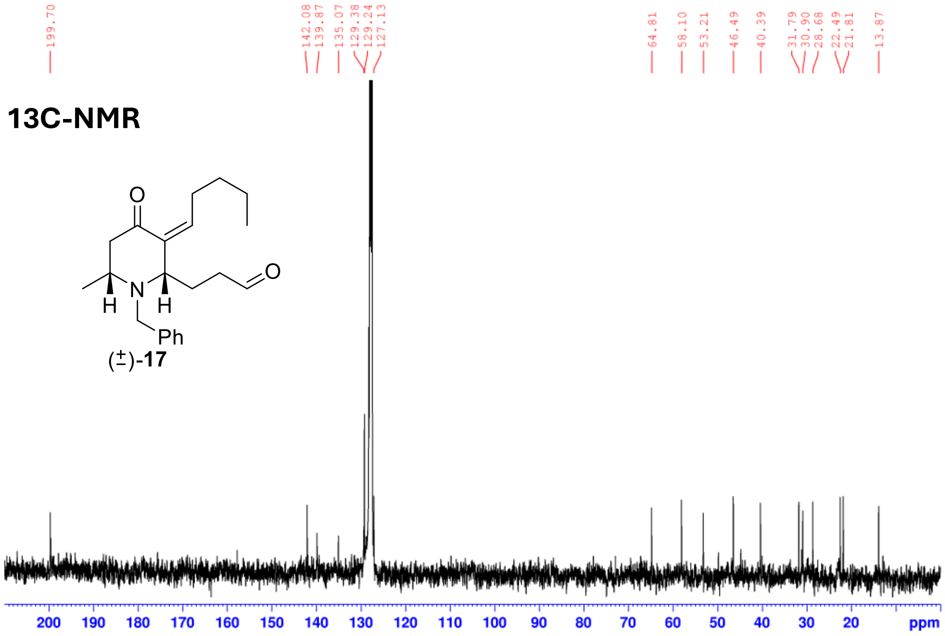

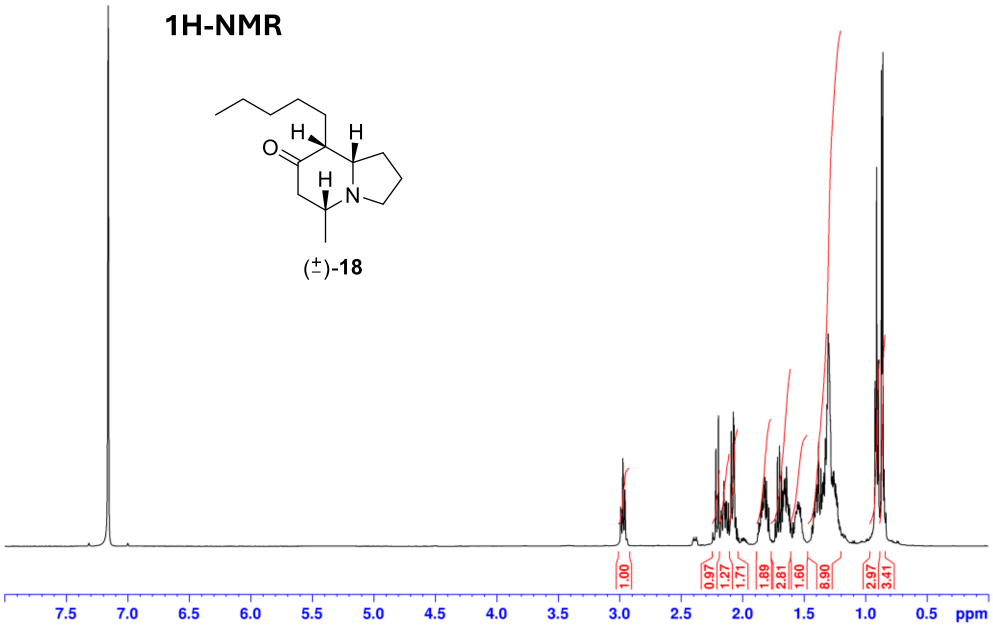
1H-NMR
H
H
O
H
N
(±)-18
1.00
0.97
1.27
1.71
1.89
2.81
1.60
8.90
2.97
3.41
7.5 7.0 6.5 6.0 5.5 5.0 4.5 4.0 3.5 3.0 2.5 2.0 1.5 1.0 0.5 ppm

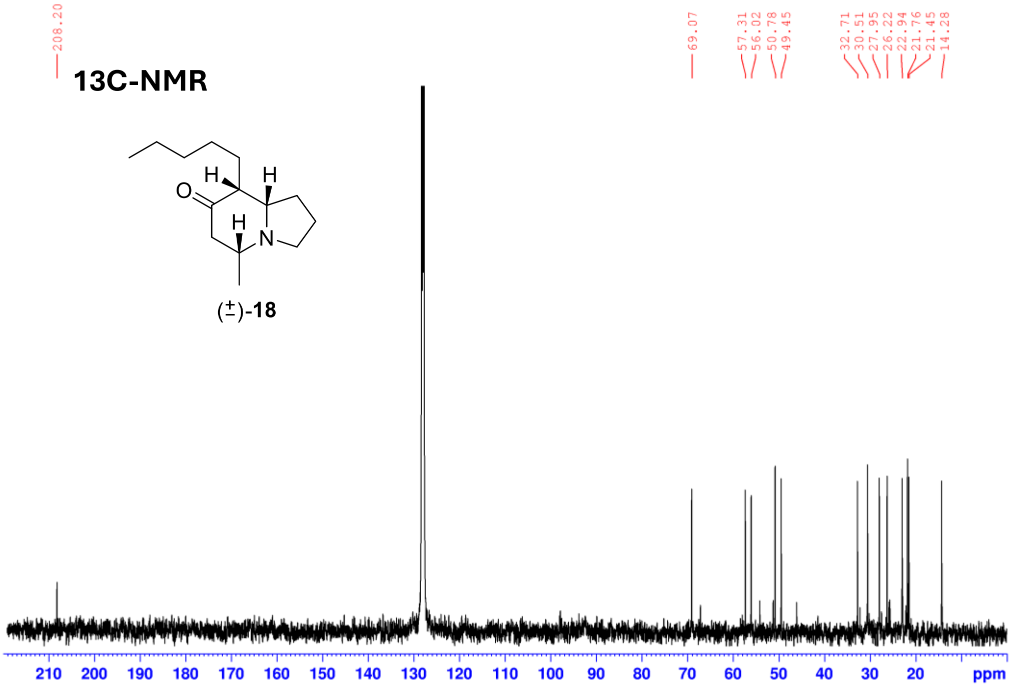
208.20
69.07
57.31
56.02
50.78
49.45
32.71
30.51
27.95
26.22
22.94
21.76
21.45
14.28
13C-NMR
H
H
O
H
N
(±)-18
210 200 190 180 170 160 150 140 130 120 110 100 90 80 70 60 50 40 30 20 ppm

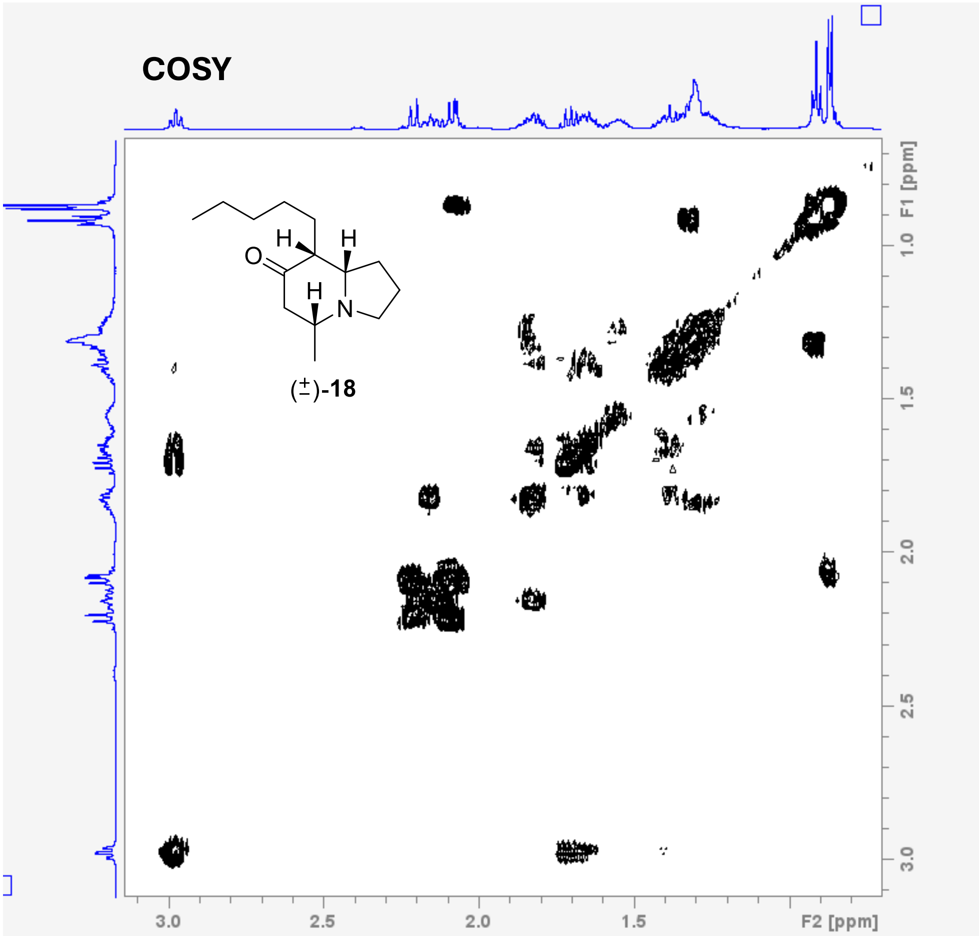
COSY
H
H
O
H
N
(±)-18
F1 [ppm]
1.0
1.5
2.0
2.5
3.0
F2 [ppm]

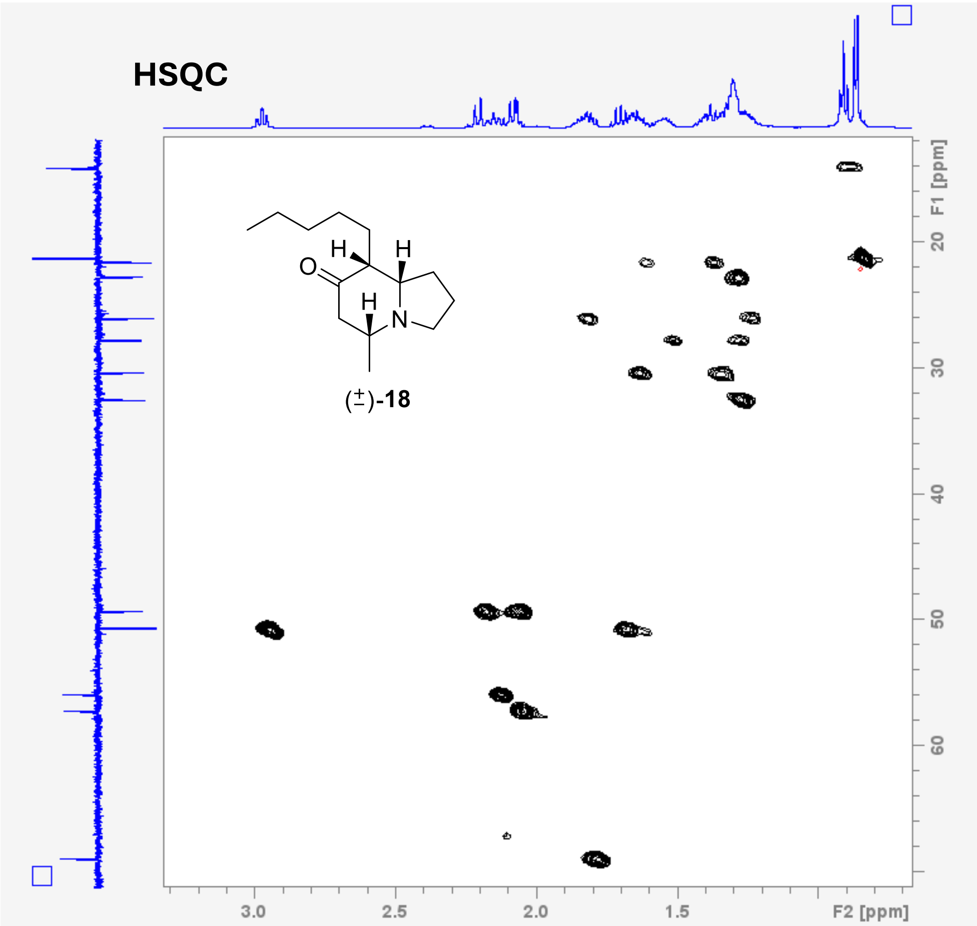
HSQC
H
H
O
H
N
(±)-18
F1 [ppm]
20
30
40
50
60
3.0
2.5
2.0
1.5
F2 [ppm]

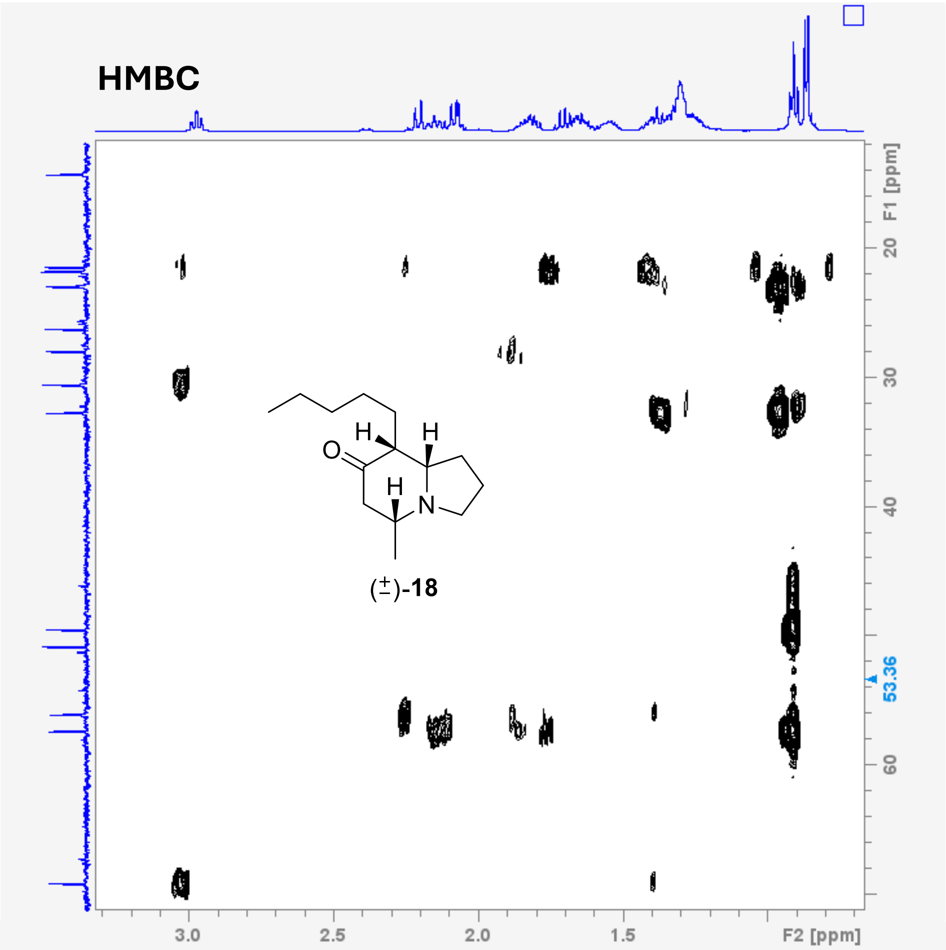
HMBC
(±)-18
F1 [ppm]
F2 [ppm]
53.36

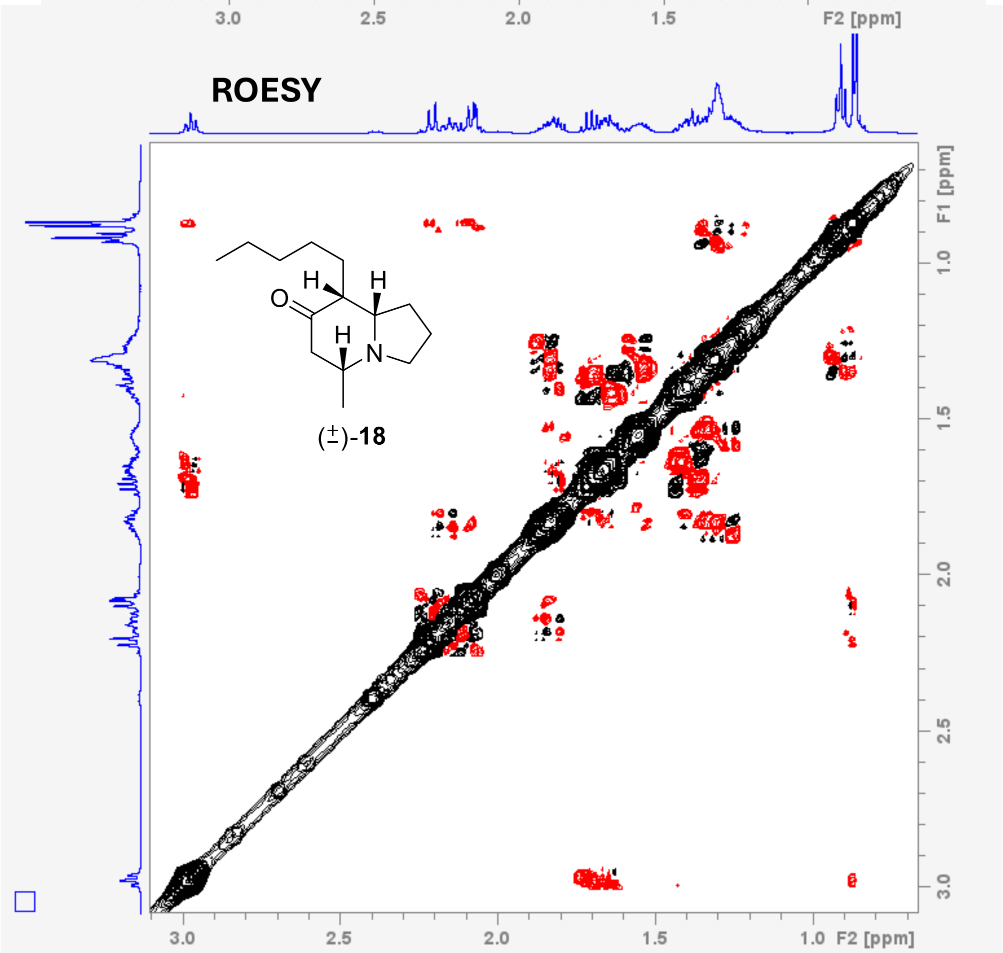
ROESY
(±)-18
F1 [ppm]
F2 [ppm]

1H-NMR

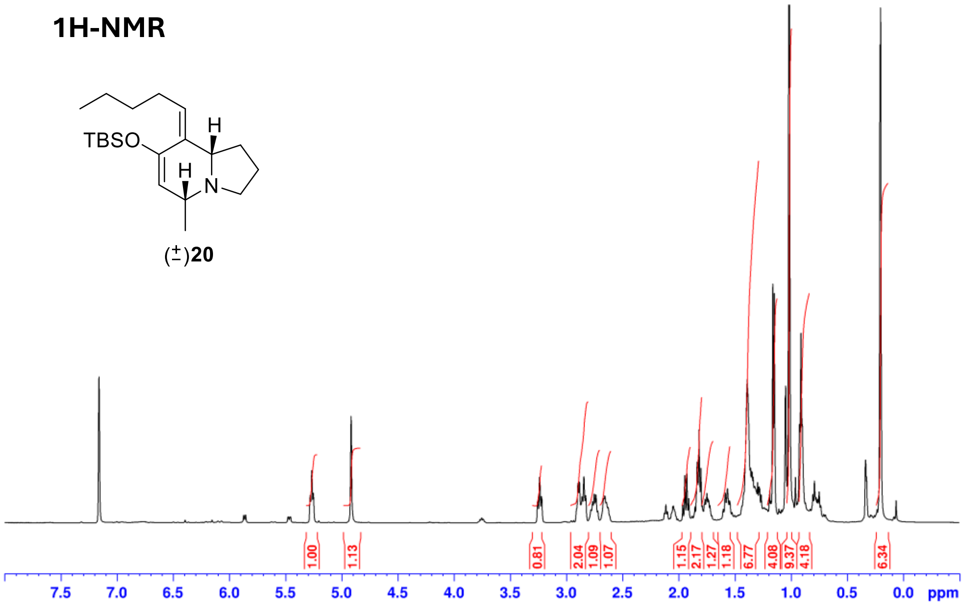

13C-NMR

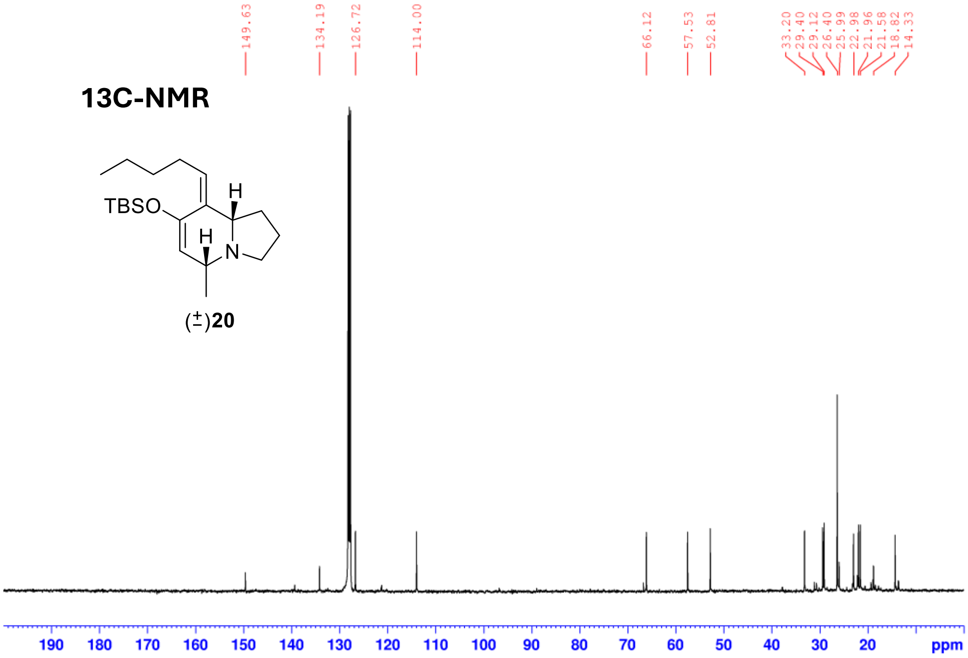

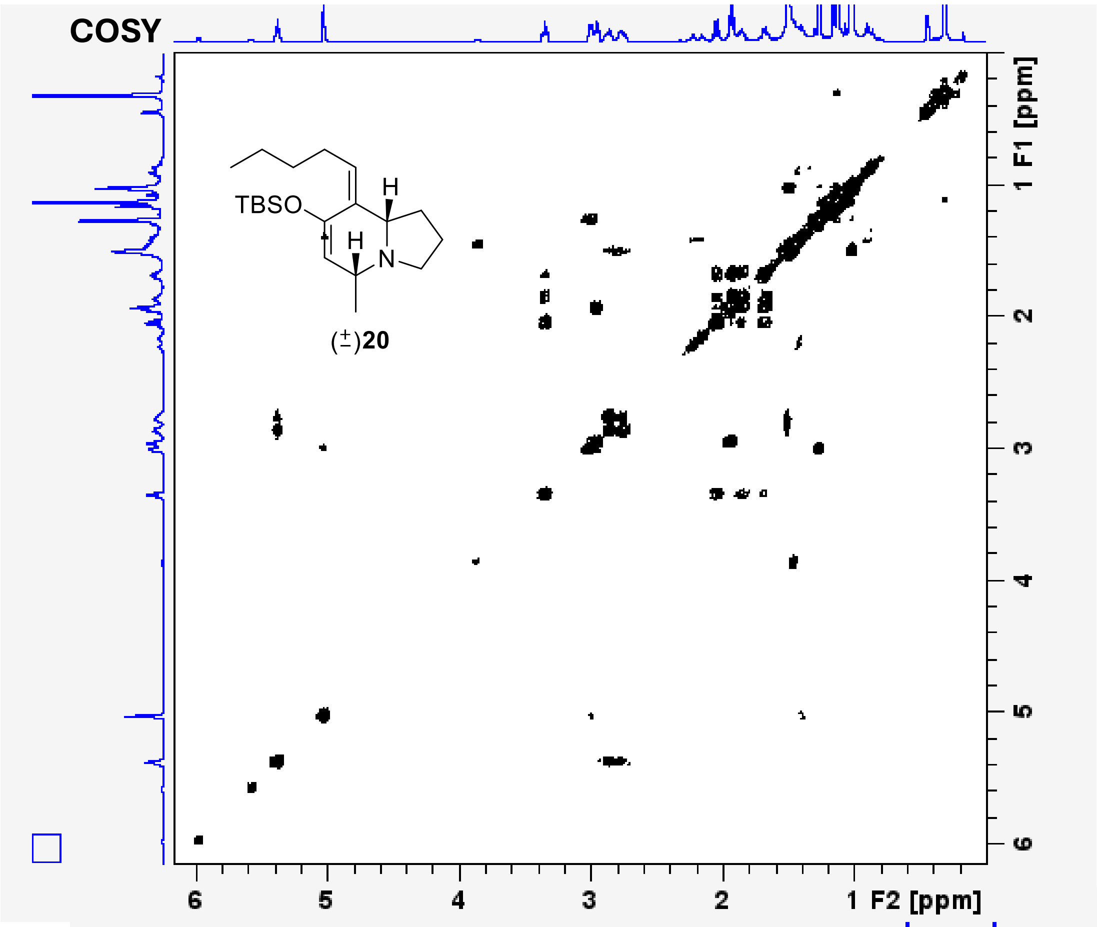
COSY
TBSO
H
H
N
(±)20
F1 [ppm]
F2 [ppm]

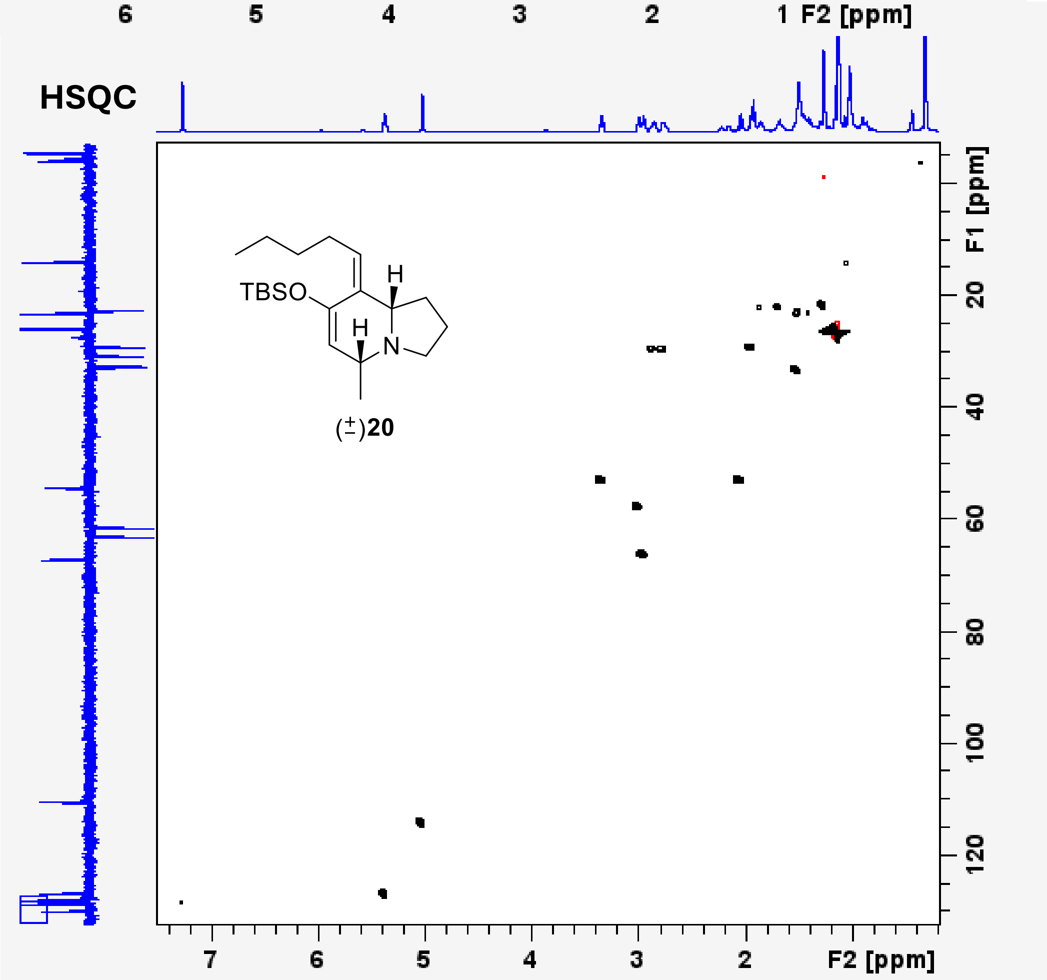
HSQC
TBSO
H
H
N
(±)20
F1 [ppm]
F2 [ppm]

1H-NMR

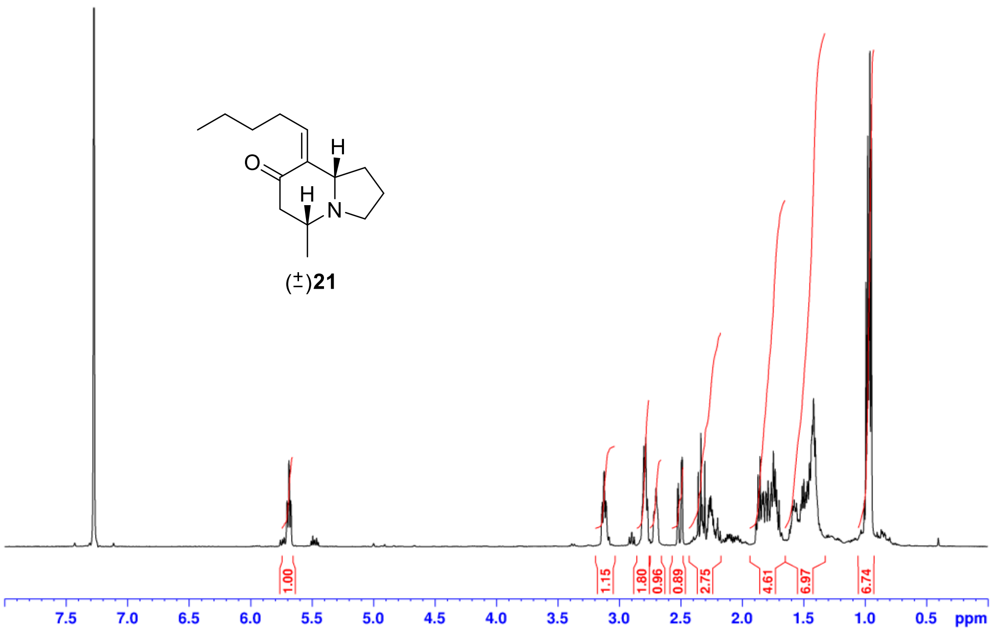

13C-NMR

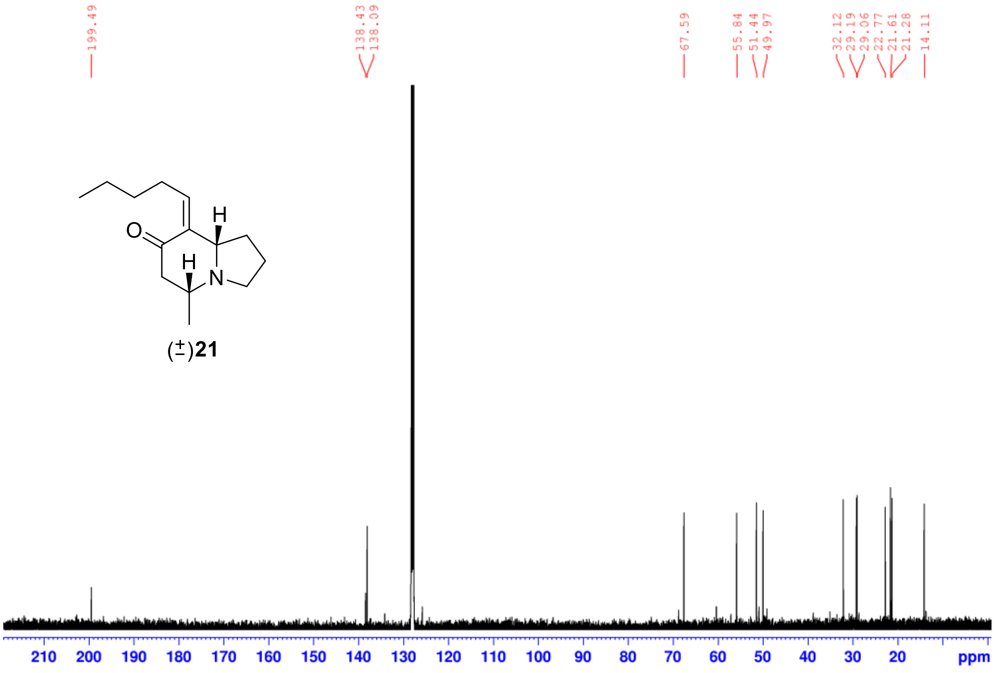

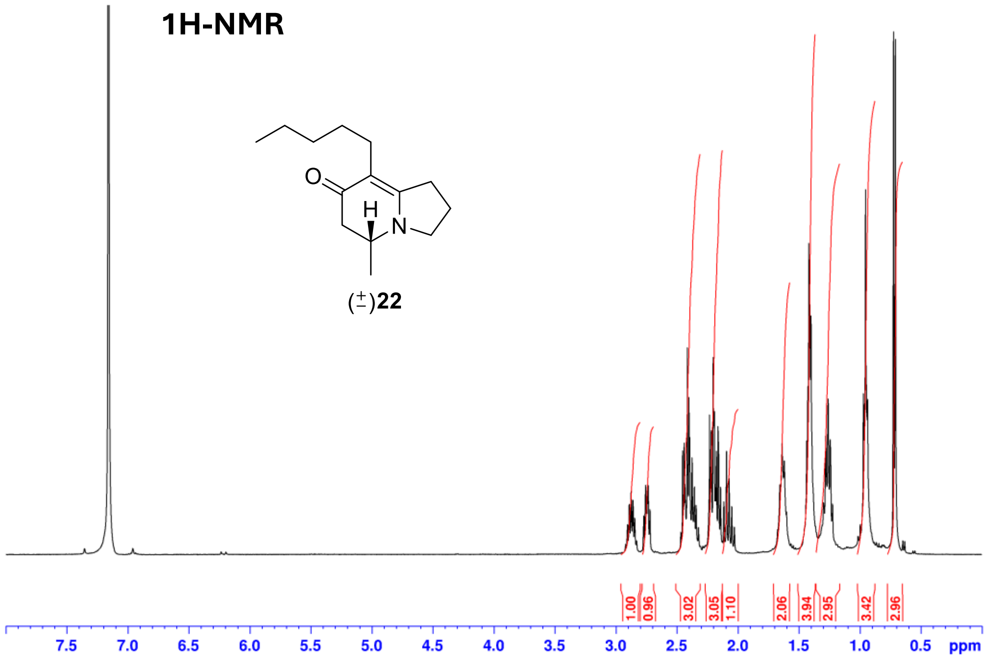
1H-NMR
O
H
N
(±)22
1.00
0.96
3.02
3.05
1.10
2.06
3.94
2.95
3.42
2.96
7.5 7.0 6.5 6.0 5.5 5.0 4.5 4.0 3.5 3.0 2.5 2.0 1.5 1.0 0.5 ppm

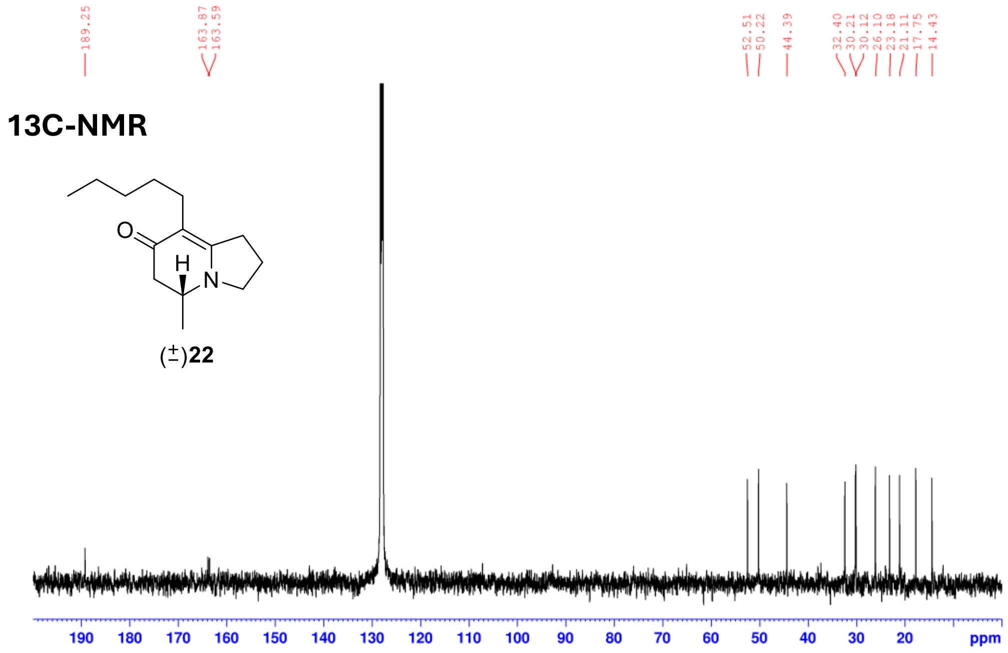
189.25
163.87
163.59
52.51
50.22
44.39
32.40
30.21
30.12
26.10
23.18
21.11
17.75
14.43
13C-NMR
O
H
N
(±)22
190 180 170 160 150 140 130 120 110 100 90 80 70 60 50 40 30 20 ppm

1H-NMR

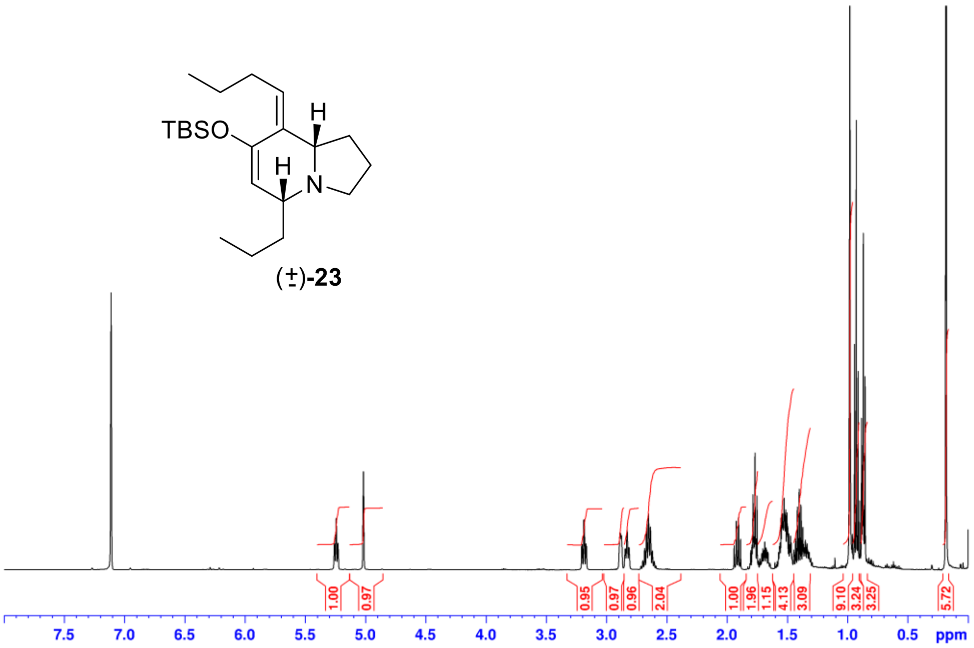

13C-NMR

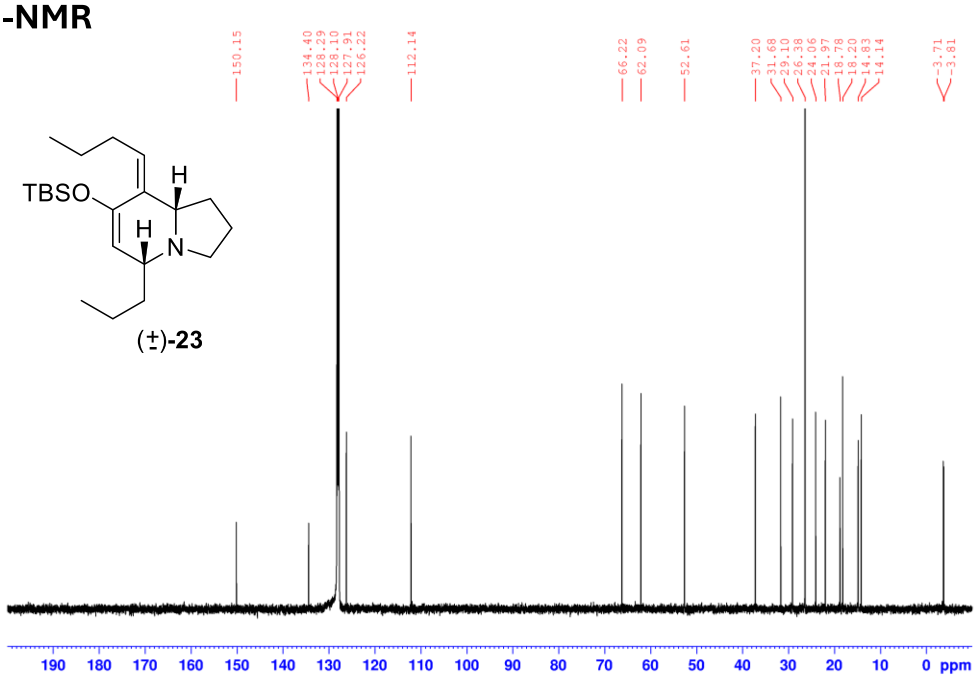

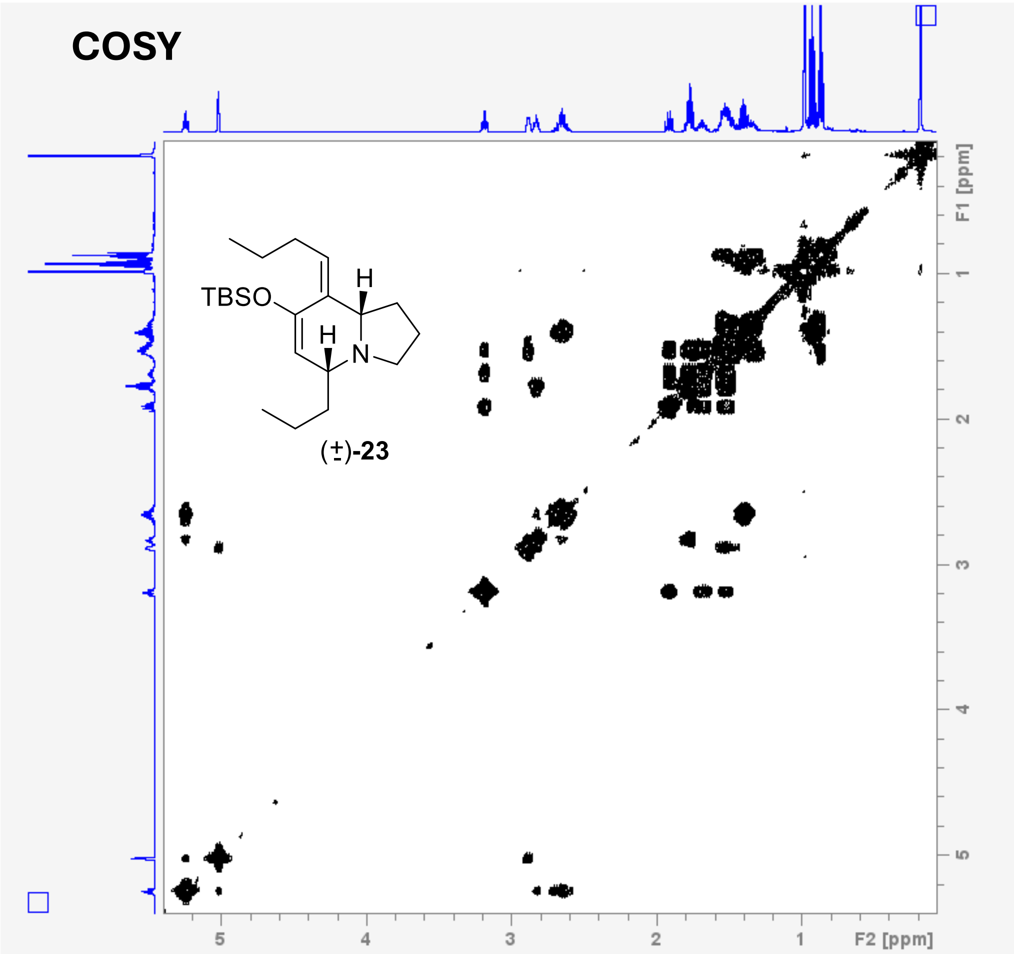
COSY
TBSO
H
H
N
(±)-23
F1 [ppm]
F2 [ppm]
1
2
3
4
5

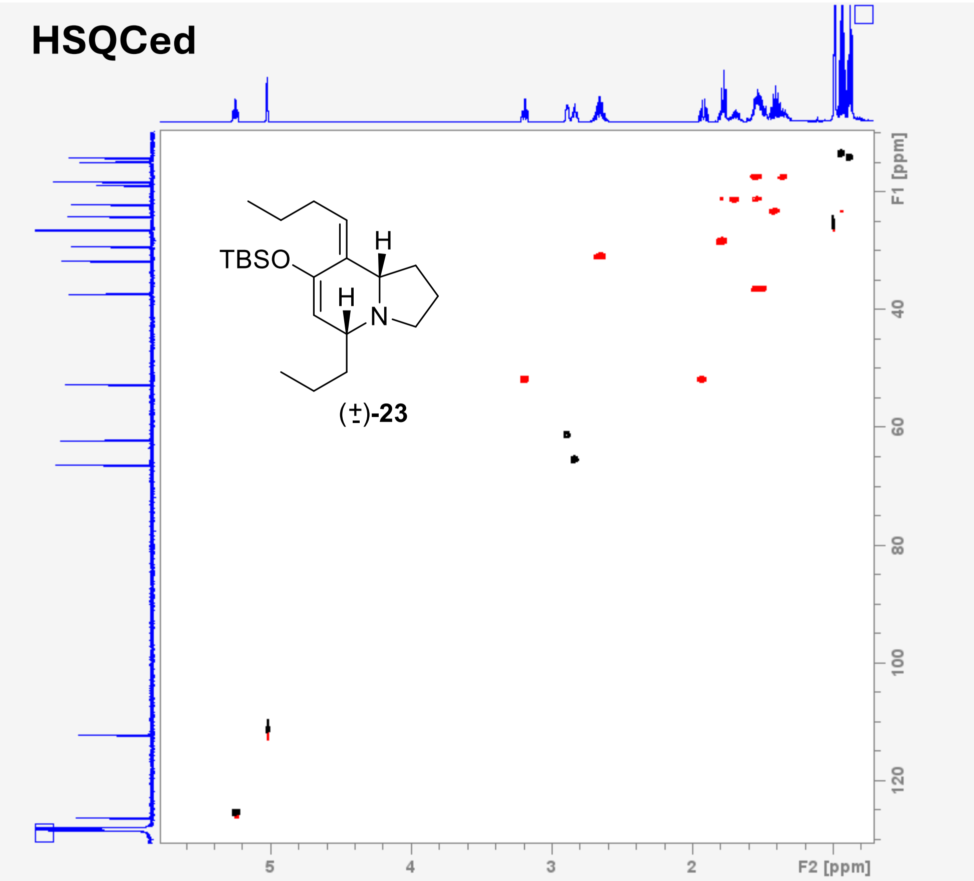
HSQCed
TBSO
H
H
N
(±)-23
F1 [ppm]
F2 [ppm]
40
60
80
100
120
5
4
3
2

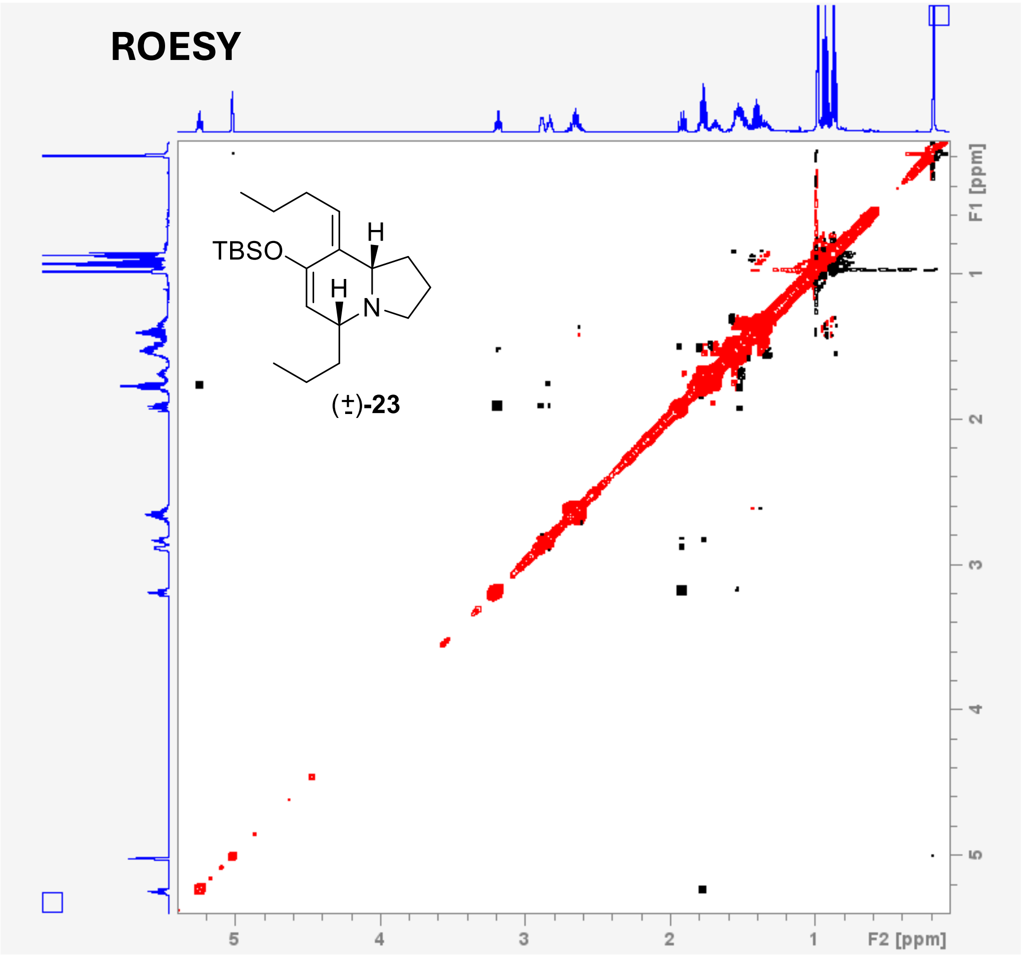

ROESY
TBSO
H
H
N
(±)-23
F1 [ppm]
F2 [ppm]
5
4
3
2
1

1H-NMR

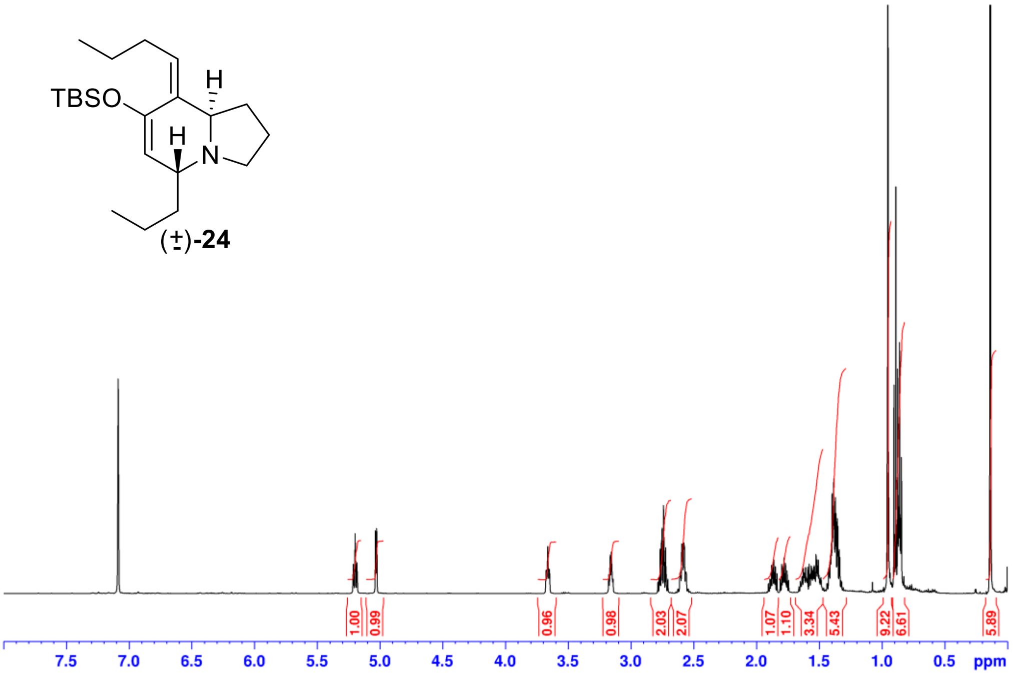

13C-NMR

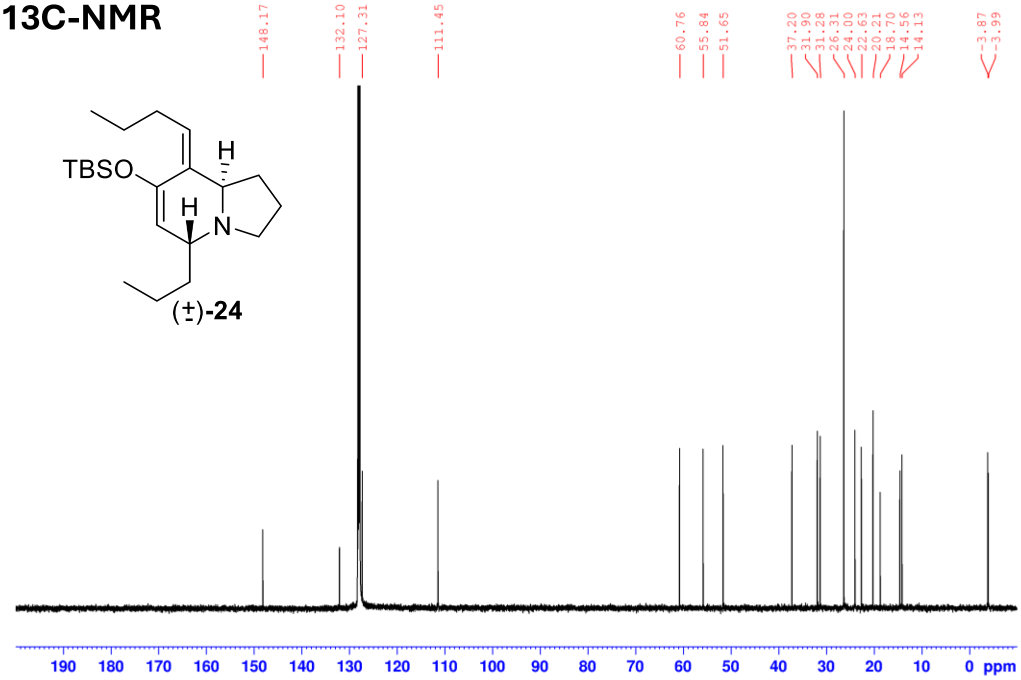

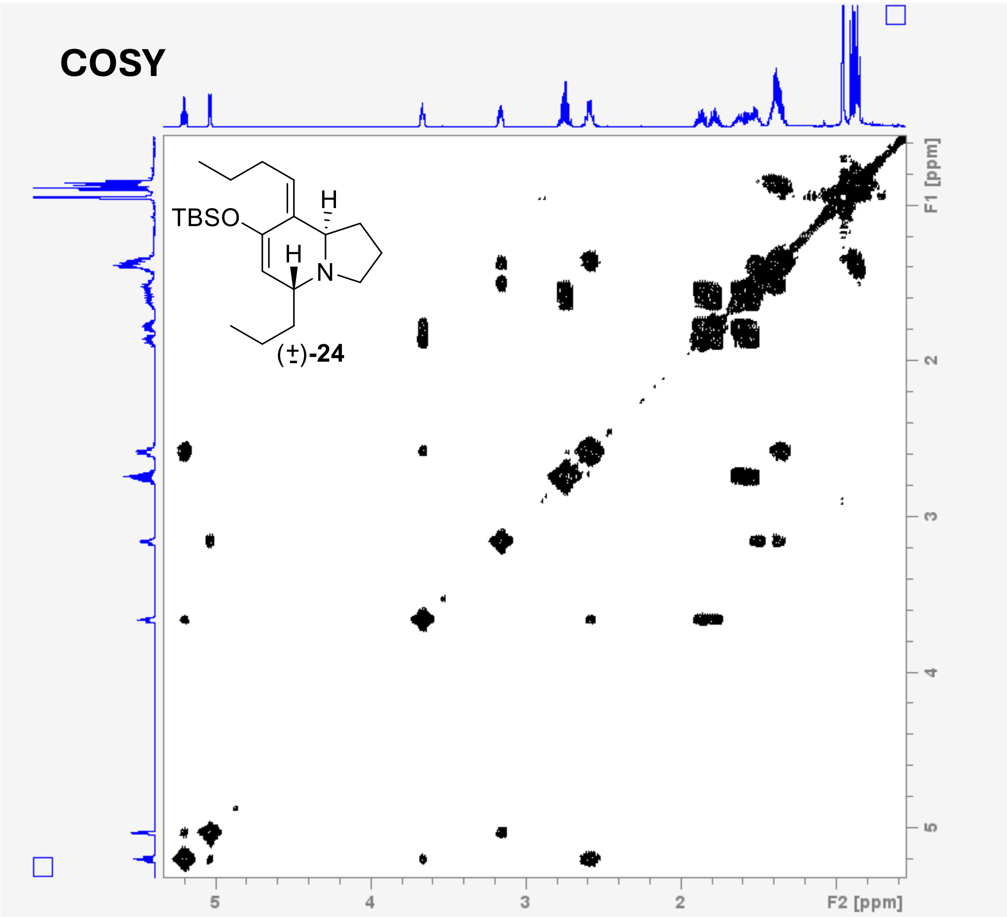
COSY
TBSO
H
H
N
(±)-24
F1 [ppm]
F2 [ppm]
5
4
3
2

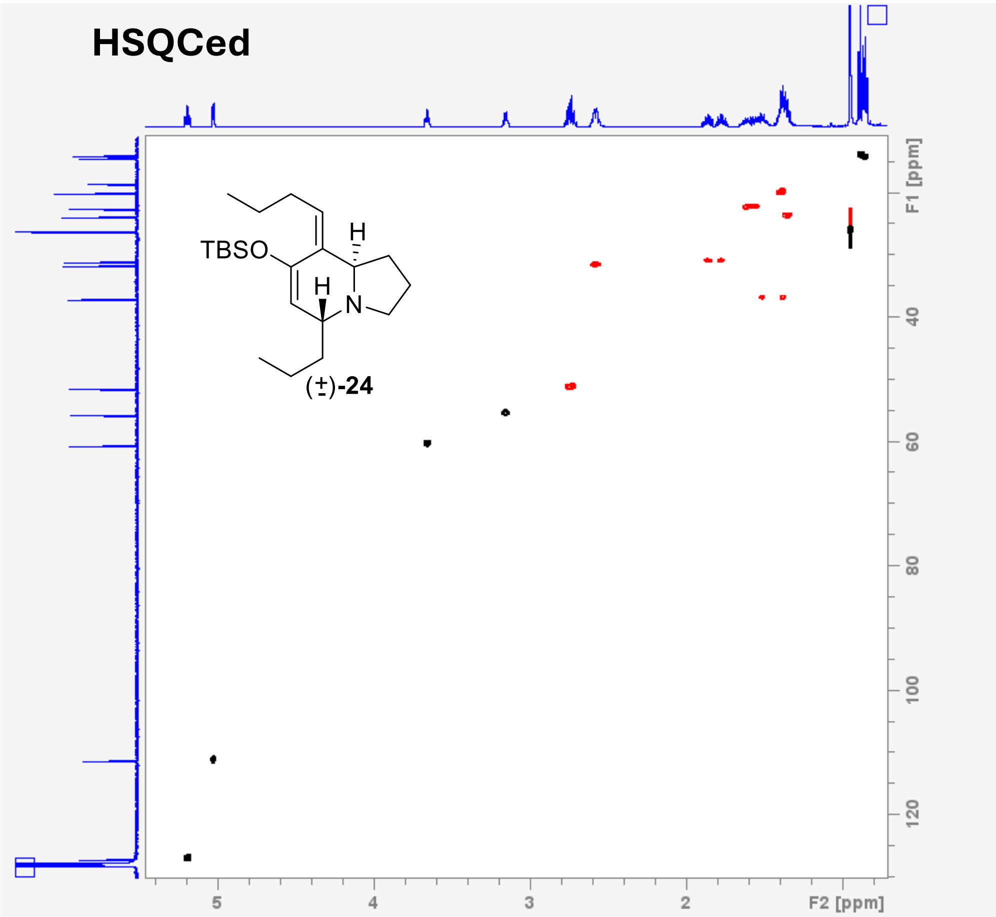
HSQCed
TBSO
H
H
N
(±)-24
F1 [ppm]
F2 [ppm]
40
60
80
100
120
5
4
3
2

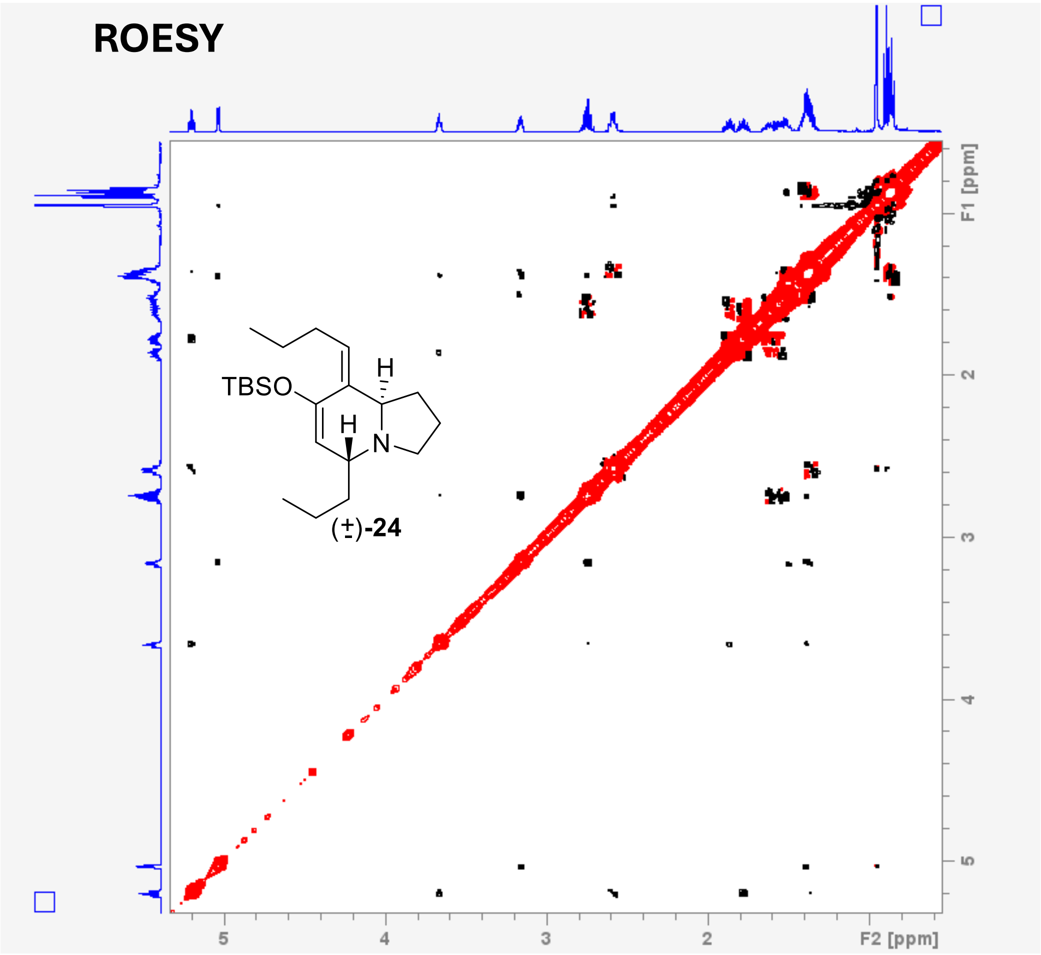
ROESY
TBSO
H
H
N
(±)-24
F1 [ppm]
F2 [ppm]
5
4
3
2

1H-NMR

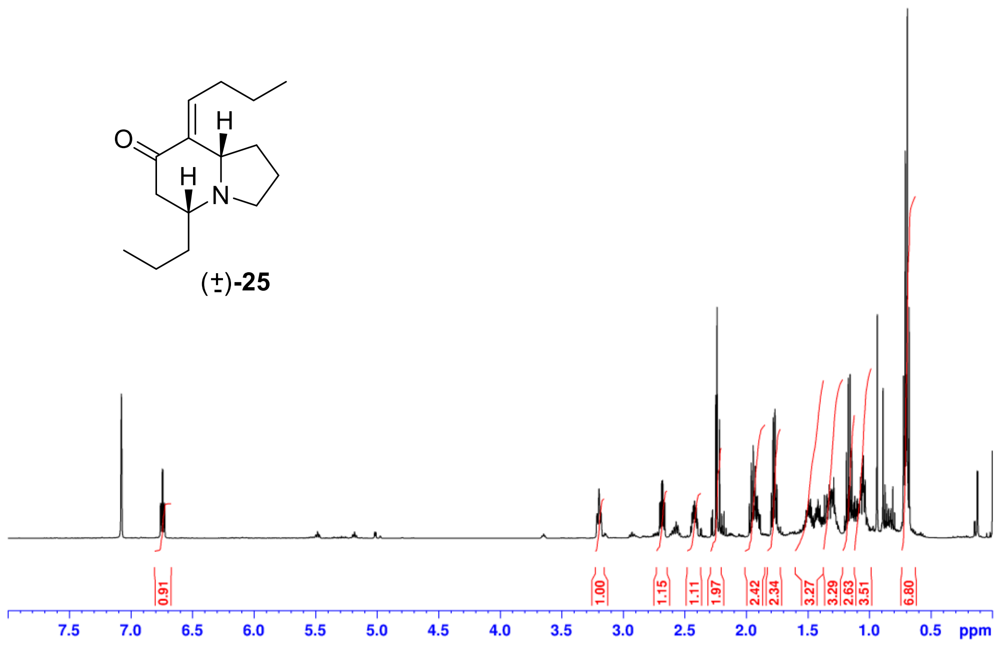

13C-NMR

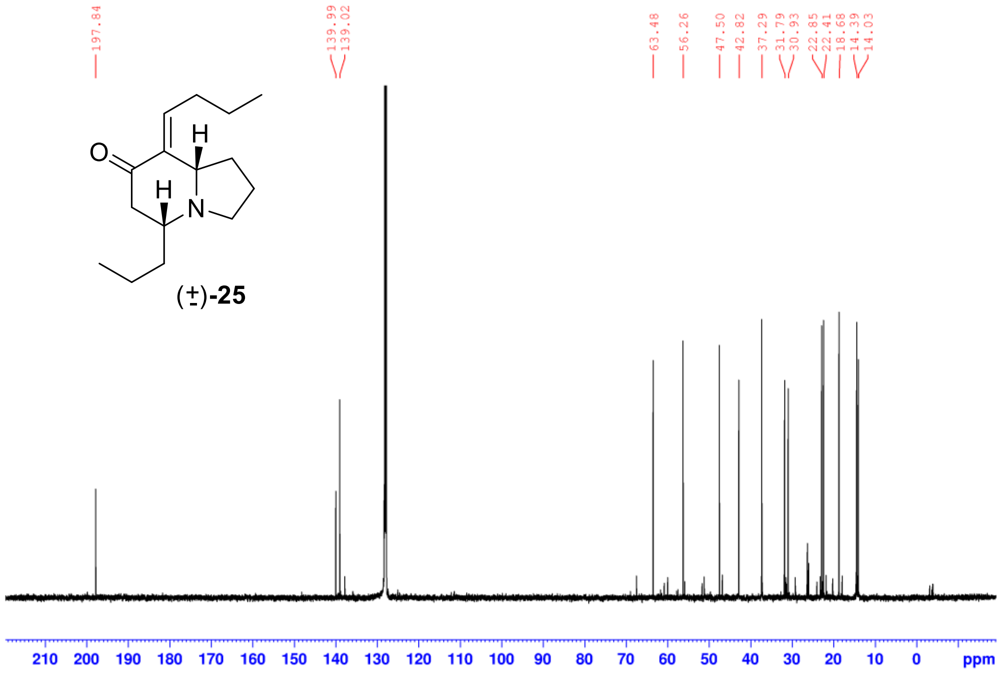

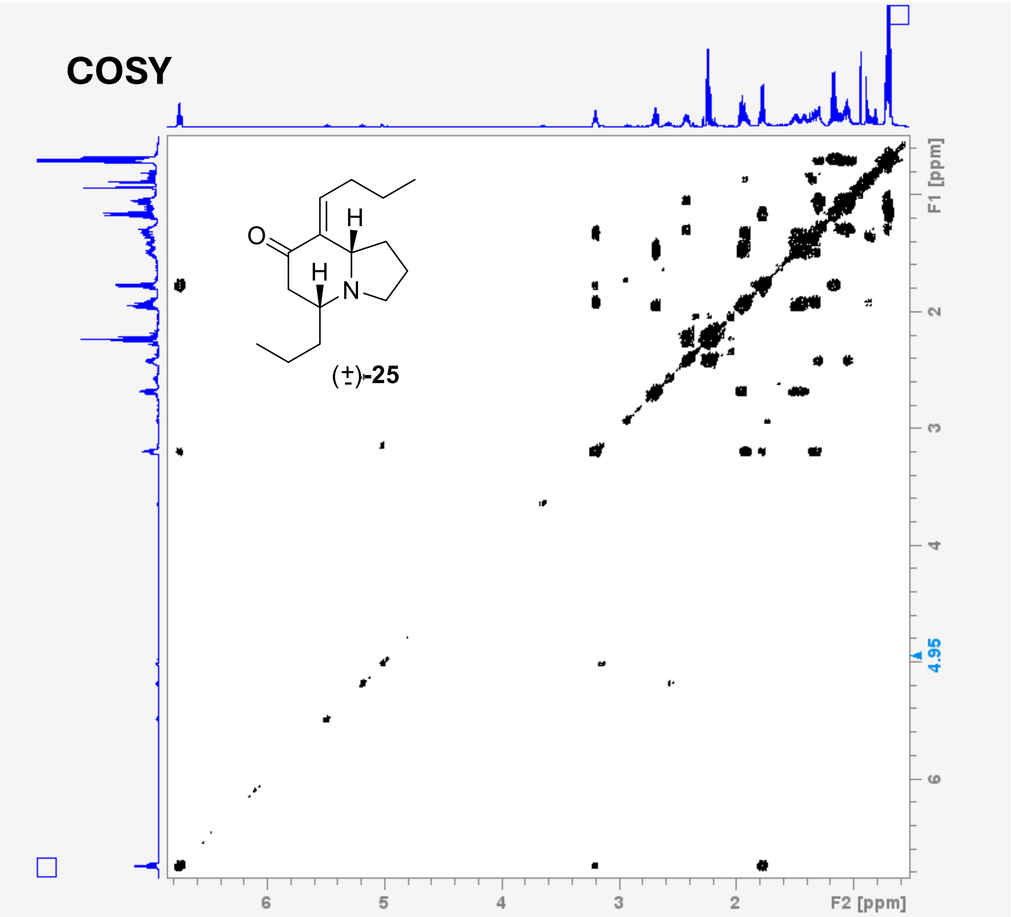
COSY
(±)-25
F1 [ppm]
F2 [ppm]
4.95

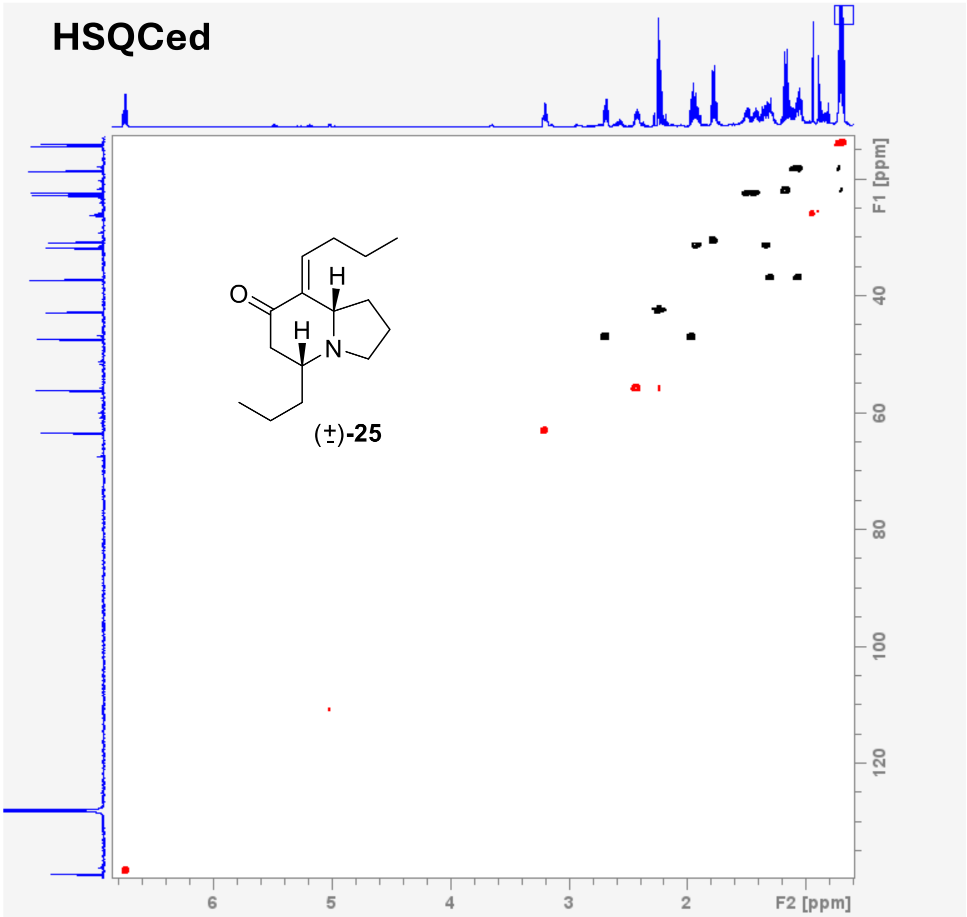
HSQCed
(±)-25
F1 [ppm]
F2 [ppm]

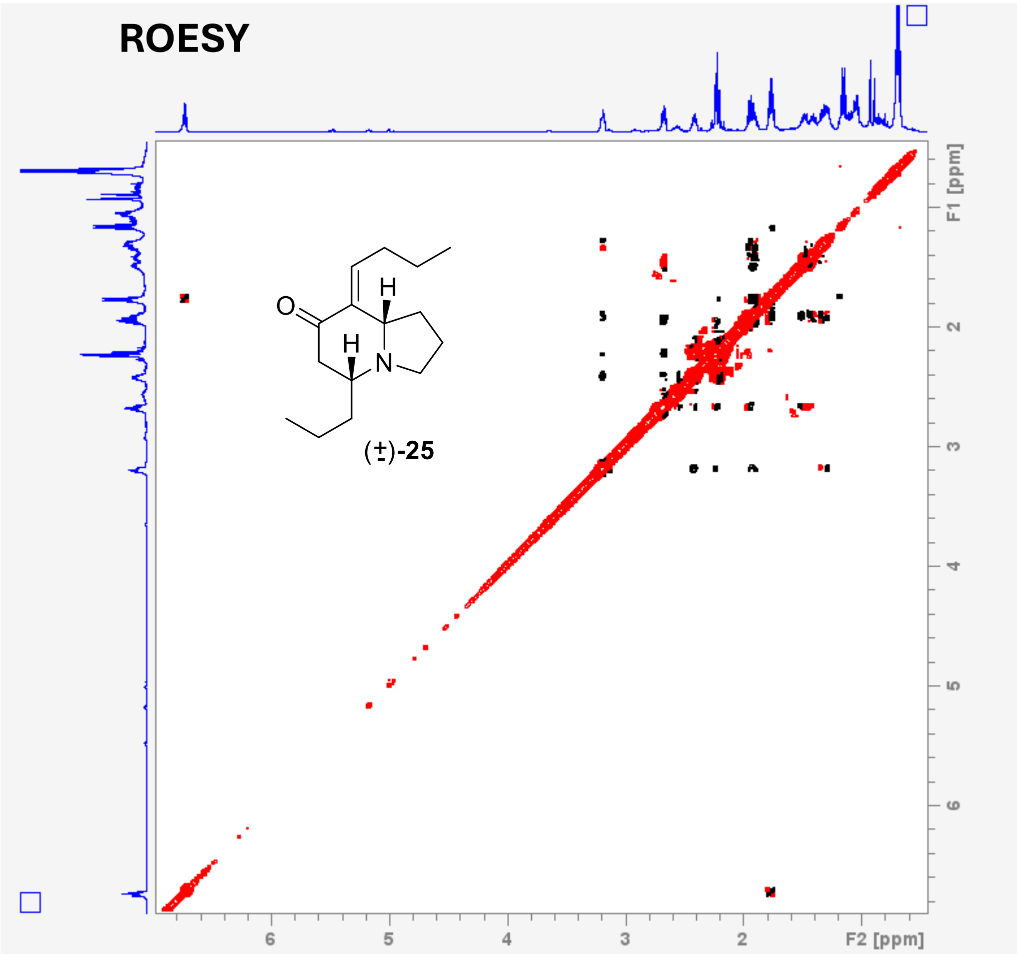

ROESY
O
H
H
N
(±)-25
F1 [ppm]
2
3
4
5
6
F2 [ppm]

1H-NMR

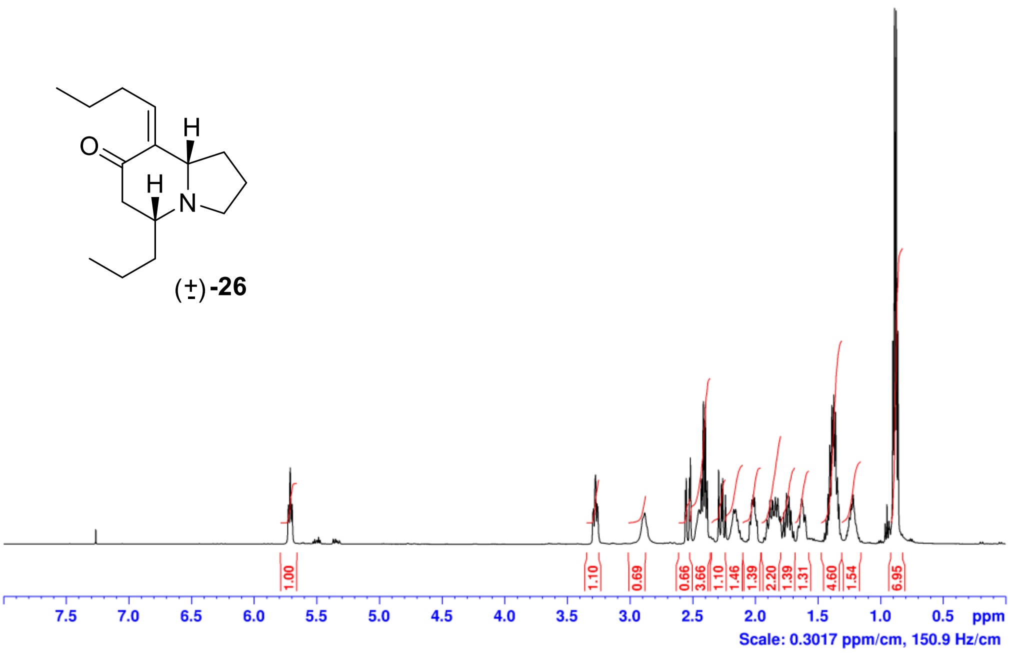

13C-NMR

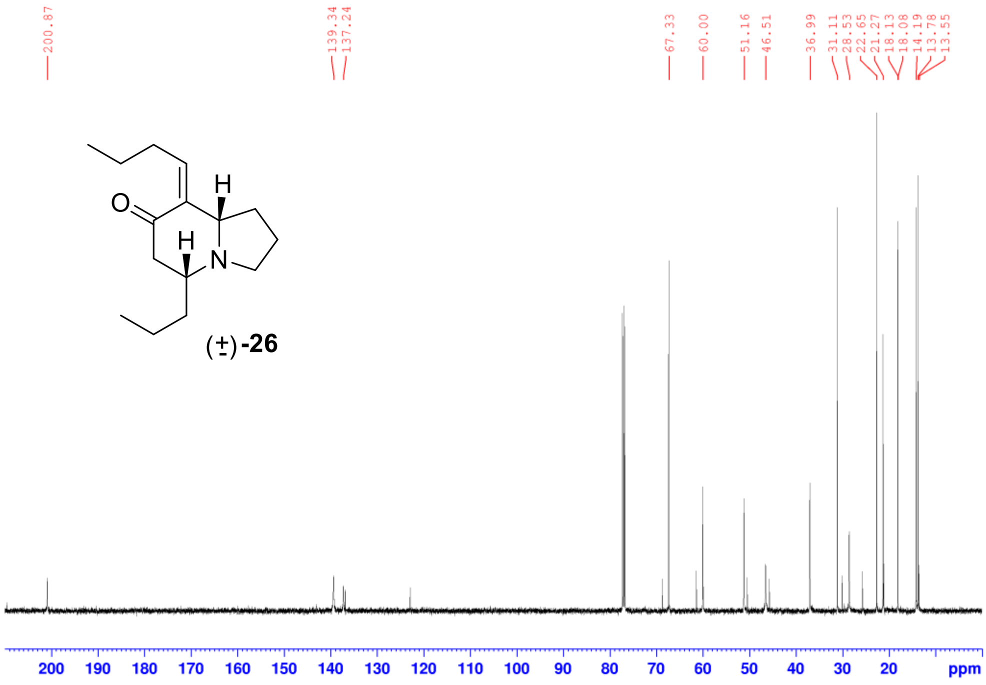

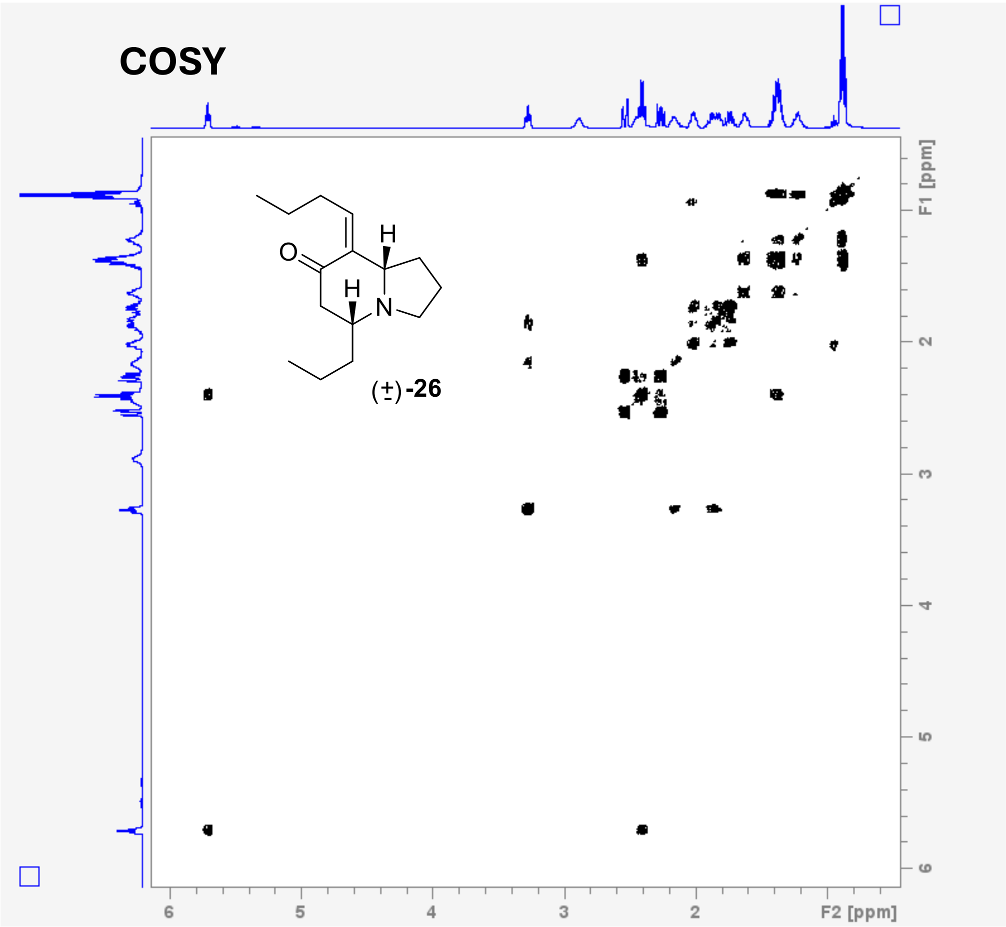
COSY
(±)-26
F1 [ppm]
F2 [ppm]

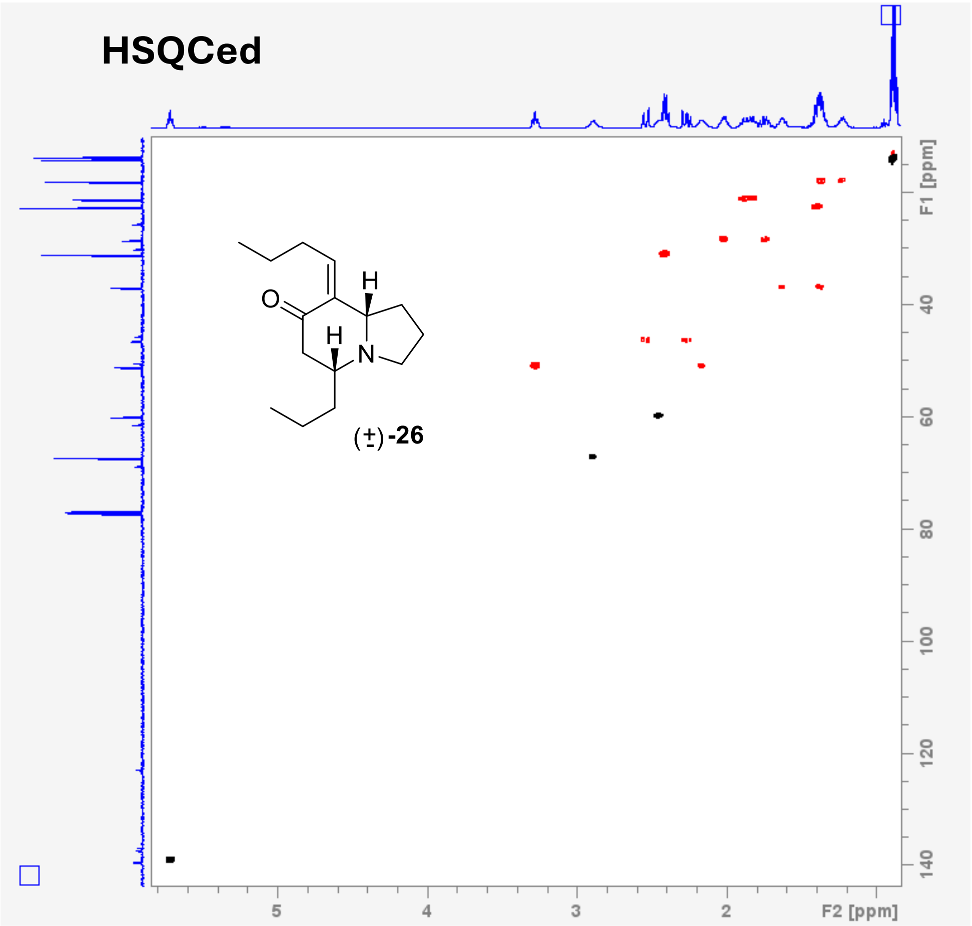
HSQCed
(±)-26
F1 [ppm]
F2 [ppm]

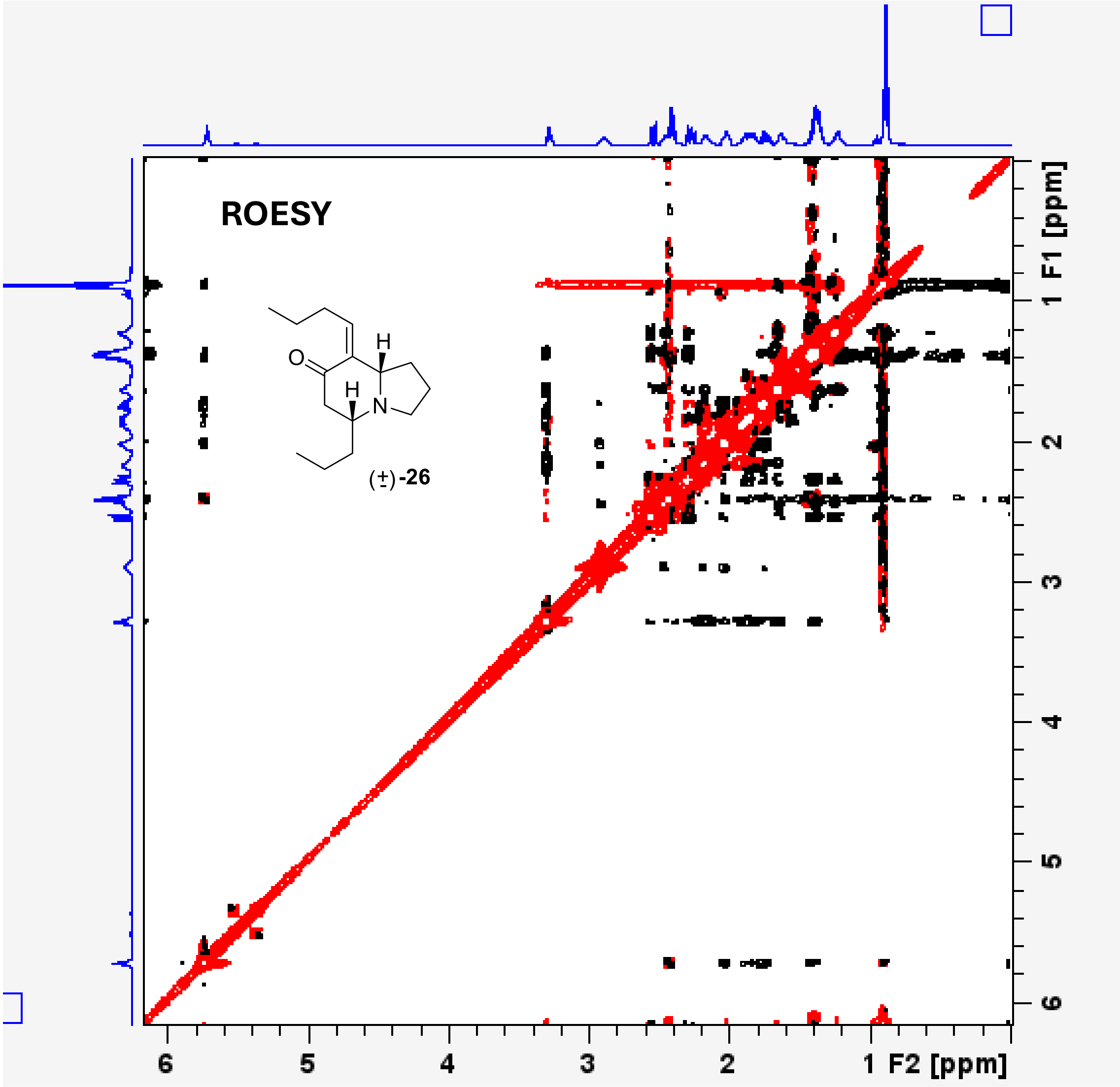

19. Neese, F. Software update: The ORCA program system, version 6.0. *WIRES Comput. Molec. Sci*. **2025**, *15*, e70019. https://doi.org/10.1002/wcms.70019.
24. Legault, C.Y. *CYLview 1.0b*; Université de Sherbrooke: Sherbrooke, QC, USA, 2009. Available online: http://www.cylview.org (accessed on 5 November 2025).
